# Archipelago architecture and evolution of paclitaxel biosynthesis revealed by the *Pseudotaxus chienii* haplotype-resolved genome

**DOI:** 10.1038/s41467-026-77115-w

**Published:** 2026-08-26

**Authors:** Huan Wang, Haidong Chen, Yaping Sun, Meifeng Su, Yi Zhang, Wangyang Xie, Ran Du, Yugeng Liu, Yuchen Xu, Shengchao Liu, Bin Jiang, Shaoxing Bai, Yanchun Peng, Lianming Gao, Jiaming Li, Jianbin Yan

**Affiliations:** 1 Shenzhen Branch, Guangdong Laboratory for Lingnan Modern Agriculture, Key Laboratory of Synthetic Biology, Ministry of Agriculture and Rural Affairs, Advanced Synthetic Biology Institute at Hohhot, Agricultural Genomics Institute at Shenzhen, Chinese Academy of Agricultural Sciences, Shenzhen, China; 2 Guangdong Province Key Laboratory of Microbial Signals and Disease Control, Integrative Microbiology Research Centre, and College of Plant Protection, South China Agricultural University, Guangzhou, China; 3 School of Life Sciences, Henan University, Kaifeng, China; 4 Shenzhen Research Institute of Henan University, Shenzhen, China; 5 MOE Key Laboratory of Bioinformatics, Tsinghua-Peking Center for Life Sciences, School of Life Sciences, Tsinghua University, Beijing, China; 6 Center for Interdisciplinary Biodiversity Research & College of Forestry, Shandong Agricultural University, Tai’an, China

**Keywords:** Plant evolution, Genome, Secondary metabolism, Metabolic pathways

## Abstract

*Pseudotaxus chienii*, the only species in the genus *Pseudotaxus*, is closely related to *Taxus* genus. Whether *P. chienii* can produce paclitaxel has long remained controversial. Here, we present a chromosome-level, haplotype-resolved genome of *P. chienii*, with each haplotype ( ~ 15.4 Gb) exhibiting high continuity and completeness. We identify a structurally conserved yet physically dispersed genomic region, termed the Taxane Biosynthetic Archipelago (TBA), containing three biosynthetic gene clusters and collinear core genes essential for taxane biosynthesis. Integrative transcriptomic, metabolite, and heterologous enzymatic analyses reveal that while *P. chienii* retains a largely conserved genomic architecture associated with paclitaxel biosynthesis, its terminal pathway shows severe functional degradation and may have largely lost the functional capacity for paclitaxel production. Concurrently, it exhibits significant accumulation of taxinine J. This study provides an evolutionary framework for interpreting the long-standing debate on paclitaxel production beyond *Taxus*, and understanding the evolutionary decay of complex specialized pathways.

## Abstract (web summary)

Paclitaxel biosynthesis beyond *Taxus* has long been debated. Here, the authors report genome assembly of *Pseudotaxus chienii* and show that, while it retains a largely conserved genomic architecture for paclitaxel biosynthesis, extensive degradation of the terminal pathway suggests a substantial loss of paclitaxel-producing capacity.

## Introduction

*Pseudotaxus chienii*, a rare endemic understory conifer, is characterized by seeds enclosed in a white fleshy aril, distinct from the typically red arils of *Taxus* (Supplementary Fig. 1)^1^. It occupies moist rocky habitats in subtropical montane forests at 600–1500 m elevation, with fragmented distributions confined to isolated populations in southeastern China. Due to habitat fragmentation, it is on the International Union for Conservation of Nature (IUCN) vulnerable and endangered list^2,3^. As the sole species in the monotypic genus *Pseudotaxus* (*Taxaceae*)^4^, it serves as an important model for understanding the evolutionary diversification of *Taxaceae*.

In particular, the phylogenetic and morphological similarities between *P. chienii* and *Taxus* make it a suitable option for comparative studies on taxoid metabolism. *Taxus* species yield paclitaxel (Taxol), a vital diterpenoid for cancer chemotherapy and among the most studied plant specialized metabolites^5,6^. The paclitaxel biosynthesis entails clustered, co-regulated enzymes, including cytochrome P450s, acyltransferases, and hydroxylases. Genomic analyses in *Taxus mairei*^7^, *T. wallichiana*^8^, and *T. yunnanensis*^9^ have uncovered novel taxadiene synthases, conserved biosynthetic gene clusters, and provided evolutionary insights. Despite these findings, gymnosperms generally possess exceptionally large and highly repetitive genomes, posing substantial challenges for resolving their structural organization at high resolution. Consequently, it remains poorly understood how their complex, multi-step specialized metabolic pathways in gymnosperms preserve genomic architectural integrity while simultaneously undergoing evolutionary diversification. Thus, *P. chienii* represents an ideal model to dissect the origin, genomic architectural conservation, and evolutionary divergence of the paclitaxel biosynthetic pathway across *Taxaceae*.

Although significant progress has been made in elucidating its biosynthetic pathway in *Taxus* species^7–13^, research on paclitaxel biosynthesis in *P. chienii* is still limited and presents conflicting results. Previous studies report conflicting paclitaxel levels in *P. chienii*, with none detected by Heinig et al.^14^ but substantial accumulation noted by Yu et al.^15^, highlighting the need for comprehensive genomic investigation to resolve these discrepancies. A recent study by Wang et al.^16^ further intensified this controversy by proposing that *P. chienii* possesses only an incomplete taxane biosynthetic pathway, which terminates before the synthesis of 10-deacetylbaccatin Ⅲ. However, conclusions derived from conventional consensus genome assemblies may be inherently limited in highly heterozygous species. Accurate characterization of complex specialized metabolic pathways requires haplotype-resolved genome assemblies, which enable comprehensive characterization of allelic variation, structural divergence, and intact pathway organization^17,18^. Moreover, the metabolic capacity of ancient gymnosperm lineages may not be completely lost at the structural level; instead, it may have undergone substantial functional attenuation, with the resulting metabolic variation potentially shaped by complex genotype-by-environment (G × E) interactions^19,20^. Together, these considerations underscore the urgent need for integrated haplotype-resolved genomic analyses combined with targeted metabolite quantification to accurately elucidate the evolutionary and functional status of paclitaxel biosynthesis in *P. chienii*.

Here, we present a chromosome-level, haplotype-resolved genome assembly of *P. chienii* and identify a genomic architecture underlying paclitaxel biosynthesis, which we designate the Taxane Biosynthetic Archipelago (TBA). The TBA comprises multiple physically dispersed yet syntenically conserved biosynthetic gene groups and core pathway enzymes. Integrating this genomic framework with targeted metabolite quantification, transcriptomic analyses, and heterologous enzyme assays, we show that although *P. chienii* retains a largely conserved genomic architecture associated with paclitaxel biosynthesis, its terminal pathway exhibits severe functional degradation. This attenuation is characterized by reduced catalytic efficiency of PcTOT and the failure of multiple downstream enzymes to produce detectable pathway products in *Nicotiana benthamiana*. Paclitaxel is detected only at trace levels under specific conditions, whereas taxinine J and taxusin accumulate to substantial levels in specific tissues and populations. This study provides a framework for understanding the evolutionary decay and diversification of complex specialized metabolic pathways in *Taxaceae*. Furthermore, the haplotype-resolved assembly offers a valuable resource for species conservation and enables future metabolic engineering of high-value taxanes.

## Results

### The de novo assembly of haplotype-resolved genomes of *Pseudotaxus chienii*

To generate a high-quality genome assembly for *P. chienii*, we first conducted a genome survey. The *P. chienii* genome size was estimated using flow cytometry and K-mer analysis. Flow cytometry suggested a size of approximately 15.64 Gb (Supplementary Fig. 2a–c and Supplementary Table 1), largely consistent with K-mer analysis (14.85 Gb) (Supplementary Fig. 2d). Furthermore, K-mer analysis showed a prominent heterozygous peak (Supplementary Fig. 2d), indicating significant genomic complexity and high heterozygosity.

**Fig. 1:**
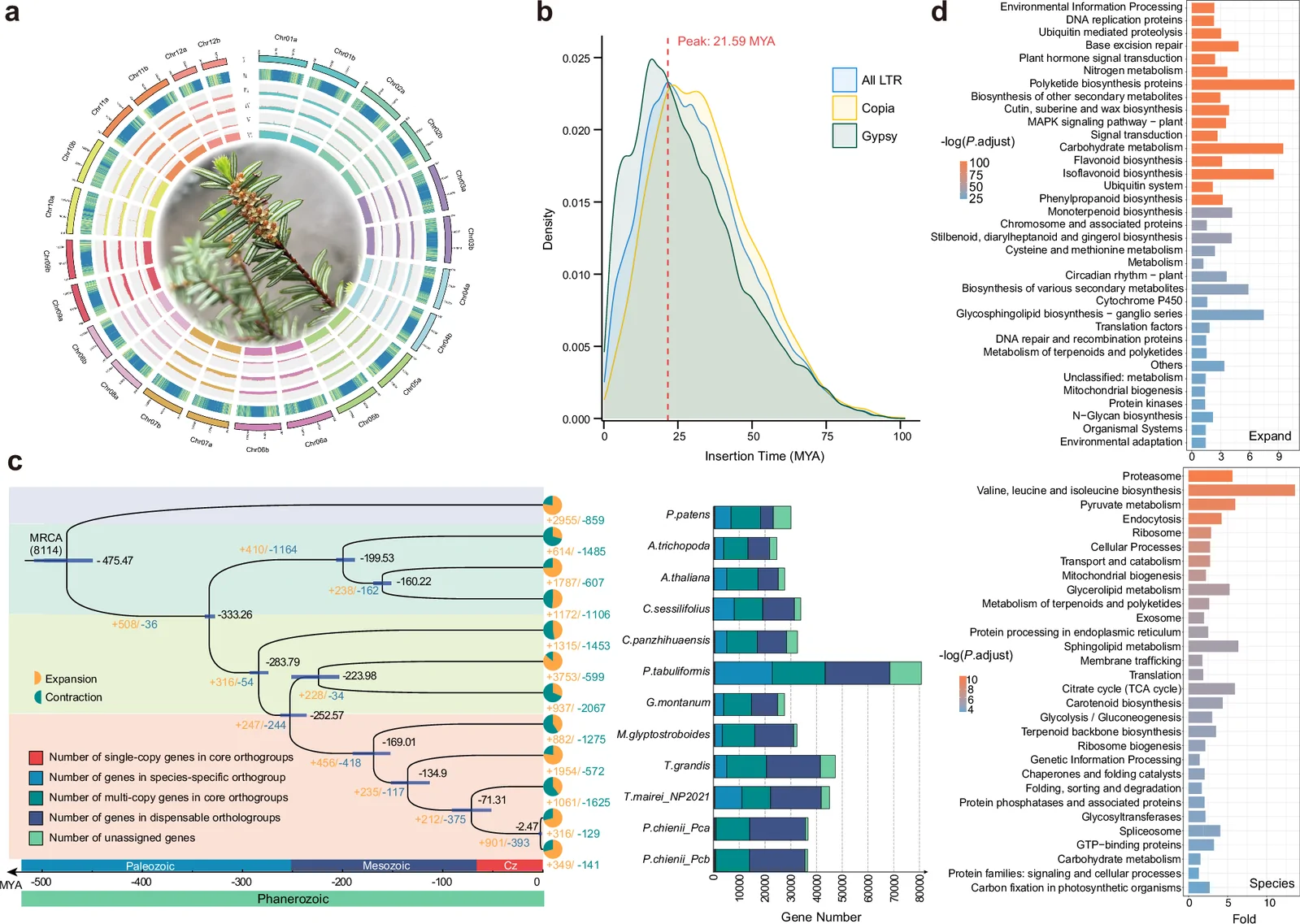
Genome characteristics and comparative genomic analysis of *Pseudotaxus chienii*. **a** Genome landscape of the 24 assembled pseudochromosomes in *P. chienii*. Track (I) displays the length of each pseudochromosome (in Mb), while tracks (II) to (VI) represent the distribution of repeat density, GC content, gene density, and the abundance of Ty3/Gypsy, Ty1/Copia, and unknown long terminal repeat (LTR) elements, respectively. All features were calculated using a 5 Mb sliding window. The central image depicts *P. chienii* during its flowering stage. **b** Expansion and diversity of LTR elements in the *P. chienii* genome. **c** Evolutionary dynamics of gene families in *P. chienii* and representative plant species. The predicted most recent common ancestor (MRCA) is estimated to have possessed 8,146 gene families. Pca/b, *P. chienii* hap1/hap2. **d** KEGG pathway enrichment analysis of expanded and species-specific genes in *P. chienii*. Color intensity reflects adjusted *P*-value significance, and the x-axis shows enrichment fold. The *P* values were calculated using a two-sided hypergeometric test and adjusted for multiple comparisons using the false discovery rate (FDR). Source data are provided as a Source Data file.

To produce a high-quality assembly for *P. chienii*, we employed a multi-platform sequencing strategy, including 99 × BGI short reads, 47 × Revio HiFi long reads, and 151.42 × Hi-C sequencing for scaffolding (Supplementary Table 2). This yielded two haplotype-phased genomes (Hap1 and Hap2), both anchored to 12 pseudochromosomes, corresponding to the highly conserved haploid chromosome number (*n* = 12) of the *Taxus* species^21,22^. The total length of the assembled pseudochromosomes is consistent with our genome size estimations, the previously published genome of *P. chienii*^16^. Hap1 spans 15.434 Gb (725 contigs; contig N50 60.2 Mb) and Hap2 spans 15.430 Gb (817 contigs; contig N50 55.9 Mb). (Fig. 1a and Supplementary Table 3). The Hi-C interaction heatmap exhibited a characteristic checkerboard pattern (Supplementary Figs. 3 and 4). Strong diagonal interaction patterns confirmed proper chromatin proximity and absence of aberrant contacts, which supported the accuracy of scaffold placement and the structural integrity of the assembly. The assembly length was highly consistent with the flow cytometry estimate and the K-mer analysis result, confirming comprehensive genome representation (Supplementary Table 3). Merqury analysis showed genome completeness of ~87.6% for individual haplotypes and 98.13% for merged assembly (Supplementary Table 3). High base-level consensus accuracy was also evidenced by base quality values (QV) exceeding 54 for both haplotypes (Supplementary Table 3).

### De novo structural (gene model) annotation

Next, we performed high-quality genome annotation, including repeat annotation, gene prediction, and functional annotation. Comprehensive repeat annotation demonstrated that repetitive sequences were the dominant genomic component, accounting for 88.43% (Hap1) and 88.60% (Hap2) of the assembly. Among these, long terminal repeat (LTR) retrotransposons were dominant (53.65% in Hap1 and 54.05% in Hap2), with Gypsy elements and Copia elements being the most dominant (Fig. 1a and Supplementary Table 4 and 5). DNA transposons represented 21.55% (Hap1) and 21.12% (Hap2) of the genome, with Tc1/IS630/Pogo and hobo-Activator superfamilies being most abundant (Supplementary Table 4 and 5). Among LTR types, younger Gypsy elements showed greater abundance and broader genomic distribution than Copia elements, consistent with their preference for heterochromatic regions, potentially facilitating genome remodeling and regulatory diversification (Supplementary Table 4). Additionally, insertion time analysis of 33605 intact LTR retrotransposons (Hap1) revealed a unimodal expansion peak at ~21.59 MYA (Fig. 1b), which calibrated at 7.3 × 10⁻¹⁰ substitutions, site, and year^23^, indicating singular burst of transpositional activity in the *P. chienii* genome. *P. chienii* harbors a larger genome (15.6 Gb vs. 10.2 Gb) yet fewer intact LTR insertions (31% vs. 40%) within the 8–24 MYA window in comparison to *T. mairei*^7^. This showed that genome expansion in *P. chienii* was driven by the enhanced retention of LTR that peaked several million years earlier than those in *T.mairei*, whereas the latter is distinguished by a slightly more recent burst coupled with more effective purging of young elements^7^. These findings suggested that the *P. chienii* genome exhibits extreme repetitive sequence expansion and structural complexity, likely resulting from extensive transposon amplification during evolutionary divergence, a pattern consistent with observations in other gymnosperm genomes, such as *T. grandis* and *T. mairei*^7,24^.

Gene prediction was conducted using an integrative strategy that incorporated de novo prediction, homology-based alignment, and transcriptome evidence. A total of 36,488 and 36,360 high-confidence protein-coding genes were predicted for Hap1 and Hap2, respectively. BUSCO analysis based on conserved protein-coding genes revealed completeness scores of 95.0% for both haplotypes and 95.6% in combination (Fig. 1a, Supplementary Table 3 and Supplementary Fig. 5). Gene functional annotation was conducted using publicly available databases, including Nr, InterPro, Pfam, KEGG, GO, SwissProt, and eggNOG. In total, 34,332 and 34,136 genes from Hap1 and Hap2 were annotated in the Nr database, 32,976 and 32,801 in InterPro, 30,145 and 30,011 in eggNOG, 26,872 and 26,654 in Pfam, and 24,563 and 24,383 in SwissProt (Supplementary Table 6). Additionally, 25,971 (Hap1) and 25,811 (Hap2) genes were annotated with GO terms, and 9744 and 9736 genes were assigned KEGG pathway annotations. Overall, 96.13% of genes in Hap1 and 95.89% of genes in Hap2 were functionally annotated (Supplementary Table 6). These results exhibited that both haplotypes possessed high completeness and accuracy in both gene structure and function annotations.

### Phylogenetic analysis revealed lineage-specific adaptation in *Pseudotaxus chienii*

To investigate *P.* *chienii* evolutionary trajectory and adaptive features, we conducted phylogenetic analysis based on 421 single-copy orthologs, whereas a recent study by Wang et al. ^16^ based on a consensus genome assembly utilized 184 single-copy genes. The results revealed a clear topology: the three *Taxaceae* species (*P. chienii*, *T. mairei*, and *T. grandis*) formed a monophyletic group that diverged from *Metasequoia glyptostroboides* (*M. glyptostroboides*) approximately 169.01 Mya (Fig. 1c). Within the *Taxaceae* clade, *P. chienii* was resolved as sister to *T. mairei* (diverging at ~71.31 Mya), and this sister pair subsequently diverged from *T.* *grandis* at ~134.9 Mya. Our estimated divergence times differ from those reported by Wang et al. ^16^. This difference is expected and likely attributable to our distinct taxon sampling strategy and the substantially larger dataset of single-copy orthologs (421 vs. 184) utilized in our analysis. Furthermore, gene family clustering revealed 717 significantly expanded gene families in *P. chienii*, which were significantly enriched in transferase activity, polyketide biosynthesis proteins, and isoflavonoid biosynthesis (Fig. 1d and Supplementary Fig. 6). The 853 lineage-specific genes were enriched in proteasome, valine/leucine/isoleucine biosynthesis, and pyruvate metabolism. Furthermore, 3864 species-specific genes of *P. chienii* from a comparative analysis against three related conifers (*T. mairei*, *T. grandis*, and *M. glyptostroboides*) showed significant enrichment in structural constituent of ribosome, ubiquitin-protein transferase activity, glycolysis/gluconeogenesis, ribosome, and exosome (Supplementary Fig. 7).

### Conserved genomic architecture of paclitaxel biosynthetic loci in *Pseudotaxus chienii*

**Fig. 2:**
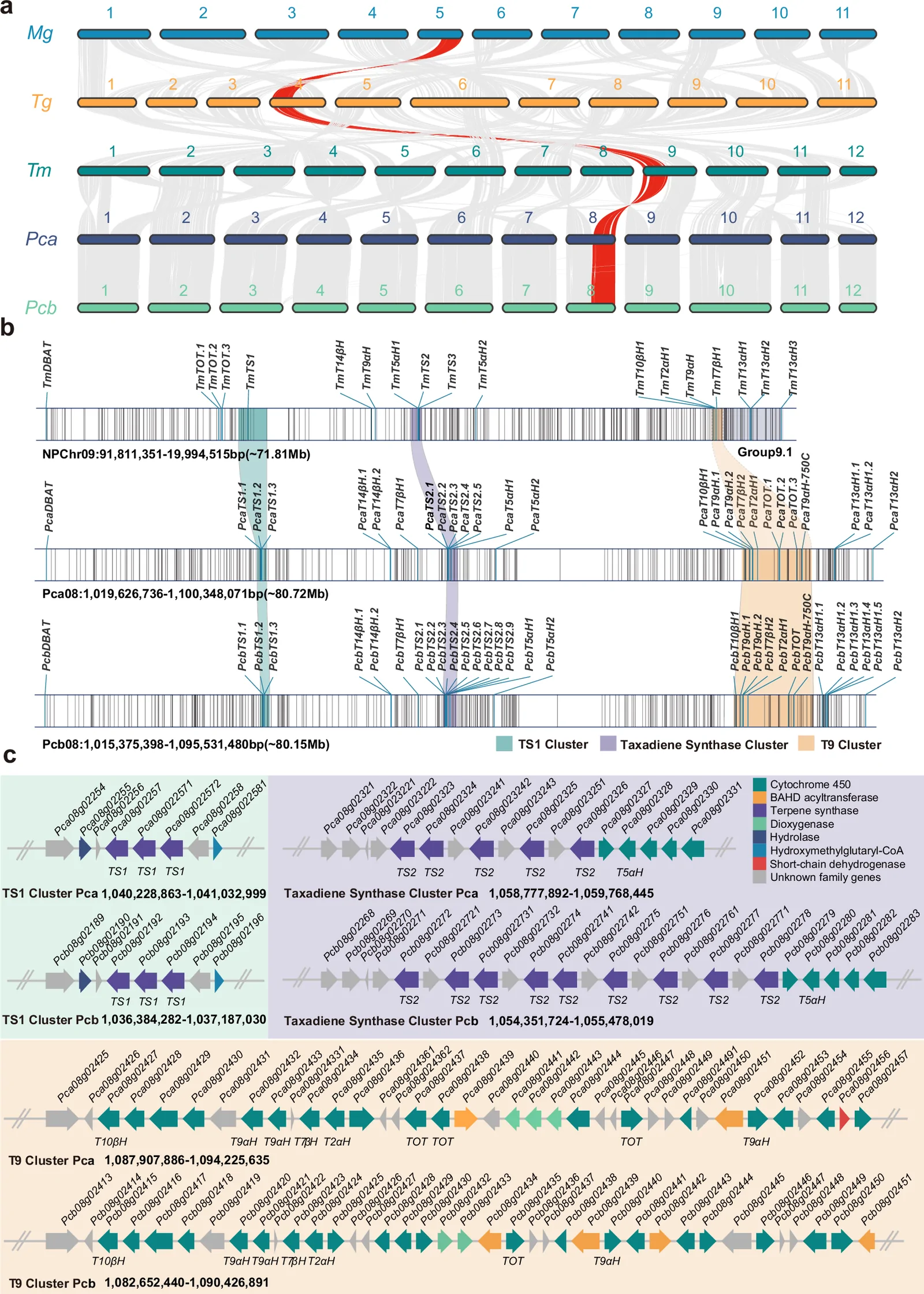
Genomic organization and structural features of Taxane Biosynthetic Archipelago (TBA) in *Pseudotaxus chienii*. **a** Macrosynteny among three *Taxaceae* species and the *Cupressaceae* outgroup *M. glyptostroboides* (Mg) highlights a conserved region containing three biosynthetic gene clusters (BGCs) and colinear upstream genes, including *TS*, *T5αH*, *T10βH* and *T13αH*. Tg, *T.grandis*; Tm, *T.mairei*; Pca/b, *P. chienii* hap1/hap2. **b** Colinear arrangement of paclitaxel biosynthetic genes within the TBA region. Genes involved in the paclitaxel biosynthetic pathway are shown in red. **c** Organization of three paclitaxel-related BGCs on *P. chienii* chromosome Pca08. Genes are color-coded by enzymatic function, including terpene synthases, cytochrome P450s and downstream tailoring enzymes involved in taxane modification. Source data are provided as a Source Data file.

Previous genomic investigations in *Taxus* have revealed that a cluster of key paclitaxel biosynthetic genes is located at the terminal region of chromosome 9^7,13^ (corresponding to chromosome 10^8^ or 12^9^ in certain assemblies). To determine whether this functional genomic architecture is evolutionarily conserved in *P. chienii*, we performed a genome-wide synteny analysis involving *T. mairei*, *T. grandis* and *M. glyptostroboides*. The global genomic landscape revealed a dynamic evolutionary history between *Taxus* and *Pseudotaxus*: while chromosome 2 exhibited syntenic stability with high linearity, most other chromosomes have undergone extensive inter-chromosomal rearrangements (Fig. 2a). Under this background of chromosomal reorganization, we identified a conserved ~80.7 Mb syntenic block on the terminal region of *P. chienii* chromosome 8 of hap1 (Pca08: 1,019,626,736–1,100,348,071) that exhibits collinearity with the homologous ~80.2 Mb region in the hap2 (Pcb08: 1,015,375,398–1,095,531,480) and a strong macrosynteny with *T. mairei* chromosome 9 (Chr9: 19,994,515–91,811,351) (Fig. 2a, Supplementary Fig. 8 and 9). In contrast, the corresponding regions in *T. grandis* and *M. glyptostroboides* were fragmented, preserving only scattered orthologs of paclitaxel biosynthetic genes (e.g., *T5αH* in both, *TS* and *T13αH* in *M. glyptostroboides*) (Fig. 2b). These results indicated that a large macro-syntenic block containing some of the core paclitaxel biosynthetic genes was specifically maintained in the genera *Taxus* and *Pseudotaxus* but was fragmented or absent in *T. grandis* and *M. glyptostroboides*.

### The taxane biosynthesis pathway in *Pseudotaxus chienii* is characterized by an archipelago architecture

Leveraging the ~80.7 Mb macro-synteny block conserved across both *P. chienii* haplotypes and positionally corresponding to on *T. mairei* chromosome 9 as an anchor, a genome-wide survey of both haplotypes revealed a rich landscape of secondary metabolism, identifying 21 candidate biosynthetic gene clusters (BGCs) in Pca and 25 in Pcb (Supplementary Fig. 10, Supplementary Data 1 and 2). Consistent with patterns previously documented in gymnosperms, these clusters are exceptionally large, with an average size of approximately 4.5 Mb and approximately 65% exceeding 2 Mb in length–a scale rarely seen in angiosperms^9,13,25^. Among them, a paclitaxel-enriched subregion on chromosome 8 contains three physically dispersed but functionally associated BGCs, designated TS1 Cluster (TS1C), Taxadiene Synthase Cluster (TSC), and T9 Cluster (T9C) within the conserved region of Pca08 (Fig. 2b). Although physically dispersed across the ~80.7 Mb block of Pca08, these three BGCs and several interspersed core paclitaxel biosynthetic genes located outside the predicted BGC boundaries are enriched in cytochrome P450s, BAHD acyltransferases, and terpene synthase enzymes, while maintaining collinearity between the two haplotypes and with the homologous region in *T. mairei*. The T9C exemplifies functional-genomic BGC definition: initially delineated at ~1.3 Mb in Pca via plantiSMASH based on gene proximity, its Pcb homolog spanned ~7.7 Mb. WGCNA analysis across an integrated dataset of 124 *P. chienii* transcriptomes confirmed co-expression among metabolism-related genes across the entire 7.7 Mb Pcb region, a pattern conserved in the corresponding Pca region, thereby justifying the extension of the Pca T9C boundary to ~6.3 Mb beyond the proximity-based limit (Supplementary Fig. 11 and Supplementary Data 3).

To quantitatively validate the functional integration of this dispersed genomic archipelago, we combined chromatin architecture profiles derived from Hi-C analysis (using the Pca haplotype as a representative model) with Pearson correlation analyses of a comprehensive 124-sample transcriptomic dataset. Hi-C profiles revealed that the entire TBA region is embedded within the transcriptionally active A compartment^26,27^, providing a globally favorable environment for gene expression. The three gene clusters are spatially segregated into distinct Topologically Associating Domains (TADs)^28,29^, which further subdivide into localized sub-TADs (Supplementary Fig. 12a). This hierarchical division closely aligns with functional boundaries; for example, the TAD encompassing TS1C exclusively harbors core metabolic genes, effectively demarcating the functional domain by excluding a non-metabolic gene at its left border. Although separated into independent TADs, metabolic genes from the three clusters and these dispersed core enzymes form a highly coordinated co-expression network, with the majority of gene pairs exhibiting Pearson correlation coefficients (r) ≥0.9 (Supplementary Fig. 12b). Collectively, these findings indicate that the functional integrity of the TBA may be supported by regulatory coordination within an active chromatin environment, rather than strictly by linear physical proximity. This archipelago-like organization, characterized by both functionally linked but spatially separated clusters and several dispersed core enzymes, contrasts with the more compact BGCs typical of microbes and angiosperms^30,31^ (Supplementary Fig. 13).

We therefore propose the term Taxane Biosynthetic Archipelago to describe this distinctive arrangement of paclitaxel biosynthetic genes in *P. chienii*, with partially conserved syntenic elements in the sister genus *Taxus*. The TBA exhibits structural plasticity in integrating conserved core modules with lineage-specific accessory elements, and is characterized by extensive serves as a dynamic region for asymmetric evolution. Each lineage has undergone extensive gene amplifications and rearrangements. For instance, the T9C (collinear with the previously defined Group 9.1 in *T. mairei*^*7*^) highlights this plasticity (Fig. 2b), with the *TOT* genes varying from three copies in Pca to a single copy in Pcb, and appearing translocated near the TS1C in the *T. mairei* (Fig. 2b, c). Additionally, the Pcb haplotype may have an enhanced potential for terpene scaffold generation through the presence of nine copies of the *TS2*, an independent clade characterized by significant gene expansion and jasmonate-dependent regulatory specialization based on the classification established in *T. mairei*^*7*^, compared to only five in Pca (Fig. 2c). To uncover the regulatory basis of this *TS2* expansion, we analyzed promoter cis-elements across both haplotypes of *P. chienii* and *T. mairei*. A clear regulatory divergence was observed: *TS2* promoters are highly enriched in Methyl jasmonate (MeJA)-responsive elements (e.g., CGTCA-motifs, TGACG-motifs, and MYC binding sites)^32,33^ (Supplementary Fig. 14), which are largely absent in TS1. This pronounced structural divergence suggests a regulatory specialization: while TS1 likely supports baseline expression, the expanded TS2 genes are transcriptionally primed for stress-induced responses.

### Phylogenomic insights into the origin and diversification of the paclitaxel pathway

To investigate whether *P. chienii* retains the complete set of enzymes required for paclitaxel biosynthesis, we performed gene distribution analysis of 28 key genes involved in the paclitaxel biosynthetic pathway across representative land plants (Supplementary Fig. 15 and 16). *P. chienii* harbors copies of all currently known paclitaxel biosynthetic genes, indicating the retention of a largely complete genomic complement associated with paclitaxel biosynthesis. The upstream precursor biosynthesis genes geranylgeranyl diphosphate synthases (*GGPPS*), *FoTO1*, and the phenylpropanoid pathway gene phenylalanoyl-CoA ligase (*PCL*) are conserved across all surveyed land plant lineages from bryophytes to angiosperms, consistent with their inheritance from a common ancestor of embryophytes.

In contrast, the core taxane backbone-forming terpene synthases *TS1* and *TS2* are absent in bryophytes, *Gnetales* and angiosperms, but present in *Pinaceae*, most *Cupressaceae*, and *Taxaceae*. Despite their presence in these broader conifer lineages,*TS* orthologs were not detected in *Gnetum montanum*, *Cupressus gigantea*, and *T. grandis*. Specifically, the absence of these core genes in the basal *Taxaceae* species *T. grandis*, coupled with its highly fragmented syntenic region (Fig. 2a and Supplementary Fig. 8), is more consistent with secondary loss events following lineage divergence, rather than incomplete lineage sorting. This phylogenetic distribution of *TS* is potentially driven by gene duplication and subsequent functional specialization within the terpene synthase family, as demonstrated by the functional divergence of *TS* orthologs in *Taxus wallichiana* and their absence in non-Cupressales species^8^.

A second suite of downstream pathway genes involved various oxygenases, acyltransferases, and side-chain assembly enzyme (*T5αH1*, *T13αH1*, *T9αH-750C*, *T7dA*, *T9dA*, *TAX19*, *DBTNBT* and *PAM*) are present in most conifer lineages (*Pinaceae*, *Taxaceae*, and *Cupressaceae*) and absent from *Gnetales*, bryophytes, and angiosperms. The final set of pathway genes comprising additional likely functional enzymes (e.g., *T10βH1*, *T2αH*, *T7βH1*, *TOT*, *T9αH*, *T7AT*, taxane 2α-*O-*benzoyltransferase (*TBT1*), *BAPT*) are exclusively detected in the two sequenced *Taxaceae* genomes (*T. mairei* and *P. chienii*), with 1–3 copies of each gene and zero copies in all other species surveyed.

This hierarchical distribution of these genes, spanning from ubiquitous precursors, through conifer-enriched modifications, to a suite of oxygenation, acylation and side-chain assembly enzymes, supports a step-wise evolutionary assembly of the genomic pathway complement. This indicates that the genomic architecture associated with paclitaxel biosynthesis may have been assembled in the most recent common ancestor of *Taxus* and *Pseudotaxus*. Furthermore, orthologs shared between *P. chienii* and *T. mairei* exhibited low Ka/Ks ratios (mean Ka/Ks ≈ 0.31; Supplementary Data 4), indicating sustained purifying selection on their coding sequences^34^.

### Transcriptomic and geographical and temporal targeted metabolite quantification reveal a restricted paclitaxel accumulation

**Fig. 3:**
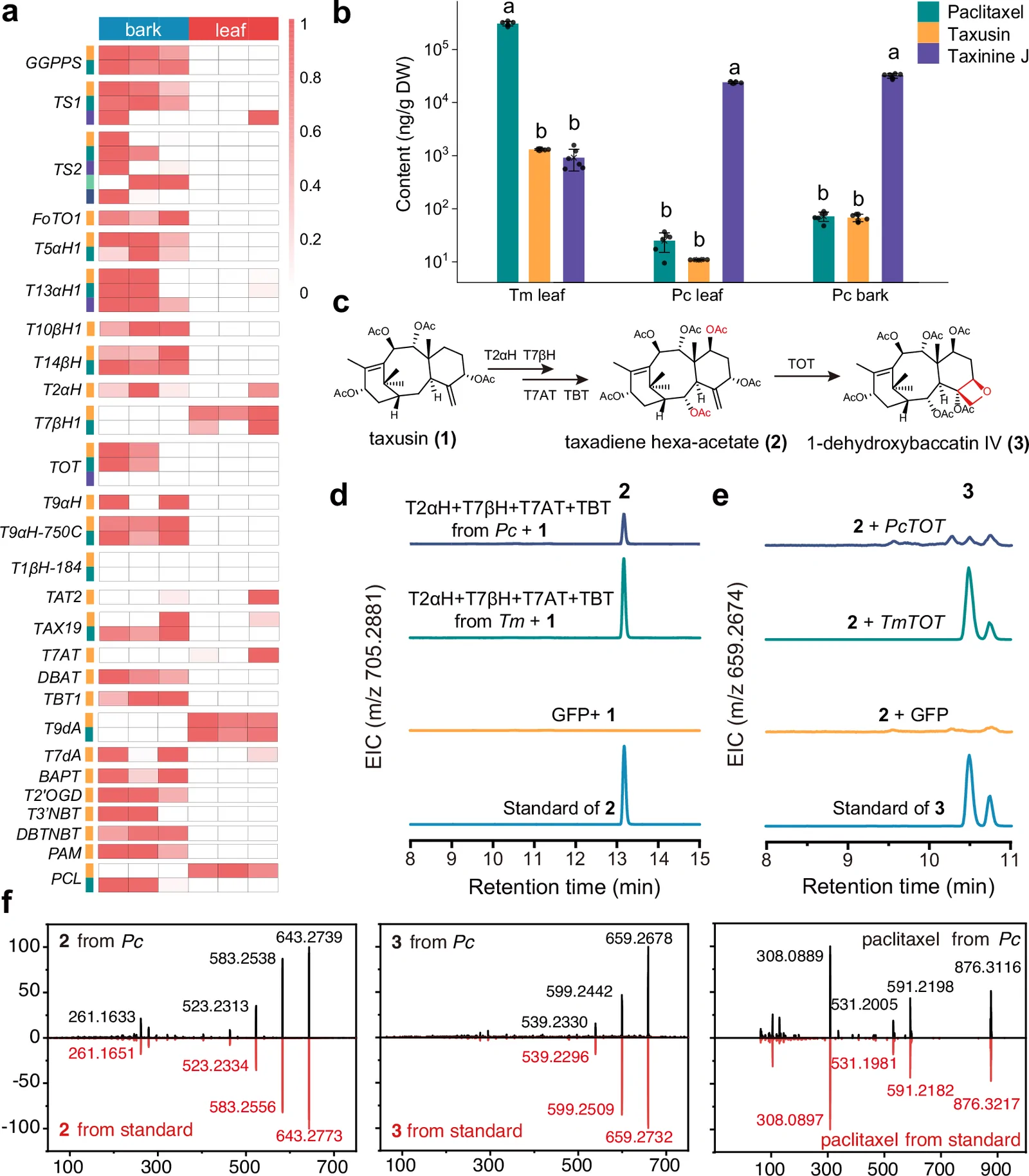
Identification of the paclitaxel biosynthetic pathway in *Pseudotaxus chienii*. **a** Heatmaps display the expression levels of corresponding biosynthetic genes in *P. chienii* leaf and *P. chienii* bark tissues. Different colors represent different gene copies in *P. chienii* hap1. **b** Targeted LC-MS profiles of key taxane metabolites in Pc leaf, Pc bark, and *T. mairei* leaf (Tm leaf). Sample sizes for Pc leaf, Pc bark, and Tm leaf were n = 6, 6, and 6 for paclitaxel; *n* = 5, 6, and 6 for taxusin; and n = 4, 6, and 6 for taxinine J, respectively. Statistical comparisons were performed on the original concentration values using one-way ANOVA followed by Fisher’s least significant difference (LSD) test in OriginPro 2026 SR1 (V 10.3.0.197), with *P* < 0.05 considered statistically significant. Different lowercase letters indicate a significant difference. Error bars represent means ± SD of independent biological replicates. Note: The Y-axis is plotted using a Log10 scale to accommodate the magnitude of concentration differences between species, which visually compresses the error bars representing biological variability. **c** Schemati**c** of T2αH, T7βH, T7AT, and TBT convert taxusin (**1**) to taxadiene hexa-acetate (**2**), and the schematic of TOT convert taxadiene hexa-acetate (**2**) to 1-dehydroxybaccatin IV (**3**). **d**,** e** EICs of expected products when the T2αH, T7βH, T7AT, TBT (**d**) and TOT (**e**) from *P. chienii* or *T. mairei* is expressed in *N. benthamiana*. **f** MS/MS spectra of taxadiene hexa-acetate (**2**), 1-dehydroxybaccatin IV (**3**) and paclitaxel detected in an extract of the *P. chienii* (black) sample SQS001 (2024) and its standard (red). Source data are provided as a Source Data file.

To elucidate the expression pattern of paclitaxel biosynthetic genes, we performed transcriptome profiling of *P. chienii* tissues (Fig. 3a). Upstream enzymes (*GGPPS*, *TS1/2*, *FoTO1*) and the majority of pathway genes (including most hydroxylases and acyltransferases), showed higher expression in bark compared to leaves (Fig. 3a). Conversely, select genes such as *T9dA* and *T7βH1* exhibited leaf-preferential expression, suggesting that this may be a result of functional divergence in branch pathways or non-canonical taxane metabolism (Fig. 3a).

To corroborate the transcriptomic findings and further investigate the controversy surrounding the presence of paclitaxel in *P. chienii*, preliminary targeted quantification of metabolites from *P. chienii* leaf (SQS001, April 2024) was performed using a SCIEX 5500 QTRAP LC-MS/MS system and a Thermo Scientific TSQ 9000 triple-quadrupole mass spectrometer coupled with a Trace 1310 gas chromatograph for GC-MS/MS detection. A total of over 41 taxane metabolites were identified across the two detection platforms (Supplementary Fig. 17a, b), including paclitaxel and taxusin, a key intermediate in the paclitaxel biosynthetic pathway^12^. Among these, taxinine J was the predominant constituent, a potential anticancer agent^35,36^, accompanied by substantial amounts of several structurally related taxinine derivatives. Given that paclitaxel and taxusin were also detected within this complex profile, and noting the absence of the downstream intermediate baccatin Ⅲ, we selected these four representative metabolites for targeted LC-MS analysis in both *P. chienii* and *T. mairei* using SCIEX 5500 LC-MS/MS platform. Importantly, baccatin Ⅲ remained below the limit of detection in both leaf and bark tissues of this initial *P. chienii* individual, so only three quantifiable taxanes are included in the comparative analysis (Fig. 3b). All three compounds could be detected in *P. chienii*, with significantly higher levels in bark than leaves (Fig. 3b). Additionally, *T. mairei* leaves accumulated substantially higher levels of paclitaxel and taxusin than any *P. chienii* tissues, whereas taxinine J accumulated to higher levels in *P. chienii* tissues than each *T.mairei* tissues. Within this initially sampled *P. chienii* individual, both paclitaxel and taxusin remained at trace amount in leaves, whereas taxinine J accumulated to substantially higher levels (Fig. 3b).

Given the trace accumulation of paclitaxel in the initial sample, we aimed to systematically determine whether this pattern was a consistent pattern across different populations of the species or a phenomenon limited to a specific region. We performed a comprehensive spatiotemporally targeted taxoid analysis on 2 distinct sampling batches from 6 independent *P. chienii* trees. The samples were taken from two geographically isolated habitats with distinct latitudes and longitudes: Sanqingshan (samples SQS001, SQS006) and Jinggangshan (samples GLM51 - GLM54) (Supplementary Fig. 18a, b). To assess temporal variation, we collected leaf and bark samples at two time points: spring 2024 (April) and winter 2026 (January), yielding a total of 18 samples (representing available tissues across seasons). All 18 spatiotemporal samples were analyzed in parallel using a high-sensitivity SCIEX Triple Quad 7500 LC-MS/MS system, while 2024 archived SQS001 samples were previously cross-validated on a SCIEX Triple Quad 5500 LC-MS/MS platform.

Across all tissues, sampling sites and time points detected, taxinine J exhibited the highest abundance among the four targeted taxanes (Supplementary Fig. 18a, b). In marked contrast, baccatin Ⅲ was undetectable in all assayed samples. Trace levels of paclitaxel were exclusively found in leaves of a single *P. chienii* individual (SQS001) collected from Sanqingshan in April, 2024. Interestingly, in the Jinggangshan populations, although taxinine J maintained the highest abundance, taxusin also accumulated to a high level in selective samples, with its concentration peaking at approximately half that of taxinine J. Furthermore, following MeJA elicitation, trace paclitaxel was detected in leaves from only one out of three independent *P. chienii* branches after 48 h of treatment, whereas no paclitaxel was captured in the 96 h treatment group (Supplementary Fig. 18a), and no paclitaxel was detected any natural samples collected in 2026.

### Functional characterization identifies a severe downstream bottleneck in paclitaxel biosynthesis

To uncover the biochemical basis of enzymatic function underlying the evident metabolic bottleneck and the universal absence of baccatin III in the examined samples, we systematically assessed the enzymatic capability of the paclitaxel biosynthesis pathway by transient co-expression in a heterologous *Nicotiana benthamiana* system. Guided by comparative sequence analysis, we prioritized candidate genes exhibiting lower sequence identity between *P. chienii* and *T. mairei* for functional verification (Supplementary Data 4). Specifically, we cloned *P. chienii* genes including Taxane 2α-hydroxylase (*T*2α*OH*), Taxane 7β-hydroxylase (*T7βOH*), taxane C-7β-*O-*acyltransferase (*T7AT*), *TBT*, and *TOT*, and transiently co-expressed them in *N. benthamiana*, with corresponding *T. mairei* genes as controls (Fig. 3c). The *P. chienii* enzymes T2αH, T7βH, T7AT, and TBT efficiently converted taxusin (**1**) to taxadiene hexa-acetate (**2**), exhibiting activities comparable to *T. mairei* orthologs (Fig. 3d, f). These heterologous expression results clearly indicate that the upstream and mid-stream steps of the paclitaxel pathway retain catalytic competence in *N. benthamiana*, whereas an obvious bottleneck arises immediately at the subsequent step. *P. chienii* TOT converted taxadiene hexa-acetate (**2**) to 1-dehydroxybaccatin IV (**3**) at markedly lower efficiency than *T. mairei* TOT (Fig. 3e, f). To preliminarily elucidate the reasons for the attenuated function of PcTOT compared to TmTOT, structural modeling and molecular docking analyses were first performed. The results revealed a slightly more relaxed catalytic conformation in PcTOT (2.7 Å/149.2°) compared to the highly optimized geometric parameters observed in TmTOT (2.4 Å/157.7°) (Supplementary Fig. 19a), despite their overall structural similarity with a root-mean-square deviation (RMSD) of 1.67 Å across 2,766 backbone atoms (Supplementary Fig. 19b). Intriguingly, molecular dynamics simulations revealed distinct, region-specific flexibility patterns across these structural regions. Specifically, PcTOT exhibited a pronounced conformational mobility within the R1 segment, yet displayed a distinctly repressed flexibility, evidenced by lower calculated residue-level root-mean-square fluctuation (RMSF) within R2 and R3 regions (Supplementary Fig. 19c**)**. Based on CAVER analysis, these differentially rigidified regions were spatially mapped onto the residues lining the substrate access channels leading to the buried catalytic pocket (Supplementary Fig. 19d**)**. Taken together, these complementary static and dynamic analyses suggest that the altered flexibility and subsequent channel gating dynamics likely contribute to the divergent catalytic behaviors of the two enzymes. Moreover, despite genomic retention and high sequence conservation with *Taxus* orthologs, heterologous expression of six core downstream candidates, including *T9ox* and *BAPT*, in *N. benthamiana* failed to yield detectable pathway products, in sharp contrast to active *T. mairei* counterparts (Supplementary Fig. 20**)**.

## Discussion

The 15.43 Gb genome assembly of *P. chienii*, with its high-quality annotation, provided a valuable reference for the *Taxaceae* family and gymnosperms^7–9,13,37^. We compared its contig N50, BUSCO completeness of protein model and repeat content with representative gymnosperms and a recently released *P. chienii* genome assembly, confirming the high reliability of our genomic dataset (Supplementary Data 5)^7,13,16,24,37–41^. Although consensus assembly may have better linear contiguity, our haplotype-resolved assembly more effectively resolves the structural complexity of this highly heterozygous genome with over 88% repetitive sequences. Our protein annotation achieves a 95% BUSCO completeness, one of the highest among reported gymnosperms, and this high-resolution assembly facilitates the accurate reconstruction of intact metabolic pathways. The present study showed that while both *Pseudotaxus* and *Taxus* underwent significant LTR-retrotransposon expansion, their genomic structures evolved differently. The major LTR expansion in *P. chienii* (~ 21.59 MYA) occurred earlier than in *T. mairei*. This expansion period aligns closely with major Early Miocene climatic shifts, most notably the strengthening of the Asian monsoon^42^. Though current substitution rates based on molecular dating cannot confirm direct causality, severe environmental fluctuations likely triggered demographic bottlenecks^43^. Such population declines could subsequently reduce the efficacy of purifying selection, permitting the extensive retention of repetitive elements^44^. The larger genome of *P. chienii* was primarily attributed to the inefficient removal of ancient repetitive elements resulting from a lack of unequal recombination, rather than more frequent transposition^45,46^. In many plants, DNA loss (through unequal recombination) balanced genome growth; however, in *P. chienii*, this imbalance between DNA gain and DNA loss has led to the long-term accumulation of repetitive sequences. This mechanism may explain how this “living fossil” maintained such an expansive genome despite a relatively ancient burst of transposon activity. As gymnosperm genomes undergo extensive expansion through the relentless proliferation of repetitive elements, the maintenance of intact biosynthetic gene clusters in linear arrays becomes increasingly difficult^24,45^.

To address this challenge, we introduced the TBA, which illustrates a striking evolutionary adaptation: pathway coordination within these large phylogenetic lineages operates independently of physical gene proximity. Defined by functionally linked yet spatially dispersed biosynthetic gene clusters, the TBA represents a departure from the canonical model of compact BGCs^30^, raising a key question of how functional coordination is maintained across large genomic regions. Traditional models generally invoke direct chromatin looping to account for long-range co-expression, whereas our findings reveal a distinct spatial regulatory paradigm in expanded gymnosperm genomes. Although spatially segregated into distinct TADs, the three TBA clusters maintain robust regulatory coordination within a shared active chromatin environment despite their linear separation. Instead, this coordinated expression may be supported by their localization within the transcriptionally active A compartment, coupled with unified trans-regulatory signals. This insight refines the evolutionary understanding of specialized metabolism. In large expanded genomes, the integrity of biosynthetic pathways may not require linear contiguity and may instead be maintained within broader chromatin environments. Rather, functional modules may remain topologically separated while being synchronously modulated by common global regulatory signals^47^. The TBA model expanded the concept of biosynthetic gene organization, offering a complementary approach to identify coordinated metabolic pathways in large or complex genomes where functional genes were physically dispersed, which may evade detection by conventional gene cluster search tools reliant on physical proximity. This dispersed organization may represent an adaptive feature in expansive genomes, facilitating pathway evolution amid repetitive element accumulation^46^.

The haplotype-resolved assembly revealed significant genomic differences between the two haplotypes, particularly in copy number variation. For example, the Pcb haplotype contains nearly twice as many copies of the *TS2* gene as Pca. While determining the direct metabolic consequence of this specific allelic expansion is technically impractical within the blended diploid metabolome, our promoter analyses provide robust alternative evidence for its functional role. These analyses provide support for an adaptive gene dosage strategy^48^: TS2 gene expansion may enable *P. chienii* to enhance JA-responsive chemical defense upon stress, effectively balancing defensive capacity and metabolic expenditure. Furthermore, such underlying genetic variation may interact with environmental changes to shape distinct chemotypes, underscoring the need to account for G × E interactions in exploring metabolic diversification^49^. Given the endangered status of *P. chienii*, these findings carry important implications for the development of more effective conservation strategies to protect its distinctive taxane biosynthetic capability. Rather than focusing exclusively on increasing population sizes, in situ conservation efforts should prioritize the protection of heterogeneous microhabitats so as to preserve G × E interactions that may contribute to chemical diversity. At the same time, ex situ germplasm collections can utilize the haplotype-resolved markers generated in this study to capture and safeguard the full range of chemotypes along with the species’ underlying genetic architecture.

Resolving the historical debate surrounding paclitaxel biosynthesis in *P.* *chienii* requires addressing not only contradictory metabolic evidence, but also divergent genomic interpretations reported in recent studies. For instance, Wang et al. ^16^ proposed that the pathway terminates prior to 10-deacetylbaccatin III, a conclusion that may partly reflect the absence of several corresponding pathway homologs from their consensus chromosome-scale genome assemblies. In contrast, our haplotype-resolved genome demonstrates that a largely complete genetic architecture underlying paclitaxel biosynthetic pathway, including the core TBA and other dispersed essential genes, remains structurally intact in *P. chienii*. These results further highlighted the critical importance of haplotype-aware genome assemblies for accurately reconstructing complex specialized metabolic pathways in highly heterozygous plant genomes. However, the observed discrepancies between this genomic retention and the restricted biochemical output suggest a dynamic evolutionary trajectory rather than a contradiction. While our phylogenomic analysis indicates that the genomic complement associated with the pathway was step-wise assembled and may have achieved functional integrity in the common ancestor of *Taxus* and *Pseudotaxus*, the retention of this ancient genomic architecture does not equate to modern functional execution. Following its divergence from *Taxus*, the terminal pathway in the *P. chienii* lineage appears to have undergone significant evolutionary functional degradation. The paclitaxel content in *P. chienii* leaves is over four orders of magnitude lower than that typically observed in *T. mairei*. Crucially, our findings reveal that the capacity for paclitaxel biosynthesis outside the *Taxus* genus cannot be simply reduced to a binary question of gene presence or absence. Rather, it represents a clear case of evolutionary functional degeneration. Although the ancestral archipelago architecture has been faithfully retained and the complete set of orthologous pathway genes displays low Ka/Ks ratios indicative of sustained purifying selection^34^ and has been robustly preserved to cope with extensive genome expansion, such structural conservation does not necessarily guarantee full functional integrity. Indeed, paclitaxel could only be detected in rare instances: specifically, in a single natural sample (SQS001) collected in April 2024 (independently detected on two LC-MS/MS platforms; the SCIEX Triple Quad 5500 LC-MS/MS spectrum is shown in Fig. 3f, and the SCIEX Triple Quad 7500 LC-MS/MS result is shown in Supplementary Fig. 18), together with only one positive case observed among all independently MeJA-induced branches in 2026 (Supplementary Fig. 18). These rare detections do not establish residual paclitaxel biosynthetic activity, although such activity cannot be completely excluded. Moreover, given the low abundance and limited reproducibility of the MeJA-associated signal, trace contamination arising during sample handling, extraction, or instrumental analysis may represent an alternative explanation for this isolated MeJA-associated detection^50,51^. Independently, the markedly reduced catalytic activity of PcTOT and the absence of detectable activity for several downstream enzymes under the tested conditions support severe functional attenuation of the terminal pathway. Taken together, the combined biochemical and metabolomic evidence suggests that the absence of functional capacity for complete paclitaxel biosynthesis is a plausible interpretation, while weak residual activity under specific native conditions remains an unresolved possibility. The highly limited and inconsistent detection of paclitaxel may help explain the conflicting reports regarding its occurrence in *P. chienii* across previous studies^14–16^. Resolving these alternatives and evaluating the possible contribution of G × E interactions to the observed variation in paclitaxel detection will require broader population-level and spatiotemporal sampling across multiple genotypes and environmental conditions, more extensive biological replication, stringent analytical verification, and further biochemical and *in planta* functional investigations.

To systematically investigate whether this functional deficiency extends across the downstream pathway, we characterized two core late-stage functional modules. The first module contains key oxygenases involved in skeleton modification, including T9ox (65.3% sequence identity to the *Taxus* ortholog), T9αH-750C (68.7% identity), and taxane 1β-hydroxylase (T1βH-184) (68.2% identity). The second module includes terminal acyltransferases responsible for side-chain formation and attachment, namely baccatin III:3-amino-3-phenylpropanoyl transferase (BAPT) (67.8% identity), taxoid-3′*-N-*benzoyltransferase (T3’NBT) (73.0% identity), and 3’*-N-*debenzoyl-2’-deoxypaclitaxel*-N-*benzoyl transferase (DBTNBT) (71.9% identity). Despite genomic retention and considerable sequence conservation, none of the genes from either downstream module produced detectable pathway products in *N. benthamiana*, whereas the functional competence of the corresponding *T. mairei* orthologs remained intact (Supplementary Fig. 20).

Together with the significantly lower product formation observed for PcTOT, the absence of detectable activity across these downstream functional modules in the heterologous system indicated profound functional attenuation of the terminal paclitaxel biosynthetic pathway. Although we cannot completely exclude the possibility that these enzymes may retain weak basal activities in the presence of *P. chienii* specific co-factors or native cytochrome P450 reductases that are absent from the heterologous system, the overall heterologous *in planta* enzymatic profile, which is characterized by catalytic activities near or below the detection threshold, is consistent with our endogenous targeted metabolite analyses.

Our findings indicate that although *P. chienii* retains a largely complete genetic architecture for paclitaxel biosynthesis, this potential is not fully realized at the level of end-product accumulation. Instead, metabolic output appears to be preferentially channeled toward alternative taxane branches, particularly taxinine J. This possible metabolic rewiring is suggested by the marked accumulation of taxinine J, along with discernible levels of taxusin in specific spatiotemporal samples, and the consistent absence of downstream intermediates like baccatin Ⅲ across sampled wild populations. Rather than conclusively indicating either complete evolutionary loss or retained residual capacity, this specific taxane accumulation profile provides a biochemical basis for severe functional attenuation of the paclitaxel biosynthesis pathway. Therefore, *P. chienii* may have lost the functional capacity for complete paclitaxel biosynthesis, although a residual, trace-level capacity under specific native conditions, possibly influenced by G × E interactions, cannot be excluded and remains to be verified^52^. This phenomenon is likely rooted in the evolutionary repurposing and progressive functional degeneration of ancestral metabolic architectures^53^.

Ultimately, the haplotype-resolved genome of the endangered *P. chienii* provides a valuable new framework for understanding how complex specialized metabolism evolves in massively expanded plant genomes. By introducing the TBA, we extend the classical concept of metabolic gene clusters, showing that within such repeat-rich gymnosperm genomes, coordinated expression of pathway-associated genes can be maintained despite their physical dispersion and may be supported by shared transcriptionally permissive chromatin environments and trans-acting regulatory signals. Importantly, the combined use of structural genomics, spatiotemporal metabolite profiling, and heterologous *in planta* functional assays helps clarify the long-standing debate over whether paclitaxel can be produced outside the *Taxus* genus. Our results point to a more subtle pattern of metabolic evolution: although the ancestral genomic architecture has been largely retained amid extreme genome expansion, pronounced functional degeneration at the later catalytic steps has constrained downstream pathway progression and is accompanied by the preferential accumulation of alternative taxane products such as taxinine J, its various structurally related derivatives (Supplementary Fig. 17), and discernible levels of taxusin across different populations. These findings thus refine our understanding of taxane diversification and offer a critical genomic foundation for both the conservation of *Taxaceae* and the engineered production of high-value taxanes.

## Methods

### Plant materials and sample collection

Fresh leaves and bark tissues were collected from two mature wild individual of *Pseudotaxus chienii* (accession SQS001 (male) and SQS006) located in Sanqingshan National Park (28.90087° N, 118.08942° E), Jiangxi Province, China. The sampling site was at an elevation of approximately 1,296 m above sea level. Additional fresh leaf and bark samples were collected from four mature wild individuals (accessions GLM51 - GLM54) located in Jinggangshan (26.510254° N, 114.161766° E), Jiangxi Province, China, at an elevation of approximately 1,292 m. Samples from these individuals were collected in April 2024 and January 2026. Collected tissues were immediately snap-frozen in liquid nitrogen and stored at – 80 °C for subsequent analyses.

### DNA and RNA extraction

High-quality genomic DNA was extracted from young leaves using a modified CTAB method. The DNA was treated with RNase A to remove RNA contaminants and further purified using AMPure XP beads (Beckman Colter, USA). DNA quantity and quality were assessed with a NanoDrop spectrophotometer, Qubit 4.0 fluorometer, agarose gel electrophoresis, and pulsed-field gel electrophoresis. Total RNA was extracted from leaf and bark tissues using the TRIzol reagent (Invitrogen, USA) and treated with RNase-free DNase I (New England Biolabs, USA) to eliminate residual genomic DNA. RNA integrity was assessed using an Agilent 2100 Bioanalyzer, and only samples with an RIN value greater than 9.0 were used for sequencing.

### Library construction and sequencing

For PacBio HiFi sequencing, following an evaluation of the isolated DNA’s quality, a 20 kb library was prepared with the SMRTbell Express Template Prep Kit 2.0 from Pacific Biosciences (Menlo Park, CA, USA). After passing quality checks, the resulting SMRTbell library was sequenced on the PacBio Revio system with one 8 M SMRT Cell (Pacific Biosciences, Menlo Park, CA, USA), yielding 677.65 Gb of HiFi reads ( ~ 47.25× coverage). Short-read genomic libraries were generated and sequenced on the MGI T7 platform (150 bp paired-end reads), producing 1,530.76 Gb of data ( ~ 99.2× coverage). For Hi-C analysis, chromatin from formaldehyde-fixed leaf nuclei was digested using the DpnII enzyme, proximity-ligated, sheared to 300–500 bp fragments, and sequenced on the MGI T7 platform to generate 2336.39 Gb of reads ( ~ 151.42 × coverage). Transcriptomic libraries for RNA-seq were prepared using the VAHTS Universal V10 RNA-seq Library Prep Kit for Illumina (vazyme, China) and sequenced on the MGI T7 platform. Iso-Seq libraries were also prepared using the SMRTbell Express Template Prep Kit 2.0 and sequenced on the PacBio Revio platform, resulting in 54.42 Gb of full-length transcriptome data. All library construction and sequencing steps adhered strictly to manufacturer protocols and quality control standards.

### Genome size estimation

Genome size was estimated through flow cytometry (CytoFLEX, Beckman Colter, USA) using *Taxus mairei* as an internal reference. Three biological replicates were analyzed following propidium iodide staining. Additionally, genome size, heterozygosity, and repetitive element content were determined using K-mer (21-mer) analysis performed with GCE v1.0.2^54^ on all clean MGI short-read data, evaluating K-mer depth distributions and peak features.

### Genome assembly and scaffolding

HiFi reads (677.65 Gb; ~47.25× genome coverage) were assembled de novo using hifiasm v0.16.1^55^, with Hi-C data incorporated for haplotype phasing and chromosome-scale scaffolding. Hi-C data were processed through the Juicer v1.6^56^ pipeline to generate contact maps, and contigs were anchored and oriented onto pseudochromosomes using 3D-DNA v180922^57^. Manual correction of misassemblies and scaffolding errors was conducted using Juicebox Assembly Tools v1.11.08^58^. Additionally, the final Hi-C interaction heatmaps were generated and visualized using HapHiC v1.0.7^59^. Gaps between contigs were filled using the gap-based genome filling (GBGF) method to improve assembly continuity^60^. Consensus sequence polishing was performed using merfin v1.0^61^ based on MGI short-read data. Assembly quality was assessed using BUSCO v5.0.0^62^ (Embryophyta_odb10, 1,614 single-copy orthologs) to evaluate completeness, as well as Merqury v1.3^63^ to calculate base-level accuracy and genome integrity, ensuring high confidence in the assembly quality.

### Repeat annotation and gene prediction

Repetitive sequences were identified using RepeatModeler v2.0.5^64^ to construct a species-specific transposable element (TE) library, which was combined with the Repbase database and used by RepeatMasker v4.1.5^65^ for comprehensive repeat detection and masking. Gene structure prediction employed a multi-tiered strategy: (1) de novo gene prediction using Augustus v3.5.0 and BRAKER v3.0.8^66^, integrating ab initio models and RNA-seq evidence; (2) homology-based prediction via GeMoMa v1.9^67^ using protein sequences from *Physcomitrella patens*^60^, *Amborella trichopoda var. SantaCruz_75* HAP1 v2.1^68^, *Oryza sativa* v7.0^68^, and *Arabidopsis thaliana* araport11^68^ as references; (3) transcript-based prediction involving Iso-Seq data processing followed by short-read RNA-seq assembly, both optimized with PASA v2.4.1^69^. Predictions were integrated using EVidenceModeler v1.1.1^69^, further refined by the PASA update module, and manually inspected and corrected using IGV v2.14.0^70^ to produce a high-confidence gene annotation set.

### Functional annotation

Functional annotation of protein-coding genes employed DIAMOND v2.0.7^71^ against NR^72^, Swiss-Prot^73^, and eggNOG^74^ databases (E-value ≤ 1e⁻⁵, alignment coverage ≥50%), complemented by domain searches using InterProScan v5.50-84.0^75^ (Pfam, SMART, CDD, TIGRFAMs, ProSite). Pfam-A^76^ domain confirmation was conducted using HMMER v3.4^77^. Pathway annotations were generated via KEGG Automated Annotation Server (KAAS)^78^ and eggNOG, with GO annotations derived from InterProScan and eggNOG ortholog groups. All functional annotations were filtered to retain those with domain coverage ≥50% and KEGG orthology confidence ≥0.8, adhering to established genome annotation protocols.

### Phylogenomics and genome evolution

OrthoFinder v2.5.2^79^, combined with DIAMOND, was used to identify orthologous gene families across *P. chienii* and ten representative plant genomes, including *Physcomitrella patens*, *Amborella trichopoda*, *Arabidopsis thaliana*, *Croomia sessilifolia*, *Cycas panzhihuaensis*, *Pinus tabuliformis*, *Gnetum montanum*, *Metasequoia glyptostroboides*, *Torreya grandis*, and *T. mairei*. Among them, some gene models of *T. mairei* genome annotation were corrected. A total of 421 single-copy orthologous gene families were identified and aligned using MUSCLE v5^80^. Phylogenetic trees were constructed using RAxML v8.2.12^81^ with the PROTGAMMAJTT model and 1,000 bootstrap replicates. Specifically, divergence times were estimated using MCMCTree (PAML v4.9j)^82^ under a relaxed molecular clock model. To calibrate the absolute evolutionary timeline, fossil calibration points obtained from the TimeTree database^83^ were applied to specific nodes. Whole-genome duplication (WGD) events were detected using WGDI v0.5.1^84^ through collinearity and syntenic block analyses, and synonymous substitution rates (Ks) between homologous gene pairs were calculated using PAML’s NG86 and YN00 methods, with Gaussian kernel density estimation applied to visualize WGD signals within and between species.

### Transcriptome profiling

RNA-seq data were processed with fastp v0.23.2^85^ and mapped separately onto both haplotype reference genomes (hap1 and hap2) using HISAT2 v2.2.1^86^. Gene expression levels were quantified using StringTie v2.1.4^87^. Functional enrichment analyses (GO and KEGG) were performed using TBtools v1.098761^88^.

### Identification of biosynthetic gene cluster (BGC)

The BGC were initially identified using plantiSMASH v2.0.0^89^ with default parameters to detect candidate clusters based on gene proximity and conserved domain signatures across both haplotypes (with same parameters) of the *P.chienii* genome. To refine these candidates and confirm functional coordination, we integrated co-expression analysis of metabolism-related genes within each cluster^90,91^. Specifically, Weighted gene correlation network analysis (WGCNA v1.72.5) and Pearson correlation analyses were conducted in R software following the official manual^92^, to quantitatively evaluate the regulatory coherence of these clusters. To construct a highly robust, species-wide co-expression network, we utilized an integrated dataset comprising 124 *P.chienii* transcriptomes. This comprehensive dataset consists of three parts: (1) transcriptome data from a population of 108 *P. chienii* individuals^93^(BioProject ID: PRJNA674020), which represent diverse natural populations distinct from the individual (SQS001) sequenced in this study; (2) additional publicly available *P. chienii* transcriptomic data (BioProject ID: PRJNA630781^94^; PRJEB4922^95^; PRJNA726756^96^; PRJNA396655^97^; PRJNA498605^98^); and (3) the tissue-specific transcriptomes generated in our current study (BioProject ID: PRJCA042793). Transcripts Per Kilobase Million (TPM) data for all genes across these 124 integrated samples served as input matrix. A soft-thresholding power of 9 was selected to achieve an approximate scale-free topology. The majority ( > 80%) of the metabolism-associated genes co-assigned to the same expression module. Clusters meeting this threshold were validated as functionally coherent BGCs, ensuring that regulatory coherence, rather than mere physical contiguity, defined the final boundaries.

### Identification of orthologous and paralogous genes in the paclitaxel biosynthetic pathway

Orthologous sequences in the paclitaxel biosynthetic pathway were identified across *T. mairei*, *P. chienii*, *M. glyptostroboides*, *T. grandis*, *G. biloba*, *C. panzhihuaensis*, *Pinus tabuliformis*, *Cupressus gigantea*, *Taxodium distichum*, *Pinus densiflora*, *Gnetum montanum*, *Physcomitrella patens*, *Amborella trichopoda* and *Arabidopsis thaliana* using the reciprocal best hit (RBH) approach^99^. For each query sequence from *T. mairei*, blastp was employed to search against all protein sequences of the target species with an E-value < 1 × 10⁻⁵. The top-scoring hit in each target sequence was then reciprocally queried against all protein sequences of *T. mairei* using the same parameters. Pairs exhibiting reciprocal best hits and ≥45% amino acid sequence identity were designated as orthologs. To identify paralogous genes, self-blastp searches (E-value < 1 × 10⁻⁵) were conducted within each species’ all protein sequences. Sequences matching an ortholog with ≥90% amino acid sequence identity and ≥90% alignment coverage (excluding self-matches) were classified as paralogs.

### Identification of chromation compartments TADs

Cleaned paired-end Hi-C sequencing reads were processed using the HiC-Pro v3.1.0^100^ and aligned to the Pca genome to construct KR-normalized contact matrices at 50 kb and 500 kb resolutions. Subsequent chromatin structure analyses were performed using the HiCExplorer v3.7.6^101^. A/B compartments were delineated using hicPCA, utilizing eigenvectors to distinguish transcriptionally active A compartments from transcriptionally repressed B compartments. Topologically Associating Domains (TADs) and their hierarchical sub-TADs boundaries were identified using the hicFindTADs tool, based on the parameters “--thresholdComparisons 0.01 --delta 0.25” and “--thresholdComparisons 0.01 --delta 0.05,” respectively. Finally, these results were visualized using PyGenomeTracks v3.9^102^.

### Structure modeling, docking, molecular dynamics simulations, and tunnel analysis

To account for protein flexibility, the initial structures of PcTOT and TmTOT were predicted using ColabFold v1.6.1^103,104^ and subsequently expanded into conformational ensembles via AlphaFlow^105^. Representative conformations maintaining intact active-site architectures were selected for molecular docking using AutoDock Vina v1.2.0^106^. The docking grid was centered on the heme-containing catalytic pocket. Docked poses were evaluated based on both binding scores and catalytic geometries, specifically the distance and corresponding reaction angle between the substrate reactive atom and the catalytic oxygen. Conformations displaying favorable catalytic geometries were retained for structural comparison and downstream molecular dynamics (MD) simulations. The selected PcTOT-substrate and TmTOT-substrate complexes underwent 200-ns MD simulations using GROMACS v2022.6^107^. The resulting trajectories were processed to remove periodic boundary conditions and structurally aligned. Root-mean-square fluctuation (RMSF) profiles were then calculated to assess residue-level flexibility. Regions showing differential local flexibility between the two enzymes were identified and mapped onto their 3D protein structures. Finally, substrate-access tunnels were computed using CAVER v3.0^108^, with the starting point defined at the heme active center adjacent to the docked substrate.

### Cloning of paclitaxel biosynthetic genes and transient expression in *N. benthamiana*

The coding sequences of *T2αH*, *T7βH*, *T7AT*, *TBT*, *TOT* were amplified from *T. mairei* and *P. chienii* cDNA by PCR. Oligonucleotide primers were purchased from Sangon Biotech, and the primer sequences are provided in Supplementary Data 6. The PCR products were inserted into the *Age*I- and *Xho*I- (New England Biolabs) sites of the pEAQ-HT vector. The plasmids containing the genes were transformed into *Agrobacterium tumefaciens* (strain GV3101) cells using the freeze–thaw method^109^. The *Agrobacterium* strains were then introduced into the leaves of *N. benthamiana* plants. Each experiment was tested on leaf 6–8 (numbered by counting from the bottom) of the same *N. benthamiana* plant, as three biological replicates.

### Metabolite extraction of *P. chienii* and *T. mairei* samples

For metabolite extraction, *P. chienii* and *T. mairei* tissues stored at −80°C (5–10 g) were ground in liquid nitrogen to 40–80 mesh and vacuum freeze-dried. Then, 100 mg of the resulting powder was extracted with 1.5 mL of 80% methanol. For the sample batches designated for the 7500 system, the extraction solvent was explicitly spiked with 100 ng mL^−1^ paclitaxel-*d*_5_ as a stable isotope internal standard to dynamically compensate for extraction loss and complex matrix effects. For the batches designated for the 5500 system, parallel extraction was performed without the addition of the internal standard. The mixtures were extracted by ultrasonication for 1 h at room temperature, followed by overnight incubation at 4 °C. The extracts were centrifuged at 12,000 × *g* for 10 min, and the supernatant was collected and recentrifuged. To remove any particulate matter prior to analysis, the final supernatants were filtered through a 0.22-μm nylon syringe filter and directly transferred to autosampler vials for liquid chromatography-mass spectrometry (LC-MS) injection.

### Metabolite extraction of *N. benthamiana* leaves

Five days after *Agrobacterium* infiltration, *N. benthamiana* leaf tissue was collected using a leaf disc cutter (1 cm in diameter) and placed inside a 2-mL tube. Each biological replicate consisted of four disks from the same leaf, corresponding to approximately 40 mg fresh weight. For metabolite extraction, 500 μL of chilled 75% acetonitrile in water (v/v, high-performance liquid chromatography (HPLC) grade; Fisher Chemical) was added to each sample, along with one 5-mm stainless steel bead. The mixture was thoroughly homogenized using a tissue lyser at 30 Hz for 60 s to ensure complete cell lysis. Subsequently, the homogenates were centrifuged at 12,000 × *g* for 10 min at 4 °C to pellet the tissue debris. The resulting supernatants were filtered using 96-well hydrophilic PTFE filters with a pore size of 0.45 μm (Millipore) and then transferred into autosampler vials for downstream targeted metabolite detection on the SCIEX 5500 QTRAP system using the external standard method.

### LC-MS analysis

Targeted metabolite detection and trace quantification of taxane compounds were strictly performed in accordance with the authoritative group standard “Quantitative detection guidelines for taxanes in *Taxus* species” (T/CI 1115-2025)^110^. The quantitative analyses were performed on a SCIEX 5500 QTRAP® triple-quadrupole/linear-ion-trap hybrid mass spectrometer equipped with a Turbo-V™ ion source and a Shimadzu LC-2030 UHPLC system. For the 5500 system, chromatographic separation was achieved on a Waters ACQUITY UPLC BEH T3 column (2.1 × 100 mm, 1.8-μm) maintained at 40 °C. Mobile phases consisted of (A) 4 mM ammonium acetate in water and (B) 4 mM ammonium acetate in methanol, delivered at 0.3 mL min⁻¹. A 32-min gradient was programmed as follows: 0–2.5 min 10 → 15% B, 2.5–26 min 15 → 90 % B, 26–26.1 min 90 → 98 % B, 26.1–28 min 98 % B, 28–28.1 min 98 → 10 % B, 28.1–30 min 10 % B, followed by 2 min of re-equilibration. Injection volume was 5 µL and samples were maintained at 8 °C in the autosampler.

To rigorously cross-validate the trace detection of paclitaxel at extreme low abundance, independent targeted analyses were also conducted on a higher-sensitivity SCIEX 7500 Triple Quad mass spectrometer coupled with an ExionLC^TM^ system (AB SCIEX, USA), which was equipped with a heated electrospray ionization (HESI) probe. For the 7500 system, chromatographic separation was performed on a Waters ACQUITY UPLC HSS T3 column (2.1 × 100 mm, 1.8 µm) maintained at 40 °C. The mobile phases consisted of (A) 4 mM ammonium acetate in water and (B) 4 mM ammonium acetate in methanol, delivered at a flow rate of 0.3 mL min-1. A 20-min gradient program was applied as follows: 0−2.5 min, 10 → 15% B; 2.5−5 min, 15 → 40% B; 5−7 min, 40 → 70% B; 7−8 min, 70% B; 8−11 min, 70 → 80% B; 11−13 min, 80 → 100% B; 13−16 min, 100% B;16−16.5 min, 100 → 10% B; followed by 3.5 min of re-equilibration. The injection volume was 2 µL, and the autosampler temperature was maintained at 8 °C.

The 5500 mass spectrometer was operated in positive electrospray ionization (ESI⁺) mode with multiple-reaction monitoring (MRM) using compound-specific transitions optimized via direct infusion of authentic standards. Quantification on the 5500 system was established using the external standard method. To unambiguously exclude matrix-derived false positives, enhanced product ion (EPI) scans were triggered on the 5500 QTRAP system to acquire full tandem mass spectrometry (MS/MS) fragmentation spectra, utilizing its linear-ion-trap functionality to structurally confirm the exact identity of trace paclitaxel against authentic standards.

In parallel, the ultra-sensitive 7500 system was dedicated to trace quantification of low-abundance samples utilizing the stable-isotope (paclitaxel-d₅) internal standard method in multiple reaction monitoring (MRM) mode under positive ionization, with ion transitions optimized using chemical standards. Source parameters for the 5500 system were: curtain gas 25 psi, ion-spray voltage 5.5 kV, temperature 500 °C, nebulizer gas (GS1) 50 psi, and heater gas (GS2) 50 psi. Source parameters for the 7500 system were: curtain gas 40 psi, ion-spray voltage 2.5 kV, temperature 400 °C, nebulizer gas (GS1) 35 psi, and heater gas (GS2) 60 psi. Compound-dependent parameters (including declustering potential, collision energy, and cell-exit potential) were automatically optimized with Analyst® 1.7 and SCIEX OS software.

Calibration curves (0.5–1000 ng mL⁻¹) of taxinine J (CAS: 18457-46-0), taxusin (CAS: 19605-80-2), Baccatin III (CAS: 27548-93-2) and paclitaxel (CAS: 33069-62-4) standards, as well as other target metabolites analyzed in *N. benthamiana* leaves, were prepared by serial dilution of authentic standards in 80% methanol to match the sample solvent matrix. For the 7500 system calibration, the standard solutions were consistency spiked with 20 ng mL^−1^ paclitaxel-*d*_5_ to match the internal standard concentration in the samples. Quality-control pools (low, mid, high) were injected every 10 samples to actively monitor system stability and verify the absence of background interference; intra-day RSDs were <14.8 %. Data acquisition and quantification were performed with Analyst 1.7 and MultiQuant 3.0 softwares for the 5500 system, applying external standard calibration. For the 7500 system, SCIEX OS software was applied for taxinine J, taxusin, Baccatin Ⅲ and paclitaxel identification and peak integration, utilizing internal-standard normalization with a 1/x² weighting factor.

### GC-MS analysis of taxadiene and Taxa-4(20),11-dien-5α-ol

For metabolite extraction, the plant tissues ground in liquid nitrogen were accurately weighed (approximately 20 mg) into a 1.5-mL centrifuge tube, followed by the addition of 1 mL of ethyl acetate. The mixture was vortexed for 3 min and subjected to ultrasonication at 25 °C and 200 W for 30 min. The sample was then centrifuged at 12,000 × *g* for 5 min. A 1-mL aliquot of the supernatant was precisely transferred to a separate tube containing 100 mg of anhydrous magnesium sulfate (MgSO_4_) to remove residual moisture and polar matrix interferences. After vortexing for 3 min and centrifuging at 12,000 × *g* for 5 min, the final supernatant was filtered through a 0.22-μm organic nylon syringe filter and transferred directly into an autosampler vial for gas chromatography-mass spectrometry (GC-MS) analysis.

Quantitative analysis was performed on a Thermo Scientific TSQ 9000 triple-quadrupole mass spectrometer coupled with a Trace 1310 gas chromatograph system. Chromatographic separation was achieved on a DB-5MS UI capillary column (30 m × 0.25 mm, 0.25 m film thickness; Agilent Technologies). The injection volume was 1 µL with a split ratio of 20:1. High-purity helium (99.999%) was used as the carrier gas at a constant flow rate of 1.2 mL min, and the injector temperature was maintained at 270 °C. The oven temperature program was set as follows: the initial temperature was held at 100 °C for 1 min, then increased to 175 °C at a rate of 15 °C/min, subsequently raised to 220 °C at a rate of 4 °C/min and kept constant for 2 min, and finally raised to 290 °C at a rate of 20 °C/min and held constant for 5 min. The MS transfer line and electron ionization (El) source temperatures were set at 250 °C and 300 °C, respectively. The mass spectrometer was operated in EI mode at 70 eV, utilizing selected ion monitoring (SIM) for the highly sensitive detection of target compounds.

Calibration curves for taxadiene (CAS: 109808-37-9) and taxa-4(20),11-dien-5α-ol (CAS: 148381-56-0) were prepared by serial dilution of authentic standards in a hexane:ethyl acetate mixture (4:1, v/v) to yield concentrations of 0.01, 0.02, 0.05, 0.1, 0.2, 0.5,1,2, 5,10, 20, 50, and 100 μg mL^−1^. The standard solutions were injected sequentially, and data acquisition and quantification were performed using the Chromeleon software suite. Calibration curves were constructed by plotting peak areas against the corresponding analyte concentrations to establish linear regression equations, which were subsequently used to calculate the absolute contents of the two target taxanes in the samples.

### Reporting summary

Further information on research design is available in the Nature Portfolio Reporting Summary linked to this article.

## Supplementary information

Supplementary Information

Peer Review file

Description of Additional Supplementary Files

Supplementary Data 1

Supplementary Data 2

Supplementary Data 3

Supplementary Data 4

Supplementary Data 5

Supplementary Data 6

Reporting Summary

## Source data

Source Data

## Data Availability

All sequencing data, including genomic sequencing, Hi-C, RNA-seq, and Revio HiFi reads, have been deposited in the NGDC Sequence Read Archive (SRA) under BioProject PRJCA042793. The de novo genome assembly and annotation have been deposited in Figshare (https://doi.org/10.6084/m9.figshare.30340690). The mass spectrometry-based metabolomics data, including LC-MS/MS and GC-MS data, have been deposited in OMIX under accession number OMIX019547 (https://ngdc.cncb.ac.cn/omix/release/OMIX019547). Source data are provided with this paper.

## Code availability

The custom scripts and pipelines used for the genome assembly, haplotype phasing, and evolutionary analyses of paclitaxel biosynthesis in this study are publicly available in the GitHub repository (https://github.com/wanghuan766/Pseudotaxus-chienii-genome-project) and also archived on *Zenodo* (https://doi.org/10.5281/zenodo.21839213)^111^.
