# Supplementary material for: Archipelago architecture and evolution of paclitaxel biosynthesis revealed by the Pseudotaxus chienii haplotype-resolved genome
Source: Nat Commun. 2026 Aug 26;17:10189. doi: 10.1038/s41467-026-77115-w (PMC13612588; doi:10.1038/s41467-026-77115-w)
Supplement: Supplementary file 2 [file 41467_2026_77115_MOESM1_ESM.pdf]

Archipelago architecture and evolution of paclitaxel biosynthesis revealed by the *Pseudotaxus chienii* haplotype-resolved genome

Wang *et al.*

Supplementary Table 1. Statistics of flow cytometry estimation of genome sizes.

Type	R1		R2		R3	
	**PI-A Mean**	**PI-A %CV**	**PI-A Mean**	**PI-A %CV**	**PI-A Mean**	**PI-A %CV**
P1 (*P. chienii*)	84,015	2.9	84,410	2.6	82,777	3.0
P2 (*T. mairei*)	54,795	3.1	55,407	3.5	54,128	3.4
Size (Gb)	15.69		15.58		15.64	
Mean size (Gb)			15.64			

Supplementary Table 2. Data statistics of sequencing data for *Pseudotaxus chienii.*

**Libraries**	**Total length**	**N50**	**Sequencing depth (X)**
Illumina	1530.75 Gb	150 bp	99.20
Revio HiFi	677,65 Gb	-	47.25
Hi-C	2336.39Gb	150 bp	151.42
Iso-seq	54.42 Gb	1,707 bp	-
RNA-seq (Bark)	23.96 Gb	150 bp	-
RNA-seq (Leaf)	20.30 Gb	150 bp	-

Supplementary Table 3. Summary statistics of *Pseudotaxus chienii* genome assembly.

**Type**	**Hap1**	**Hap2**
Number of Chromsome	12	12
Total contigs	725	817
Total bases	15,434,027,173	15,430,351,069
Min sequence length	26,284	25,588
Max sequence length	221,615,877	198,625,822
Average sequence length	21,288,313.3	18,886,598.6
Median sequence length	8,783,645	7,280,980
Contig N50 length	60,233,647	55,924,683
QV	54.16 (Hap1: 54.03; Hap2: 54.29)	
K-mer completeness	98.13% (Hap1: 87.63%; Hap2: 87.58%)	
Number of genes	36488	36360
Number of Proteins	38433	38105
BUSCO (pep)	95.6% (Hap1: 95%; Hap2: 95%)	

Supplementary Table 4. Transposable elements in hap1 from *Pseudotaxus chienii.*

Class	Number of elements	Length of sequence	Percentage of sequence
Retroelements	10,737,189	8,801,842,901bp	57.03%
SINEs	82,471	14,602,442bp	0.09%
Penelope	293,281	98,464,596bp	0.64%
LINEs	728,001	507,350,709bp	3.29%
CRE/SLACS	3,517	7,090,517bp	0.05%
L2/CR1/Rex	52,165	32,964,411bp	0.21%
R1/LOA/Jockey	19,202	9,398,204bp	0.06%
R2/R4/NeSL	0	0bp	0.00%
RTE/Bov-B	55,010	38,957,546bp	0.25%
L1/CIN4	486,336	323,206,095bp	2.09%
LTR elements	9,926,717	8,279,889,750bp	53.65%
BEL/Pao	25,226	10,932,175bp	0.07%
Ty1/Copia	2,500,000	1,373,516,193bp	8.90%
Gypsy/DIRS1	6,554,900	6,561,191,329bp	42.51%
Retroviral	123,830	61,875,606bp	0.40%
DNA transposons	9,327,934	3,326,615,269bp	21.55%
hobo-Activator	2,173,153	691,981,030bp	4.48%
Tc1-IS630-Pogo	2,332,523	765,470,860bp	4.96%
MULE-MuDR	364,709	310,530,881bp	2.01%
PiggyBac	26,464	7,273,156bp	0.05%
Tourist/Harbinger	891,390	171,798,776bp	1.11%
Rolling-circles	137,888	72,482,522bp	0.47%
Unclassified	4,858,209	1,421,029,784bp	9.21%
Total interspersed repeats	-	13,647,952,550bp	88.43%

Supplementary Table 5. Transposable elements in hap2 from *Pseudotaxus chienii.*

Class	Number of elements	Length of sequence	Percentage of sequence
Retroelements	10,876,223	8,900,585,527bp	57.68%
SINEs	89,995	14,990,856bp	0.10%
Penelope	166,843	59,119,162bp	0.38%
LINEs	866,795	544,505,237bp	3.53%
CRE/SLACS	0	0bp	0.00%
L2/CR1/Rex	53,351	29,844,318bp	0.19%
R1/LOA/Jockey	9,588	6,842,046bp	0.04%
R2/R4/NeSL	4,251	1,129,036bp	0.01%
RTE/Bov-B	112,013	52,145,641bp	0.34%
L1/CIN4	602,727	393,141,876bp	2.55%
LTR elements	9,919,433	8,341,089,434bp	54.05%
BEL/Pao	26,891	13,426,815bp	0.09%
Ty1/Copia	2,642,609	1,459,124,562bp	9.46%
Gypsy/DIRS1	6,542,651	6,555,747,199bp	42.48%
Retroviral	104,329	46,677,923bp	0.30%
DNA transposons	8,834,642	3,259,035,236bp	21.12%
hobo-Activator	2,182,086	707,612,059bp	4.59%
Tc1-IS630-Pogo	2,188,641	745,176,623bp	4.83%
MULE-MuDR	381,000	320,002,167bp	2.07%
PiggyBac	55,426	30,994,874bp	0.20%
Tourist/Harbinger	1,434,903	440,855,821bp	2.86%
Rolling-circles	114,923	59,318,612bp	0.38%
Unclassified	4,831,827	1,453,280,461bp	9.42%
Total interspersed repeats	-	13,672,020,386bp	88.60%

Supplementary Table 6. Gene function annotations information of two haplotype genomes.

Database	Hap1 gene hits	Hap2 gene hits
Nr	34,332	34,136
InterPro	32,976	32,801
eggNOG	30,145	30,011
Pfam	26,872	26,654
SwissProt	24,563	24,383
GO	31,893	31,796
KEGG	9,744	9,736

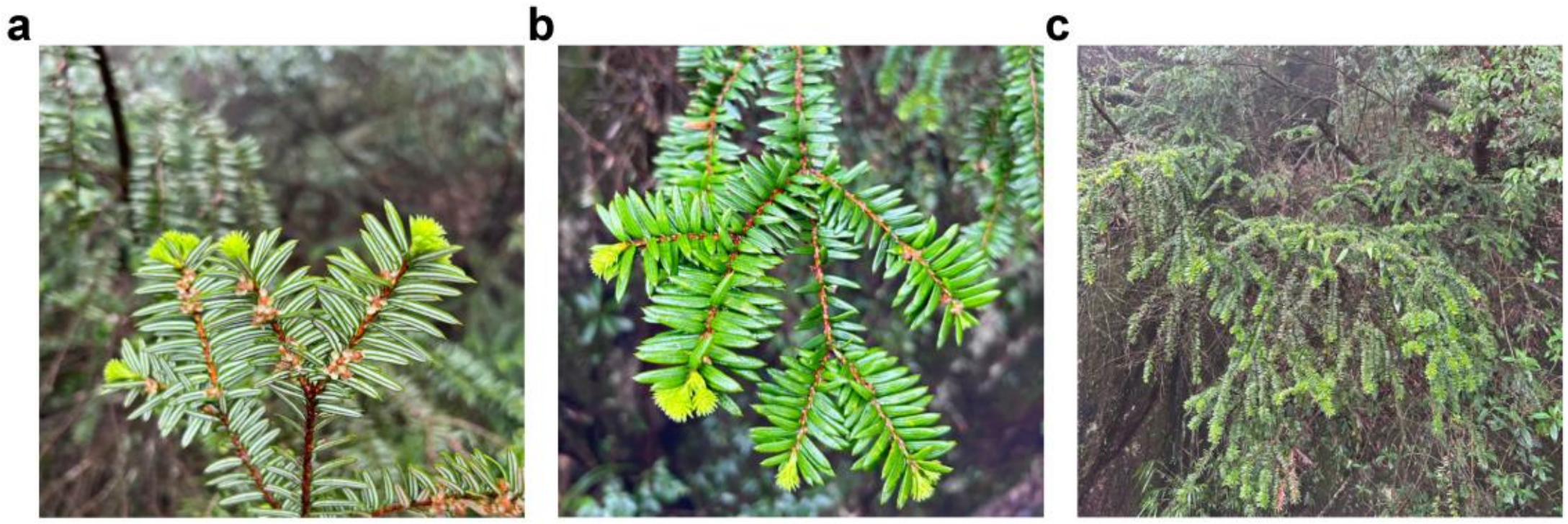

Supplementary Fig. 1. Morphological characteristics and habitats of sequenced *Pseudotaxus chienii*. a *P. chienii* branchlet showing the two distinct white stomatal bands on the abaxial surface of the needles and terminal pollen cones. **b** Shoot tip highlighting the light-green new growth. **c** The overall growth habit of the *P. chienii* in its natural habitat.

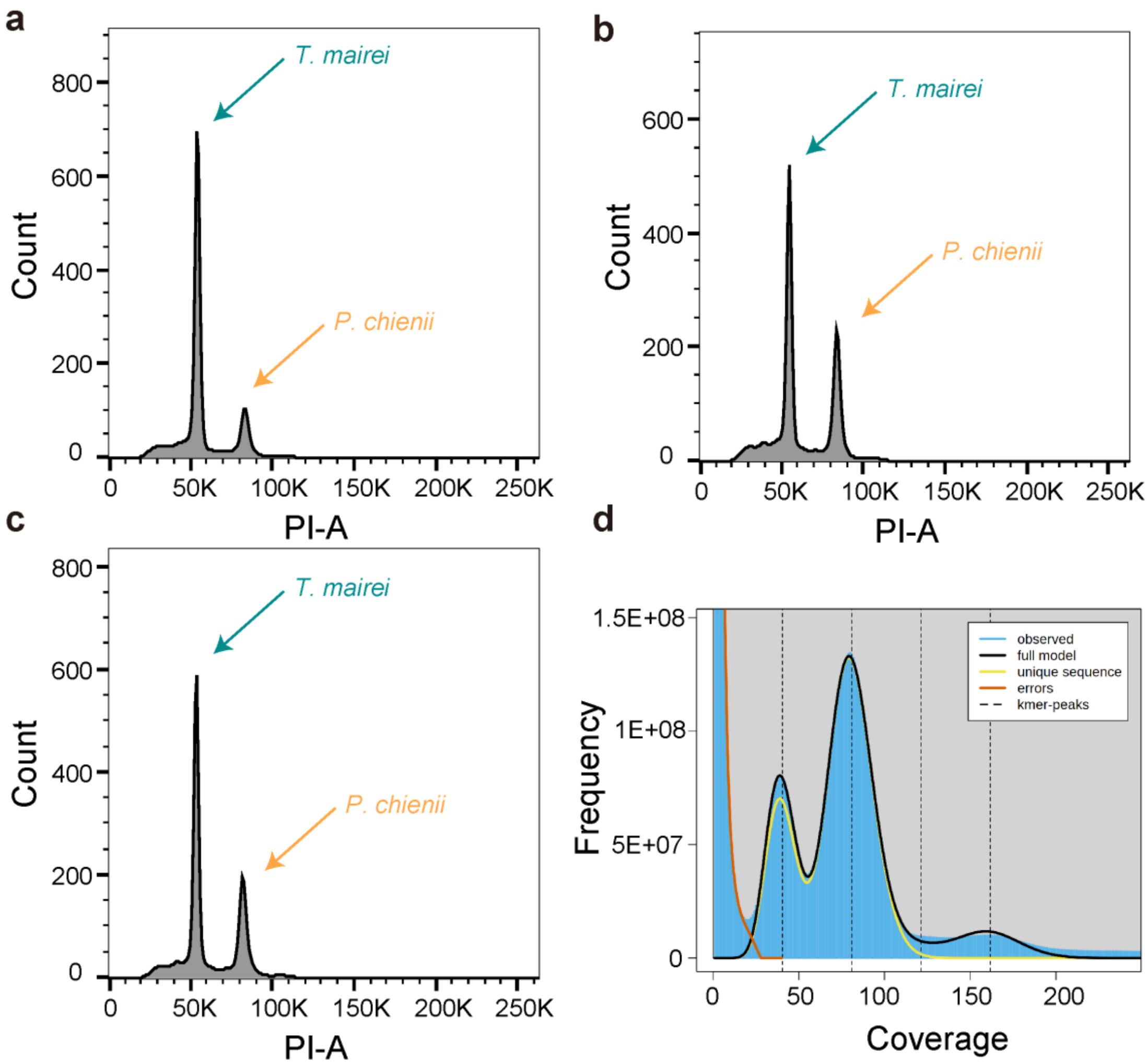

Supplementary Fig. 2. Genome size assessment. a-c Flow cytometry results of genome size estimation. a, b, c are three experimental replicates, peak 2 represents the internal reference of *T. mairei* (10.23Gb), and peak represents *P. chienii*. PI-A, propidium iodide area. **d** K-mer-based genome size prediction. The first peak presents heterozygous K-mers and the second peak presents homozygous K-mers. The estimated monoploid genome sizes and heterozygosities are shown. The first peaks of both results are located at 40 ×, corresponding to second peaks at 80 ×. Source data are provided as a Source Data file.

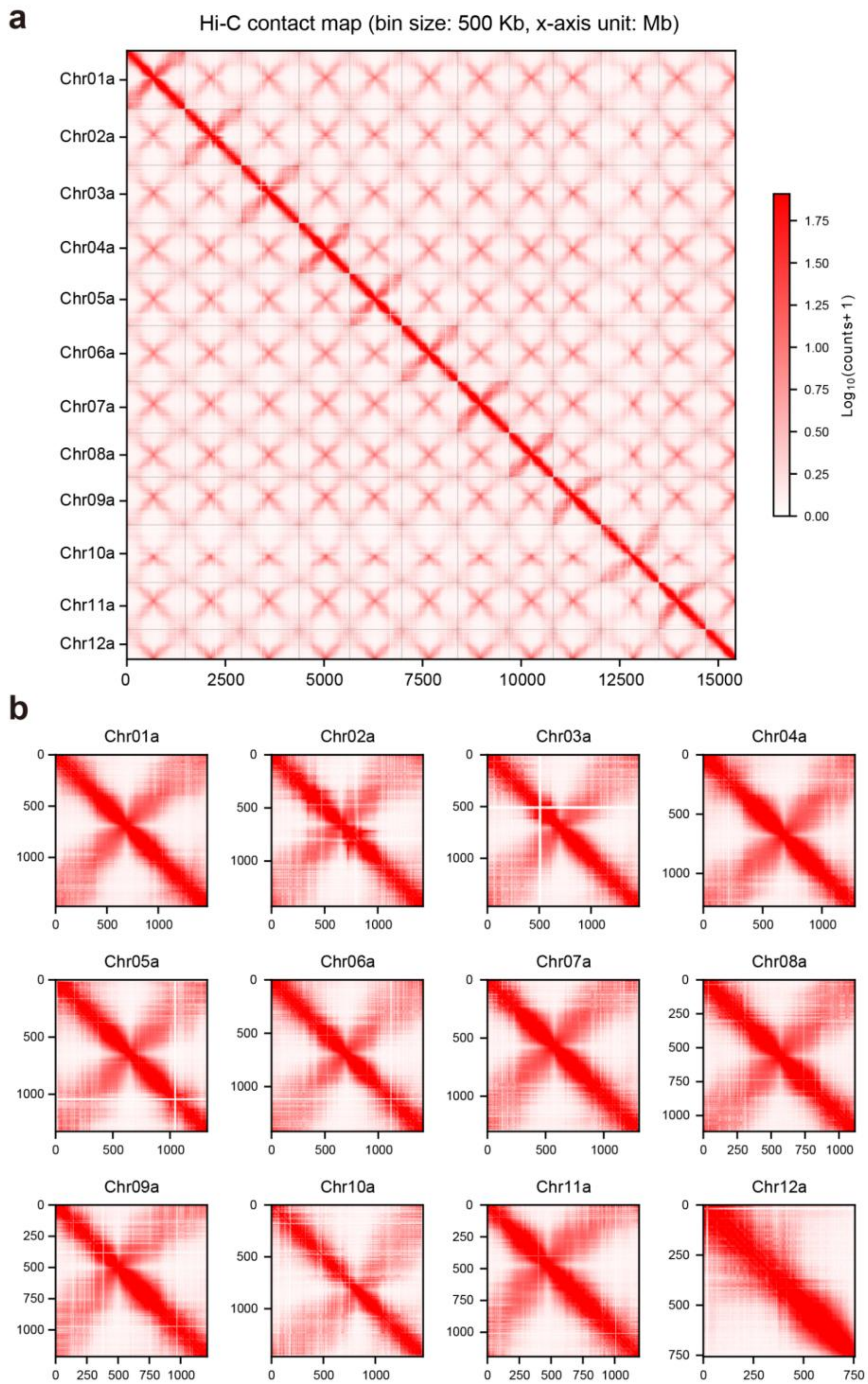

Supplementary Fig. 3. Hi-C heatmaps of chromosomal interactions for Hap1. **a** 12 chromosomal genome heatmap. Both axes represent the 12 chromosomes of Hap1 (Chr01–Chr12) binned at 500 kb resolution; color scale indicates normalized interaction frequency (observed/expected). **b** Separate 12 single chromosome heatmap. Axes correspond to physical position (Mb) along the indicated single chromosome binned at 500 kb; color scale as in panel a, depicting intrachromosomal interaction intensity.

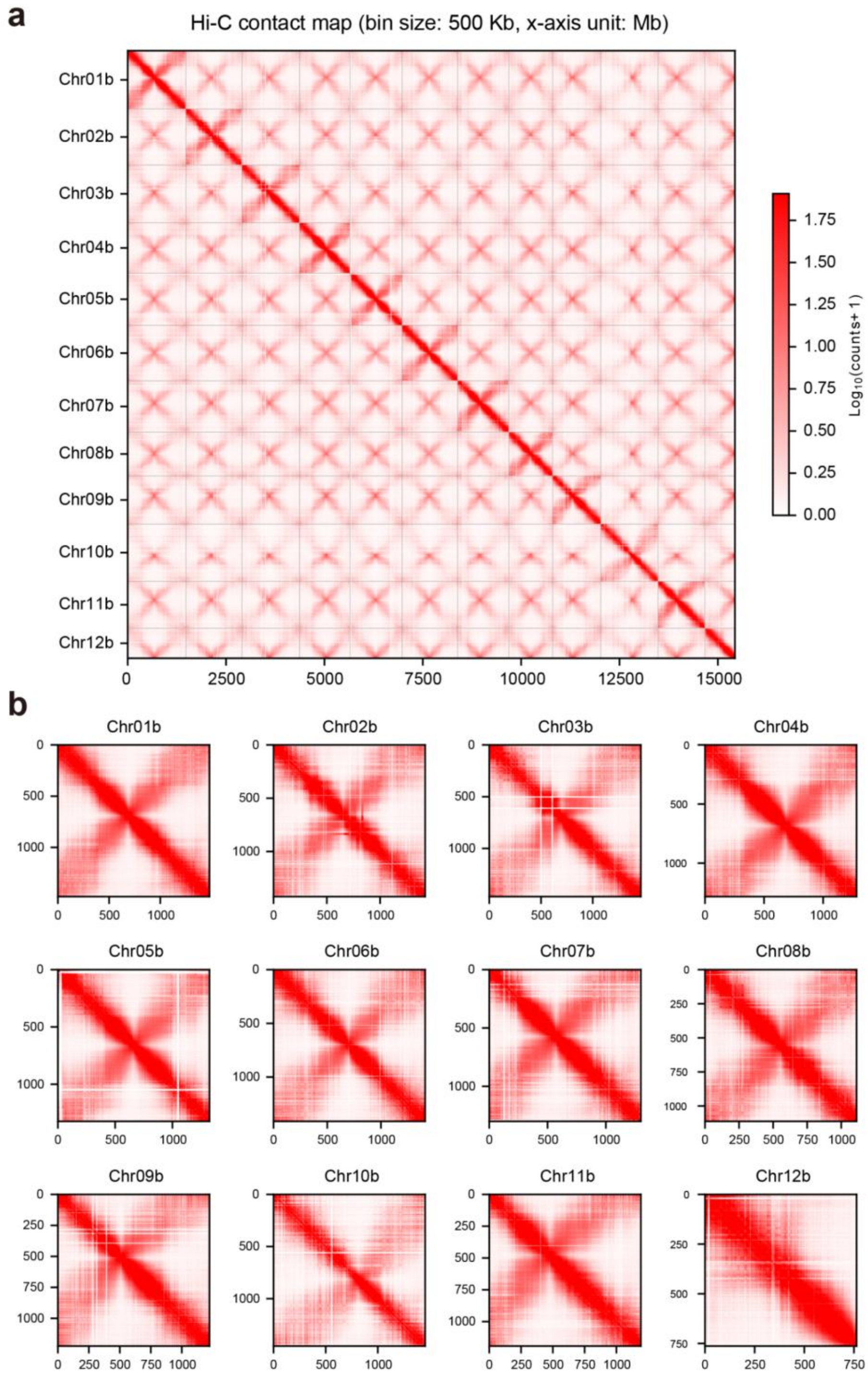

Supplementary Fig. 4. Hi-C heatmaps of chromosomal interactions for Hap2. **a** 12 chromosomal genome heatmap. Both axes represent the 12 chromosomes of Hap1 (Chr01–Chr12) binned at 500 kb resolution; color scale indicates normalized interaction frequency (observed/expected). **b** Separate 12 single chromosome heatmap. Axes correspond to physical position (Mb) along the indicated single chromosome binned at 500 kb; color scale as in panel a, depicting intrachromosomal interaction intensity.

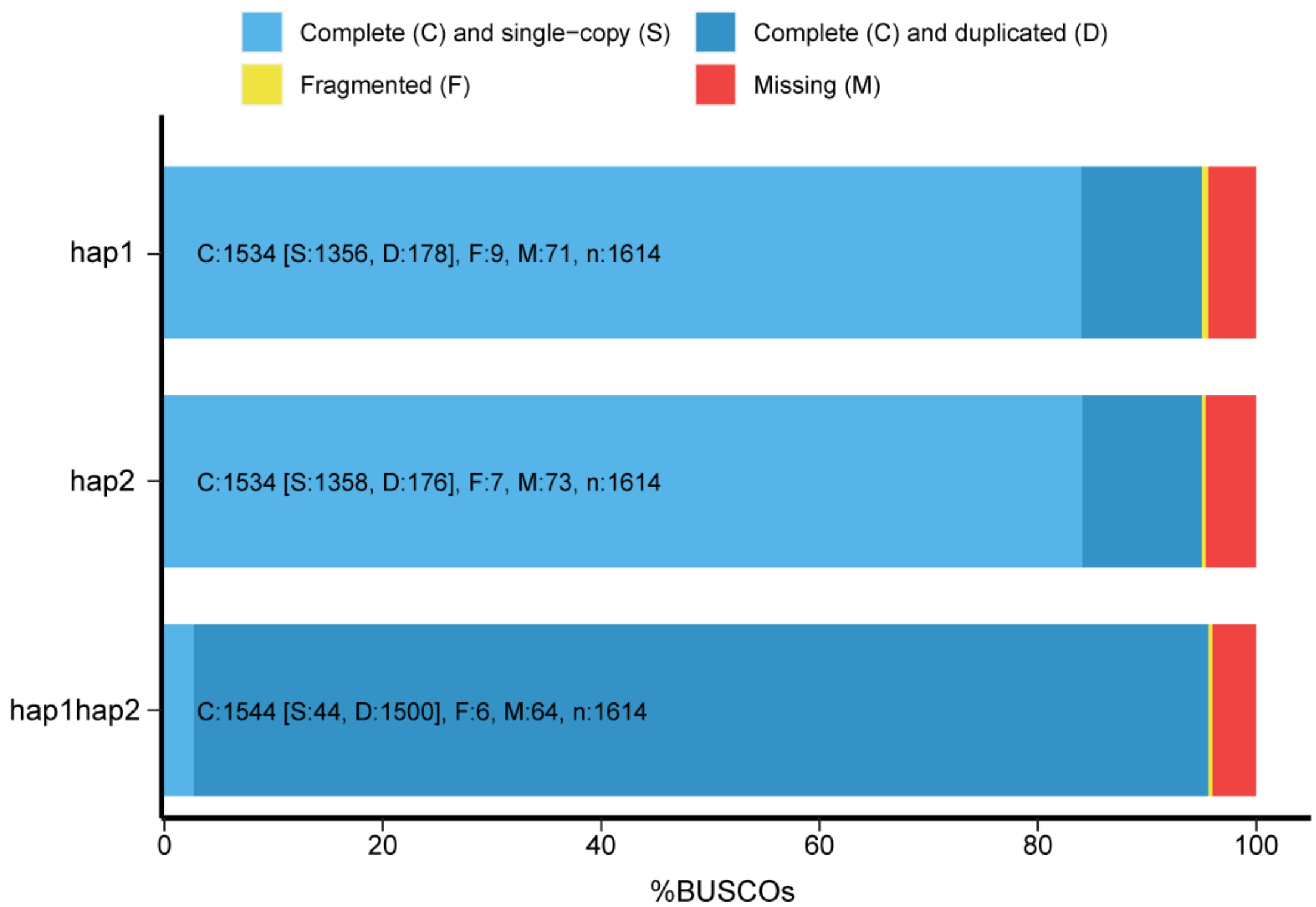

Supplementary Fig. 5. BUSCO evaluation of *Pseudotaxus chienii* protein sequences. Hap1, hap2, and hap1hap2 are derived from *P. chienii* hap1, *P. chienii* hap2 and the merged hap1 and hap2, respectively.

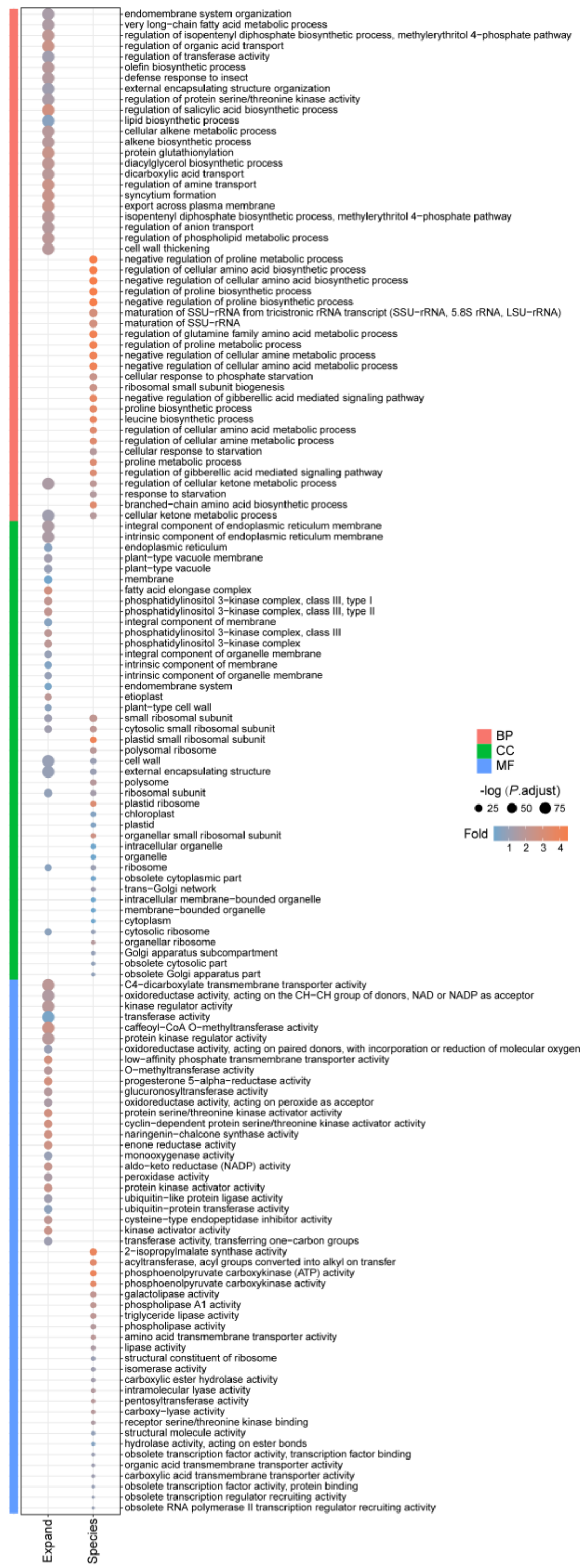

Supplementary Fig. 6. Gene Ontology (GO) enrichment of the expanded and specific gene families in *Pseudotaxus chienii*. BP, biological process; MF, molecular function; CC, cellular component. The *P* values were calculated using hypergeometric distribution (two-sided) and then adjusted using the FDR. Source data are provided as a Source Data file.

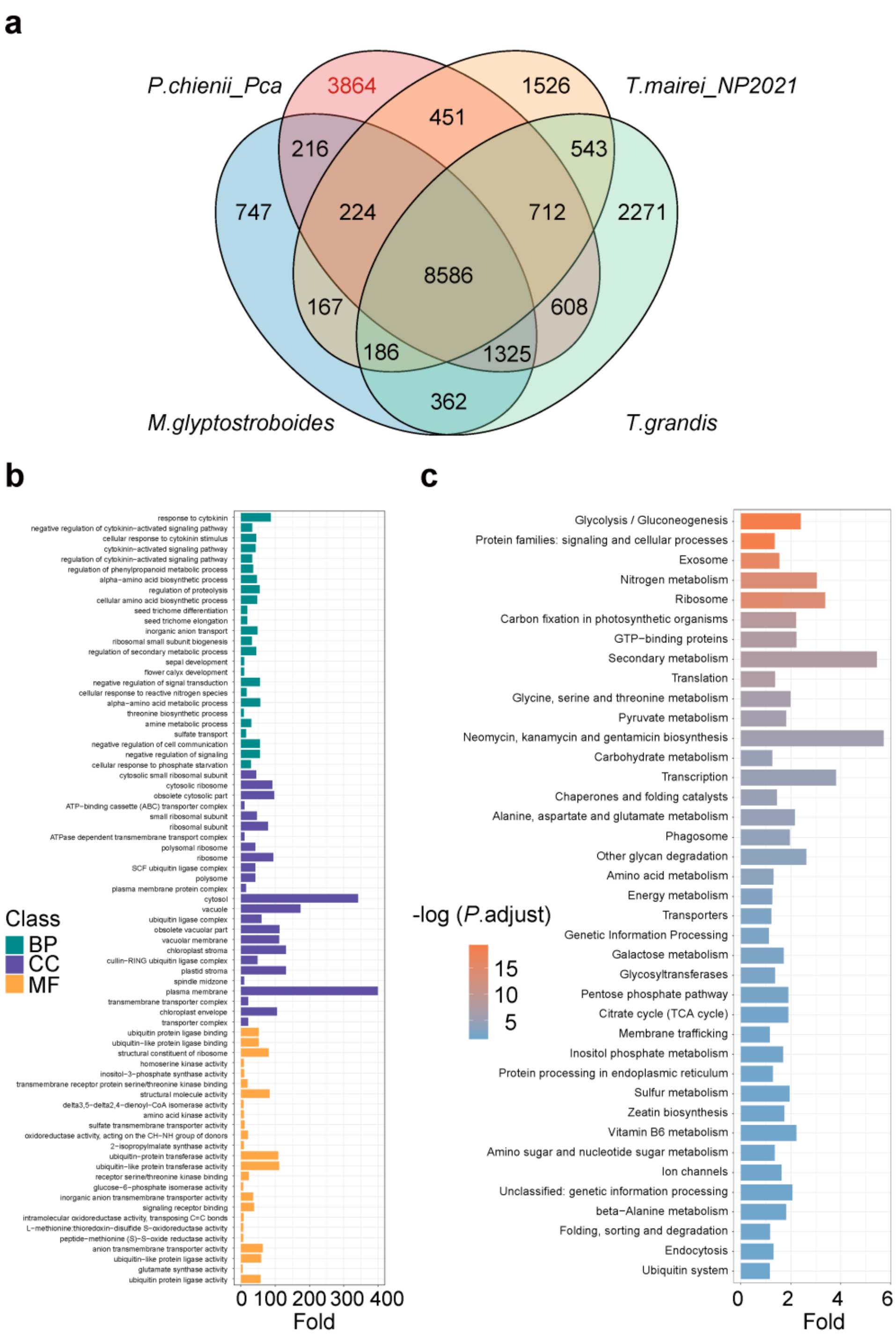

Supplementary Fig. 7. Comparative genomic analysis of *Pseudotaxus chienii* and related species. a The Venn diagram illustrates the distribution of orthologous gene families shared between *P. chienii* and three closely related species. **b** GO pathway enrichment analysis of species-specific genes in *P. chienii*. BP, biological process; MF, molecular function; CC, cellular component. **c** KEGG pathway enrichment analysis of species-specific genes in *P. chienii*. The *P* values were calculated using hypergeometric distribution (two-sided) and then adjusted using the FDR. Source data are provided as a Source Data file.

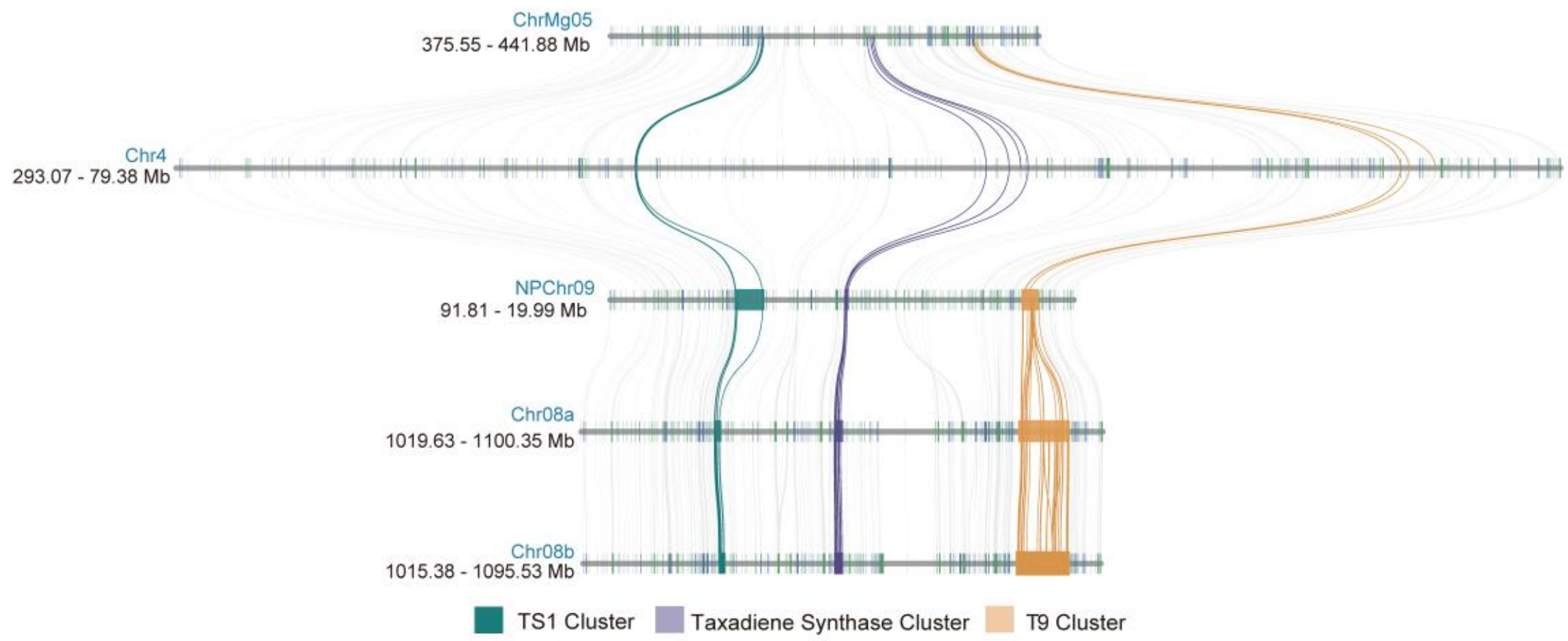

Supplementary Fig. 8. Homologous gene pairs of *Pseudotaxus chienii* and three closely related species. Curved lines connect homologous genes pairs across the following chrommosomal sgments (reverse coordinates indicate opposite orientation). ChrMg05 (375.55–441.88 Mb) comes from *M. glyptostroboides*, Chr4 (293.07–79.38 Mb) from *T. grandis*, NPChr09 (91.81–19.99 Mb) from *T. mairei*, Chr08a (1019.63–1100.35 Mb) from *Pseudotaxus chienii* (hap1), and Chr08b (1015.38–1095.53 Mb) from *Pseudotaxus chienii* (hap2).

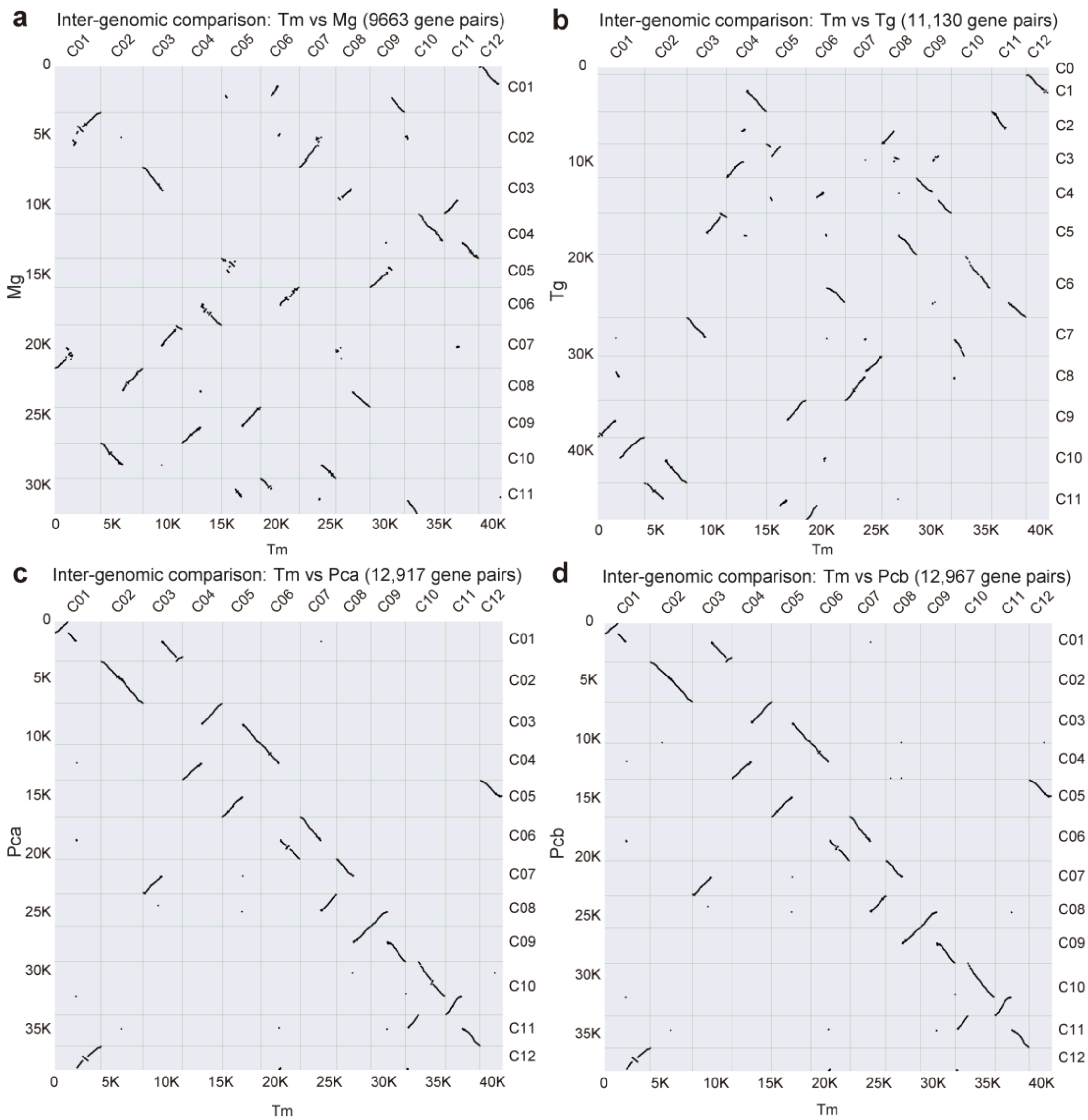

Supplementary Fig. 9. Genome dot plot analysis of *Pseudotaxus chienii* and three closely related species. **a** *T. mairei* (Tm) vs *M. glyptostroboides* (Mg) (8,857 orthologous gene pairs). **b** *T. mairei* (Tm) vs *T. grandis* (Tg) (10,084 orthologous gene pairs). c *T. mairei* (Tm) vs *Pseudotaxus chienii* hap1 (Pca) (12,303 orthologous gene pairs). d *T. mairei* (Tm) vs *Pseudotaxus chienii* hap2 (Pcb) (12,341 orthologous gene pairs). In each panel, the x-axis represents chromosomes (C01 to C12) of Tm, scaled cumulatively from 0 to ~40,000 units (bp). The y-axis represents the chromosomes of the compared genomes (C01 to C11 or C12, depending on the species) on the same scale bar. Black dots indicate the positions of orthologous gene pairs, and diagonal lines indicate conserved homologous regions.

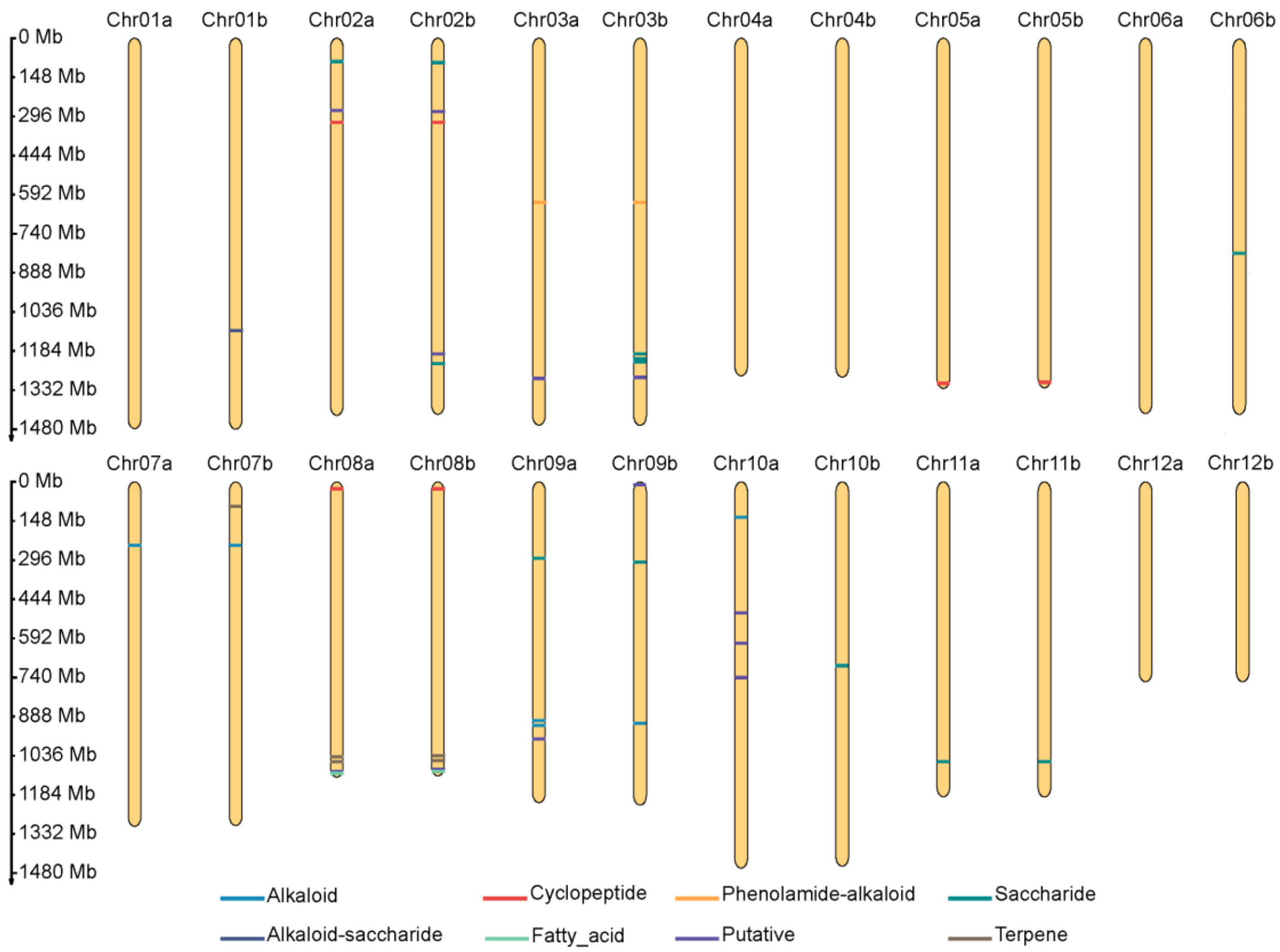

Supplementary Fig. 10. The chromosomal distribution of secondary metabolism-related gene clusters in *Pseudotaxus chienii*. Color bars and labels indicate key enzyme-coding genes involved in pathways such as esters (putative), lignans, alkaloids, terpenes, and polyketides.

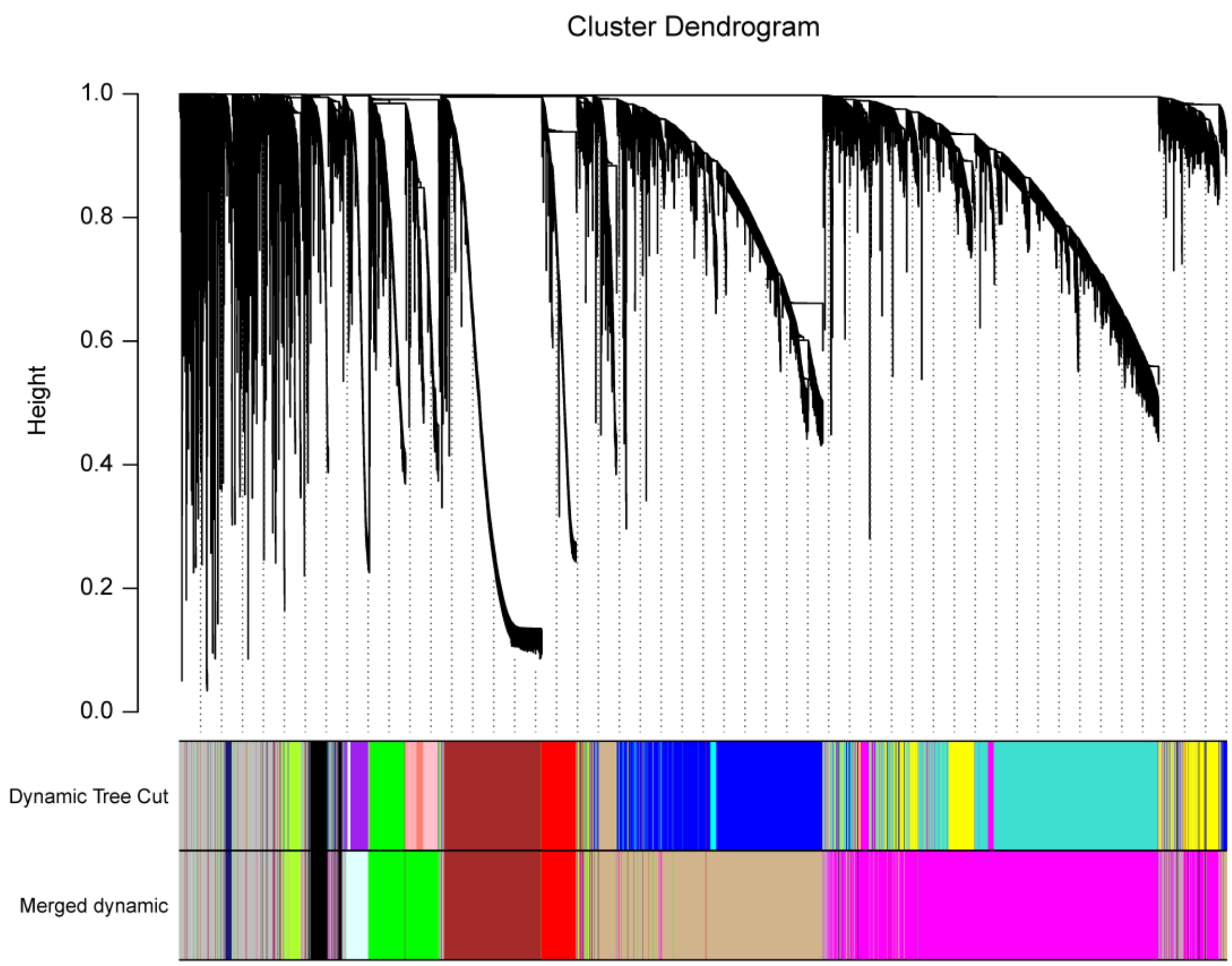

Supplementary Fig. 11. Weighted gene co-expression network analysis (WGCNA) of *Pseudotaxus chienii* transcriptomes. The dendrogram was generated using expression profiles from 124 RNA-seq libraries derived from multiple *P. chienii* tissues, developmental stages, and treatment conditions. All expressed genes passing quality filtering were included in the network construction and module clustering analysis. Branches group genes into distinct co-expression modules identified by topological overlap-based hierarchical clustering. Colored bars below the dendrogram represent different co-expression modules and correspond to the module assignments provided in Supplementary Data 3. The transcriptomic dataset used for WGCNA consisted of both newly generated RNA-seq data from this study and previously published datasets, which were reanalyzed under a unified computational pipeline. Scale bar, Pearson correlation distance.

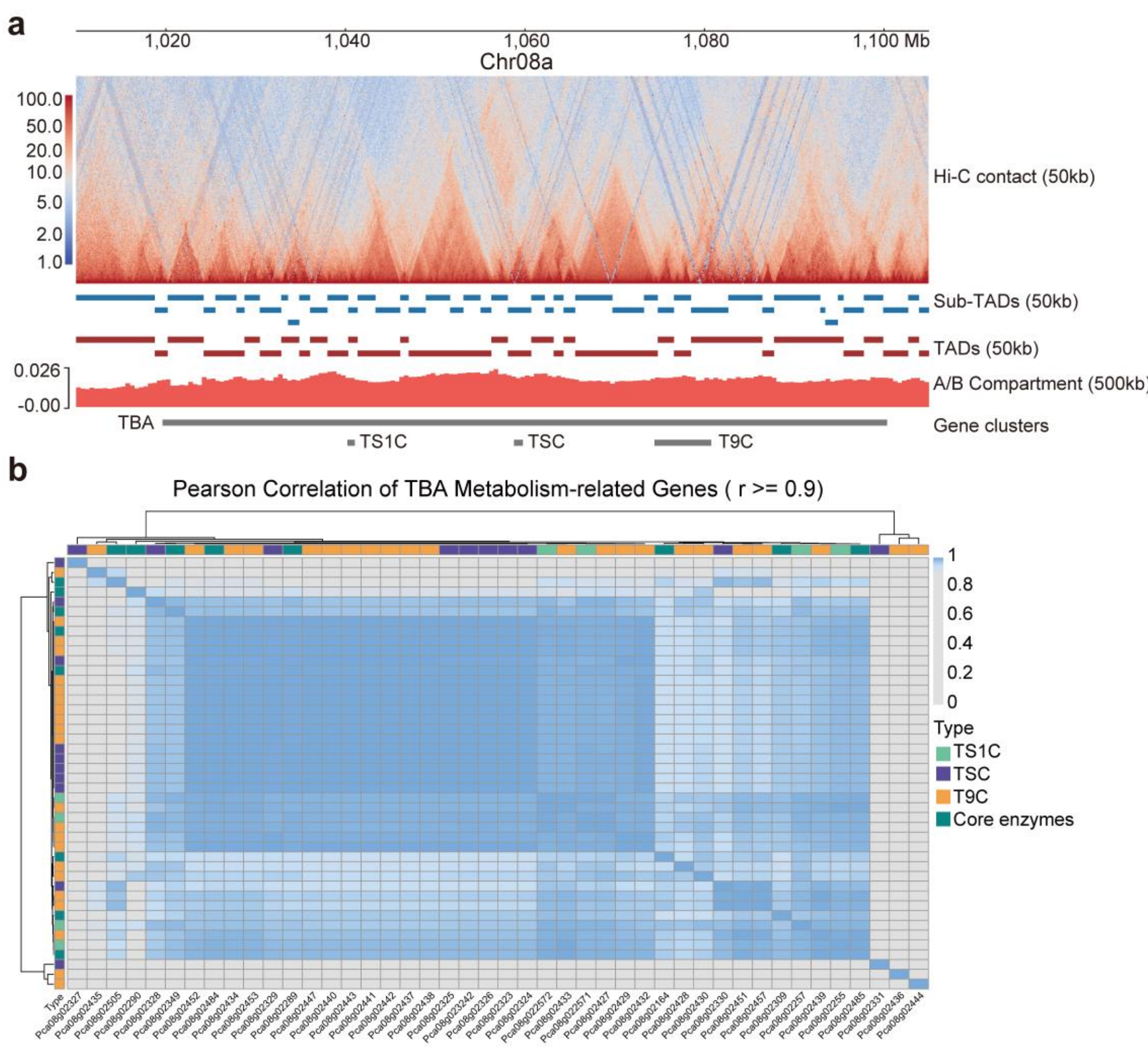

Supplementary Figure 12. Chromatin structure and co-expression network of metabolism-related genes in the TBA region of Pca. a Hi-C interaction map and chromatin features in the TBA region. Each track (from top to bottom) shows the Hi-C contact frequency, sub-TADs, TADs, A/B compartments, TBA, and the genomic location of the three gene clusters (TS1 Cluster (TS1C), Taxadiene Synthase Cluster (TSC), and T9 Cluster (T9C)). **b** Pearson correlation heatmap of metabolism-related genes in TBA. Based on a transcriptome dataset containing 124 samples, these genes collectively form a highly coordinated co-expression network, with the vast majority of gene pairs having a Pearson correlation coefficient (r $\geqslant$ 0.9). Source data are provided as a Source Data file.

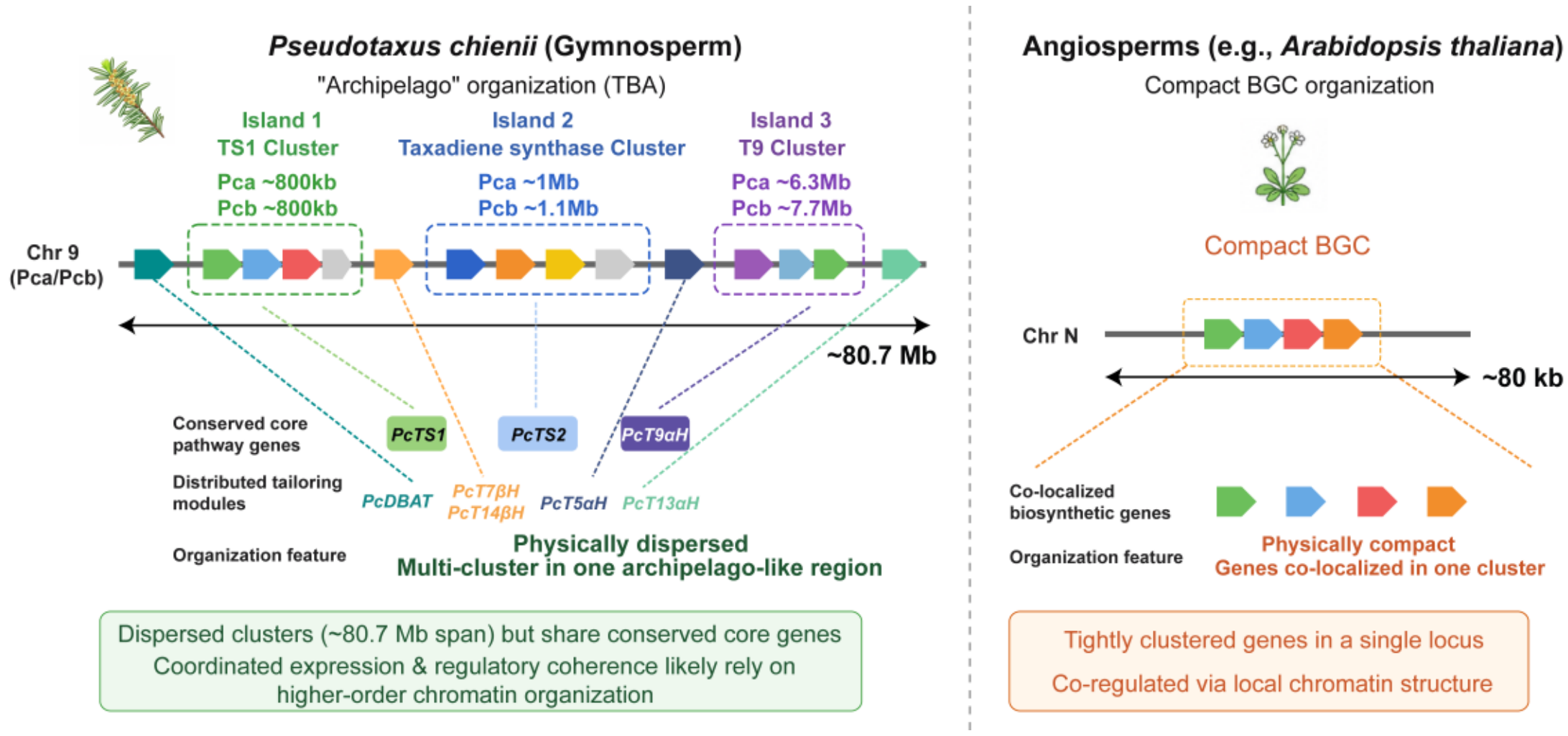

Supplementary Figure 13. Schematic comparison of the "archipelago" organization in *Pseudotaxus chienii* versus compact biosynthetic gene clusters (BGCs) in angiosperms. In *P. chienii* (left), the Taxane Biosynthetic Archipelago (TBA) spans a massive genomic region (~80.7 Mb), featuring physically dispersed gene cluster (TS1 Cluster (TS1C), Taxadiene Synthase Cluster (TSC), and T9 Cluster (T9C)) and scattered core pathway genes (e.g., *PcDBAT*, *PcT5αH*, *PcT13αH*) that likely rely on higher-order chromatin organization for coordinated expression. In contrast, typical angiosperm BGCs (right) are physically compact (e.g., ~80 kb), with co-localized biosynthetic genes tightly clustered in a single locus and co-regulated via their local chromatin structure.

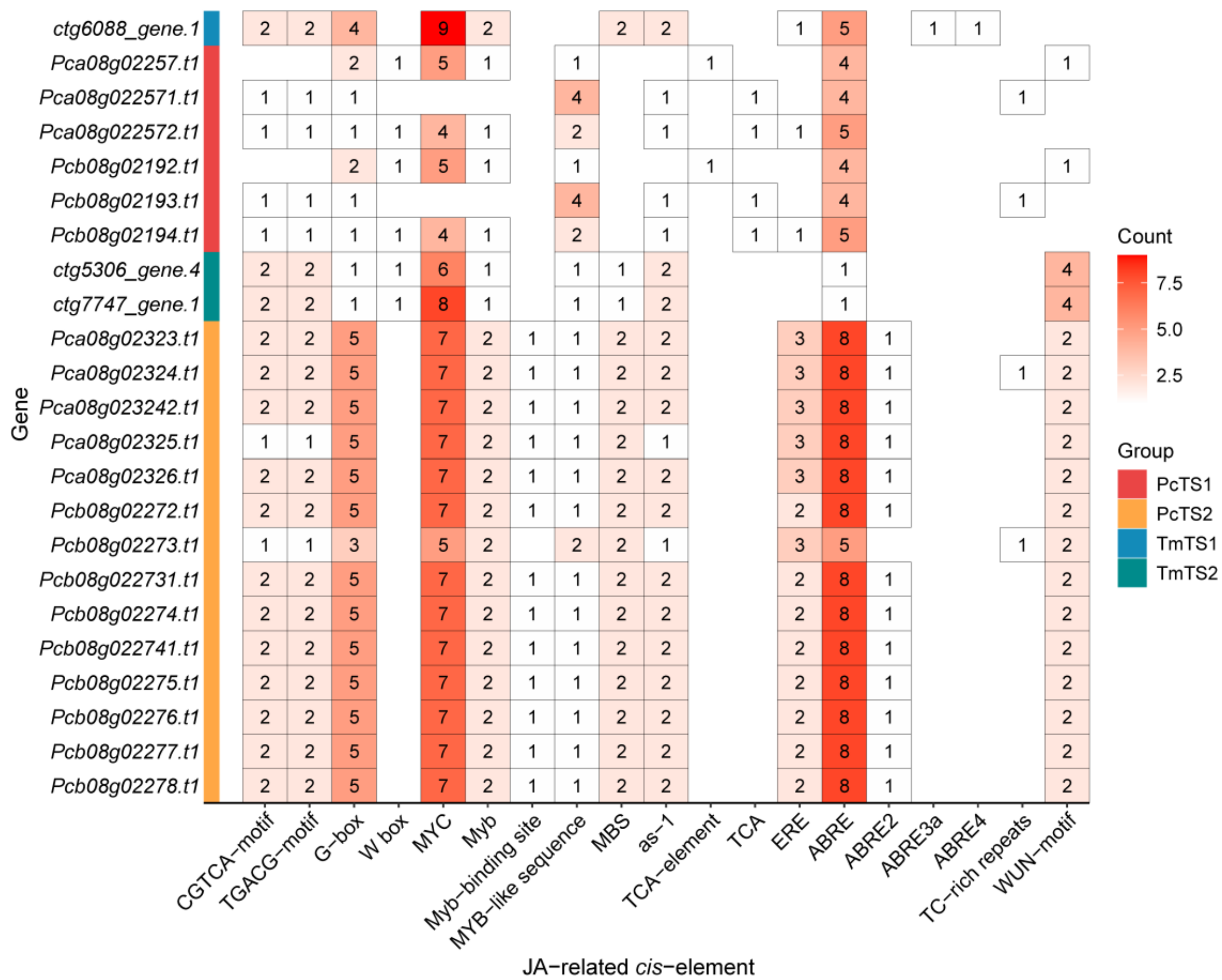

Supplementary Figure 14. Analysis of promoter *cis*-elements across *TS1* and *TS2* from *Pseudotaxus chienii* and *Taxus. mairei*. The figure displays the distribution and counts of various *cis*-regulatory elements within the promoters of *TS1* and *TS2* genes from *P. chienii* and *T. mairei*. Source data are provided as a Source Data file.

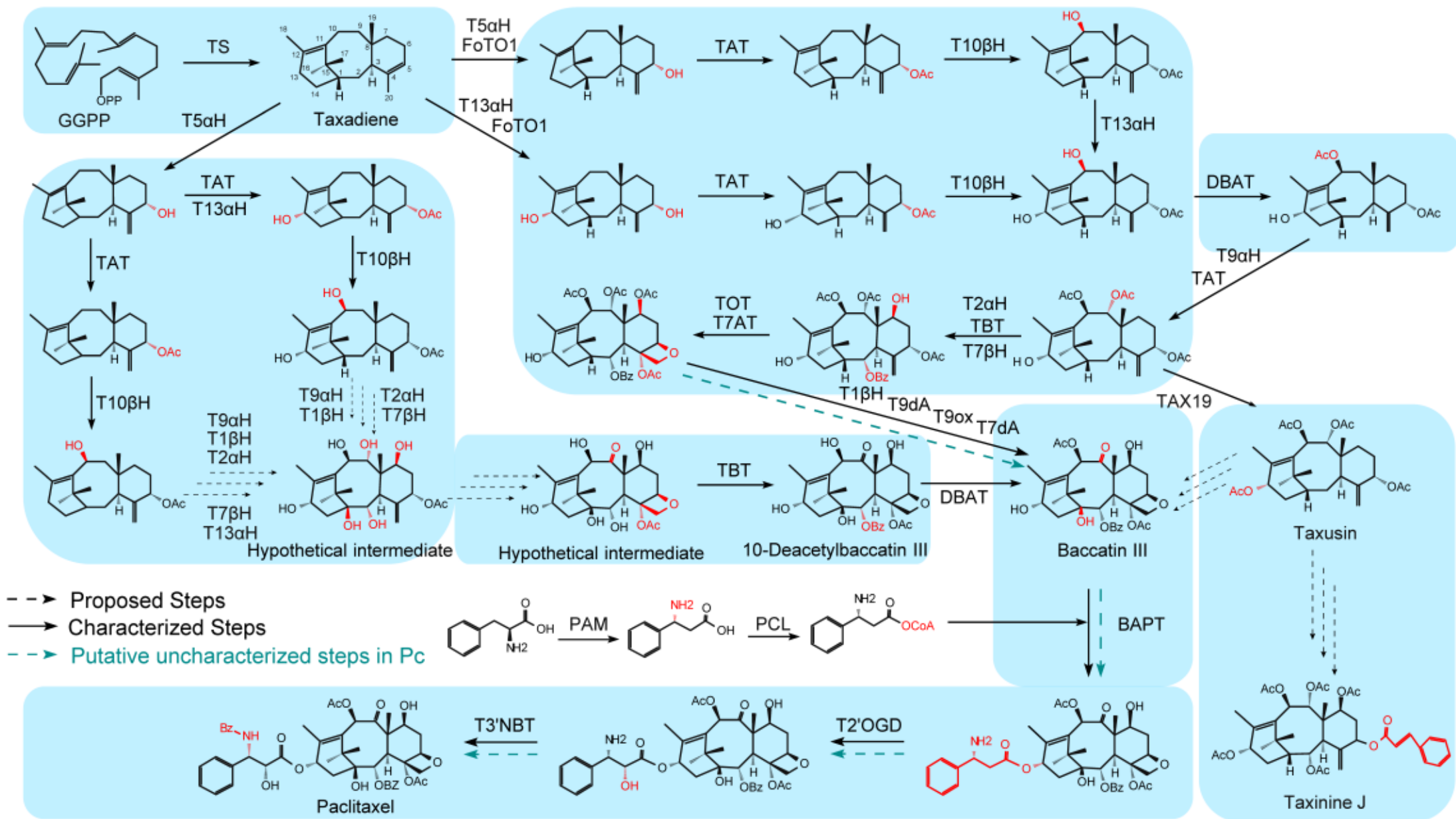

Supplementary Fig. 15. The paclitaxel biosynthetic pathway in *Pseudotaxus chienii*. Schematic representation of the complete paclitaxel biosynthetic pathway from GGPP to paclitaxel in *P. chienii*, with predicted structures of key enzymes and catalytic steps.

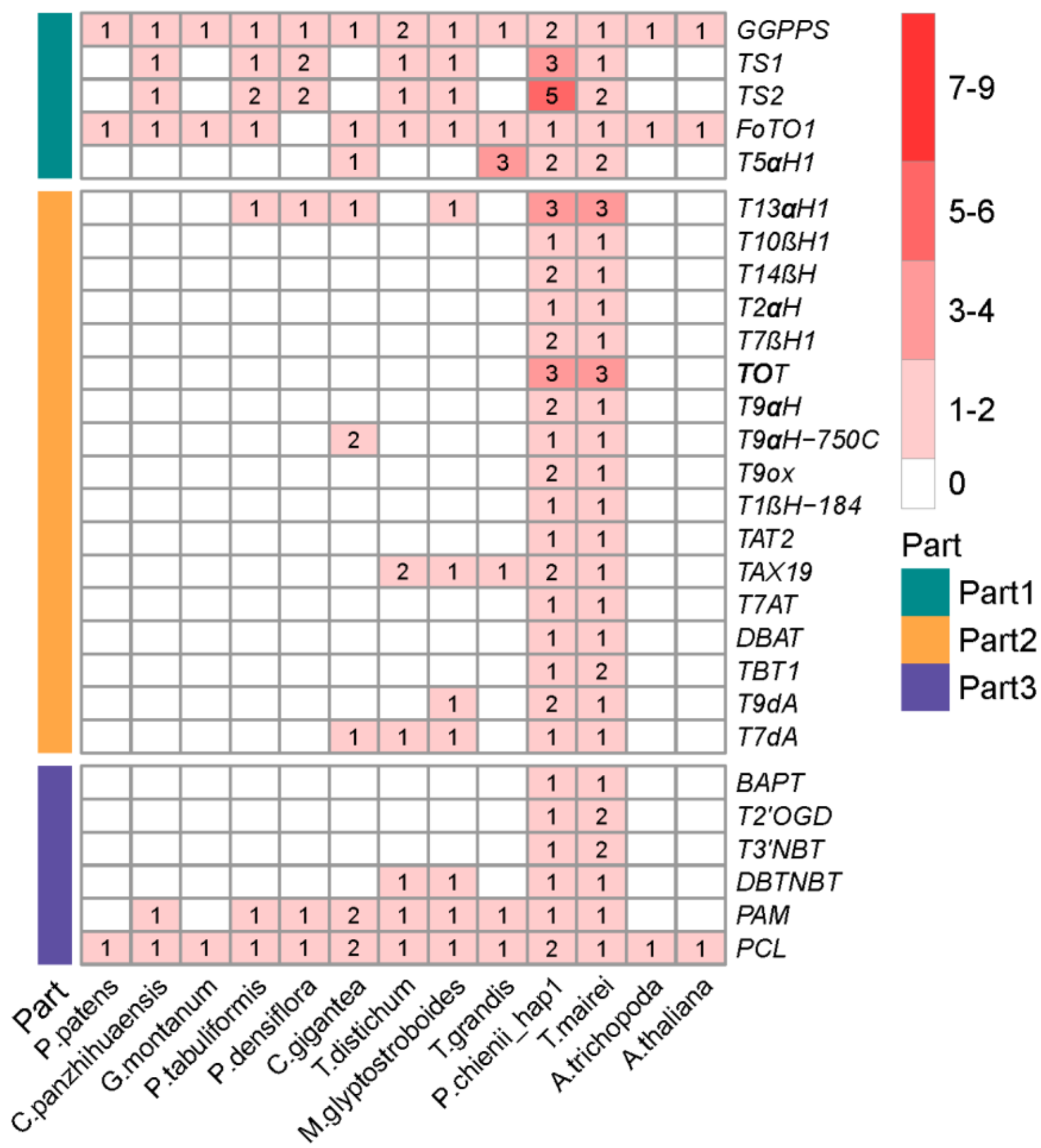

Supplementary Fig. 16. Distribution of orthologous genes involved in the paclitaxel biosynthetic pathway across bryophytes, gymnosperms, and angiosperms. The heatmap illustrates gene copy numbers (0–9, indicated by the color scale). Genes are categorized into three functional modules: part1 (upstream pathway), part2 (midstream pathway), and part3 (downstream pathway). Source data are provided as a Source Data file.

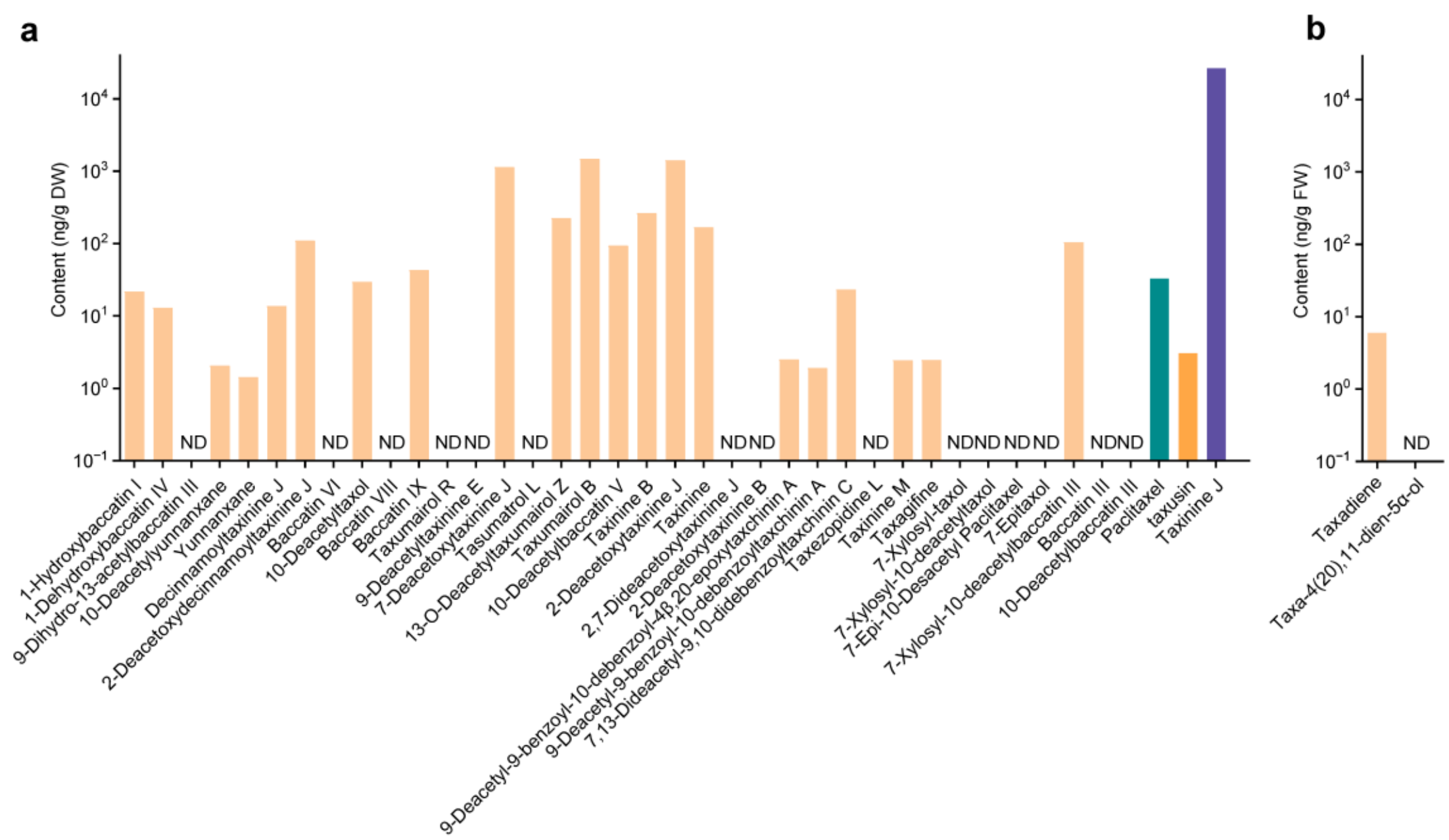

Supplementary Fig. 17. Major taxoids in *Pseudotaxus chienii* leaf. a Content of major taxane expressed as ng/g dry weight (DW). **b** Contents of selected toxoids determined on fresh weight basis (ng/g FW)**.** Metabolites that were undetected in the assay were indicated by “ND” (Not Detected). Source data are provided as a Source Data file.

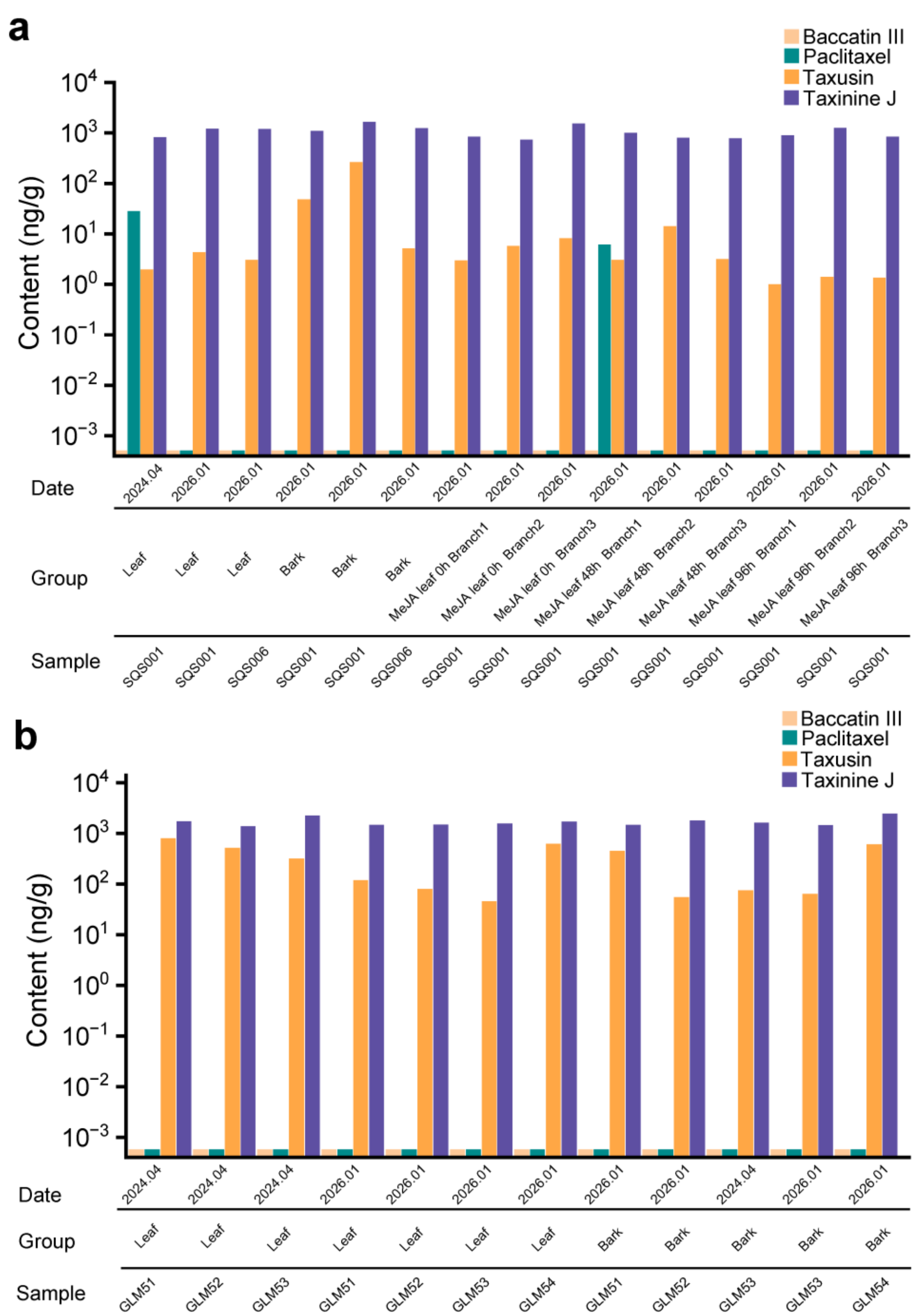

Supplementary Fig. 18. Spatiotemporal targeted quantification of metabolites of four taxanes in *Pseudotaxus chienii* using a 7500 LC-MS/MS platform. a Taxane content (ng/g DW) in samples from the Sanqingshan habitat (SQS001, SQS006), comprising natural leaf and bark tissues (April 2024, January 2026), as well as branch samples (January 2026) treated with MeJA for 0, 48, and 96 hours. **b** Taxane content (ng/g DW) in samples from the Jinggangshan habitat (GLM51 - GLM54), comprising leaf and bark tissues collected at two time points (April 2024 and January 2026). For visualization purposes on the logarithmic scale, metabolites that were undetected were assigned a nominal value of 0.0005 ng/g. Source data are provided as a Source Data file.

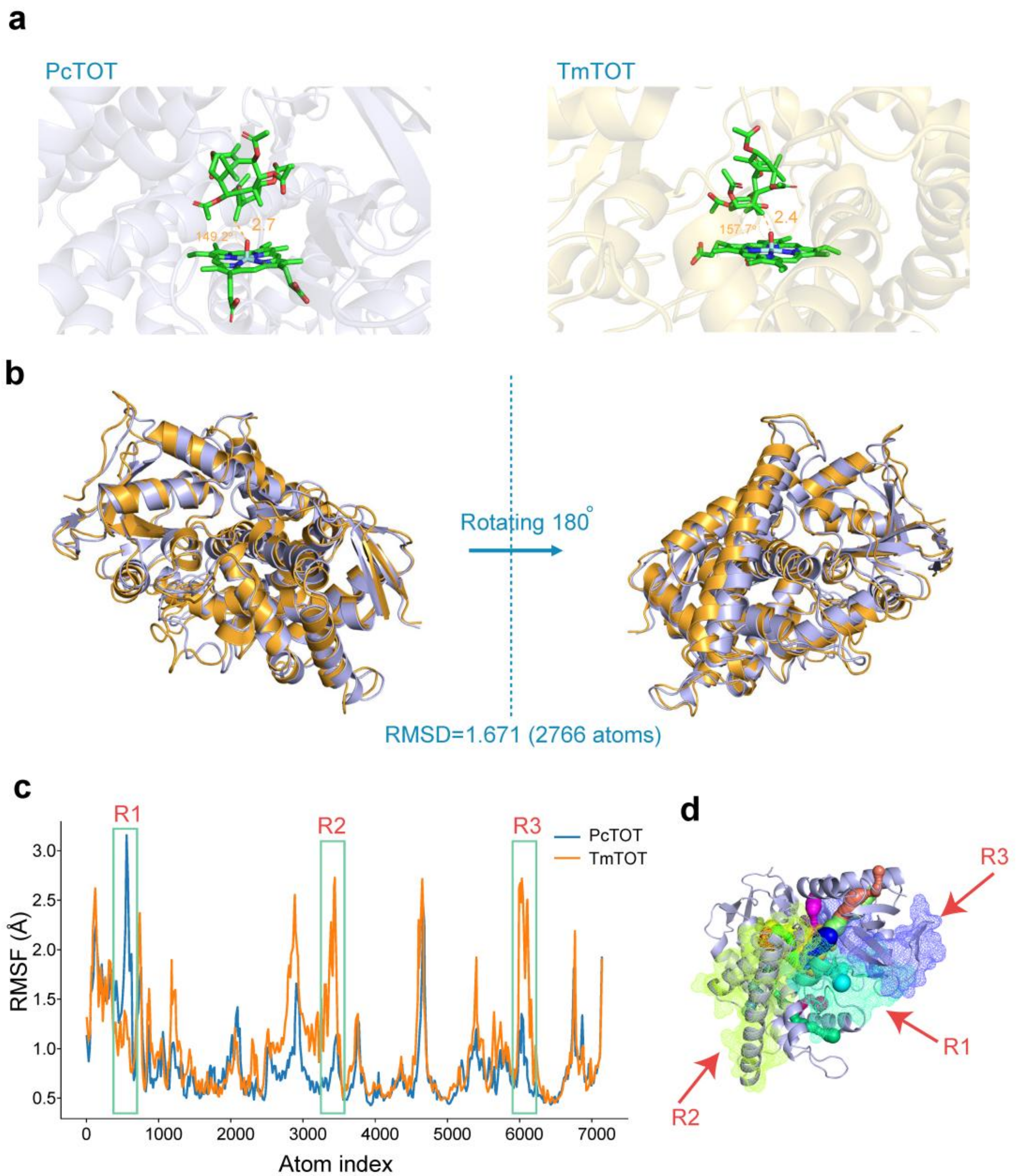

Supplementary Fig. 19. Structural comparison and molecular dynamics simulation of PcTOT and TmTOT. a Active site alignment of PcTOT (left) and TmTOT (right). The taxane substrate (upper sticks) is positioned directly above the catalytic heme co-factor (lower sticks). Orange dashed lines indicate the distance (Å) and reaction angle (degrees) between the heme-activated oxygen and the target atom of the substrate. **b** Structural superposition of the overall models of PcTOT (light blus) and TmTOT (orange) shown from two perspectives rotated by 180° (root-mean-square deviation (RMSD) = 1.671 Å across 2766 atoms). **c** Root-mean-square fluctuation (RMSF) profiles calculated from 200-ns molecular dynamics simulations. The blue and orange lines represent the atomic fluctuations of PcTOT and TmTOT, respectively. The green boxes highlight three specific structural flexibility regions, designated R1, R2, and R3. **d** Three-dimensional spatial mapping of the R1, R2, and R3 regions (indicated by red arrows and meshes) on the 3D enzyme structure, illustrating their physical locations relative to the substrate-binding pocket and access channel.

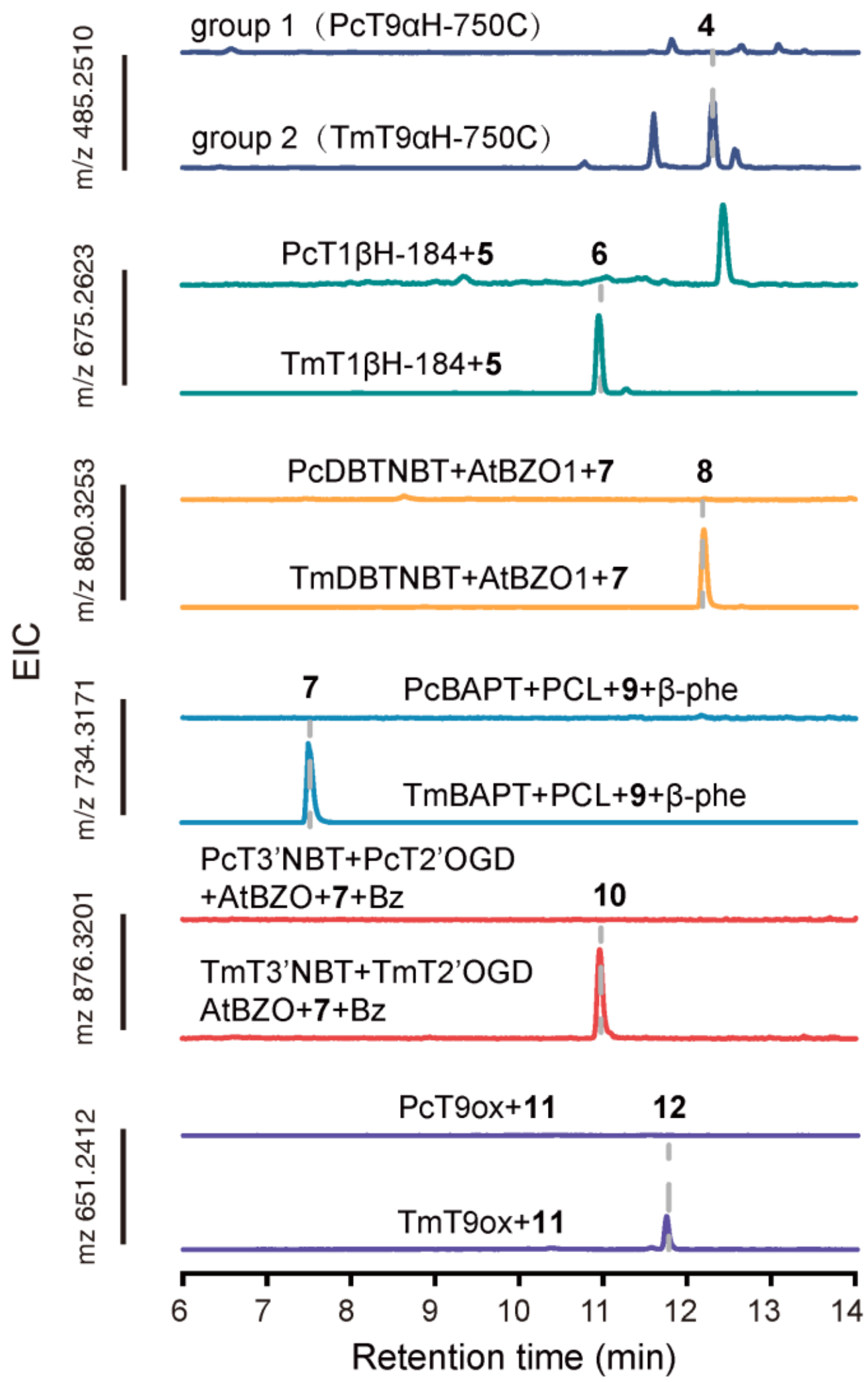

Supplementary Fig. 20. Functional characterization of candidate paclitaxel biosynthesis genes from *Pseudotaxus chienii.* *In vivo* enzymatic activities of candidate genes from *P. chienii* (group 1) were assayed using a transient expression system in tobacco leaves. The corresponding orthologous genes from *T. mairei* (group 2) were used as positive controls in all assays. Extracted ion chromatograms (EICs) illustrate the production of target metabolites across different functional validation assays: *De novo* synthesis of 13a-deacetyltaxusin (**4**) by co-expressing *PcT9aH-750C* with upstream biosynthesis genes. Production of baccatin IV (**6**) in leaves expressing *PcT1βH-184* following the infiltration of 1-dehydroxybaccatin IV (**5**). Production of 2' -deoxypaclitaxel (**8**) in leaves co-expressing *PcDBTNBT* and *AtBZ01* with the infiltration of 3'-debenzoyl-2 -deoxypaclitaxel (**7**). Synthesis of **7** in leaves co-expressing *PcBAPT* and *PCL* (a phenylalanine-CoA ligase with an A312G mutation from *Penicillium chrysogenum*) supplemented with baccatin I (**9**) and B-phenylalanine (β-phe). Production of paclitaxel (**10**) in leaves expressing *PcT3'NBT*, *PcT2'OGD*, and *AtBZO1* with the infiltration of **7** and benzoic acid (Bz). Production of 13-acetylbaccatin III (**12**) in leaves expressing *PcT9ox* following the infiltration of 9-dihydro-13-acetylbaccatin III (**11**).
