# Supplementary material for: Archipelago architecture and evolution of paclitaxel biosynthesis revealed by the Pseudotaxus chienii haplotype-resolved genome
Source: Nat Commun. 2026 Aug 26;17:10189. doi: 10.1038/s41467-026-77115-w (PMC13612588; doi:10.1038/s41467-026-77115-w)
Supplement: Supplementary file 3 [file 41467_2026_77115_MOESM2_ESM.pdf]

nature portfolio

Peer Review File

Archipelago architecture and evolution of paclitaxel biosynthesis revealed by the Pseudotaxus chienii haplotype-resolved genome

Corresponding Author: Professor Jianbin Yan

This file contains all reviewer reports in order by version, followed by all author rebuttals in order by version.

Version 0:

Reviewer comments:

Reviewer #1

(Remarks to the Author)
The manuscript by Wang et al entitled Archipelago architecture and evolution of paclitaxel biosynthesis revealed by the Pseudotaxus chienii haplotype-resolved genome describes an interesting study of a sister group species of Taxus. The genome analysis performed is of very high quality allowing some intriguing hypothesis to be made. I was highly impressed with this part of the manuscript. That said the more biochemical aspect of the paper is currently not to the same standard and will likely be a difficult read for anyone but taxol researchers. I would suggest the following (i) a model of the paclitaxel biosynthetic pathway of this species and (ii) a considerable expansion of the discussion of this pathway. It is for example particularly surprising given the last authors previous work that baccattin III is not discussed further. Additionally given that the authors state that this species is under threat it would be important to outline strategies by which the taxene biosynthesis of this species can be preserved.

Beyond these comments whilst easy to understand the manuscript contains a lot of non-standard English and could do with being corrected by a native speaker or professional company.

Reviewer #2

(Remarks to the Author)
Wang and colleagues have assembled a haplotype-resolved genome of the rare and endangered conifer Pseudotaxus chienii and performed an integrative analysis combining comparative genomics, transcriptomics, metabolomics, and functional enzymology. The central finding—a structurally conserved yet physically dispersed genomic organization termed the “Taxane Biosynthetic Archipelago” (TBA) for paclitaxel biosynthesis—represents a novel concept that advances our understanding of metabolic gene cluster evolution in plants with large, complex genomes. The genomic resource itself holds considerable value for the conservation and study of Taxaceae. The study tackles a long-standing question in plant specialized metabolism and offers valuable insights into the evolution of taxane biosynthesis and the genomic architecture underlying it. Overall, the study is substantial, well-executed, and of broad relevance to plant genomics, specialized metabolism, and evolutionary biology, and I have only some minor suggestions to further improve the manuscript.

1. The assembly quality is exemplary, yet the section could benefit from comparative context. Authors may explicitly compare P. chienii assembly statistics (contig N50, BUSCO completeness, repeat composition) with other gymnosperm genomes (e.g., Taxus mairei, Ginkgo biloba, Pinus taeda) and recently published P. chienii assembly, to highlight relative improvement or novelty. The repeat composition (>88%) places P. chienii among the most repetitive plant genomes known. Since transposon purging mechanisms differ across species, a brief comparison of TE purging efficiency or methylation systems with T. mairei would enhance mechanistic understanding. In addition, Pseudotaxus chienii is a dioecious species. The study notes that LTR expansion in P. chienii occurred earlier (~21.59 MYA) than in T. mairei. The authors may discuss whether this timing correlates with any specific paleoclimatic events or speciation bottlenecks that might have triggered such transposon activity or the relaxation of purifying selection against repetitive elements.

2. The sex of the sequenced individual (accession SQS001) is not indicated in the manuscript. This information is relevant for interpreting the assembled genome (e.g., regarding potential sex chromosomes) and the metabolite profiles. Please specify the sex in the “Plant materials and sample collection” section. If the sex is unknown, this should be stated explicitly and noted as a consideration for future studies.

3. The concept of the “Taxane Biosynthetic Archipelago” (TBA) is central to the manuscript, yet its defining criteria are introduced retrospectively, based on sequential analyses (proximity followed by co-expression). A clearer operational definition should be provided earlier—for instance, in the final paragraph of the Introduction or when the term is first used in the Results. For example: “Here, we define the TBA as a genomic region containing multiple physically dispersed yet syntenically conserved gene groups and core enzymes, whose functional coordination is supported by transcriptional co-expression analysis.” In addition, its definition remains descriptive. Quantitative analysis, for instance, correlation heatmaps or chromatin interaction data (Hi-C) linking the clusters, could substantiate their functional integration.

4. In the study, the description of metabolic flux could be strengthened. While transcription and metabolite data were analyzed separately, true “multi-omic integration” would entail correlating gene expression, metabolite abundance, and enzyme function statistically (e.g., network or flux correlation analyses). In discussion, the current text states: “Despite retaining a complete paclitaxel biosynthetic pathway, P. chienii accumulates trace amounts of paclitaxel and taxusin. In contrast, the substantially elevated levels of taxinine J…” Consider rephrasing to better highlight the metabolic branching point, for instance: “Our findings indicate that although P. chienii retains the full genetic complement for paclitaxel biosynthesis, this potential is not fully realized at the level of end-product accumulation. Instead, metabolic flux appears to be preferentially channeled toward taxinine J, as evidenced by…”

5. The difference in TS2 and other copy numbers between haplotypes is interesting. The author may consider testing whether these differences correlate with distinct metabolite profiles or stress responses in vivo, rather than only inferring from JA induction patterns.

6. In the “ Phylogenomic insights into the origin and diversification of the paclitaxel Pathway ” section, I suggest the authors discuss the paclitaxel biosynthetic pathway within a broader phylogenetic context. Elaborating on when the pathway achieved functional integrity during evolution and identifying potential secondary loss events in related lineages would significantly enhance the depth of the evolutionary narrative.

7. In Supplementary Fig. 13, the preliminary metabolic profiling of P. chienii bark does not show Baccatin III, a critical precursor in paclitaxel biosynthesis. The authors should clarify whether this compound was below the limit of detection (LOD) or absent. Similarly, 10-deacetylbaccatin Ⅲ, another essential intermediate, is missing from the figure; please specify if it was monitored. Furthermore, key upstream intermediates such as GGPP, taxadiene, and taxa-4(20),11-dien-5α-ol are not presented. The authors should provide these data or justify their exclusion. Lastly, the figure title should be revised to "Major taxoids in P. chienii bark" to more accurately represent the specific class of compounds displayed.

8. The authors attribute reduced paclitaxel accumulation to deleterious sequence changes and functional divergence, but direct evidence for such mutations (e.g., active-site substitutions) is missing. Protein modeling and mutation mapping could substantiate the argument.

9. The manuscript convincingly demonstrates low paclitaxel but high taxinine J accumulation in P. chienii and suggests that prioritizing taxinine J is an evolved defense strategy. Is there any ecological or bioactivity evidence suggesting that taxinine J is more effective than paclitaxel against the specific pathogens or herbivores found in P. chienii's habitat? Without this context, the "adaptive" argument remains speculative.

10. The methods section states that experiments were performed on leaves 6, 7, and 8 of the same N. benthamiana plant as three biological replicates, each consisting of four leaf discs. However, the Results section lacks data representing these replicates (e.g., error bars, SD/SEM, or individual data points). Please provide the underlying data for these replicates or incorporate statistical error bars into the figures to show experimental consistency.

11. The conclusion regarding the low paclitaxel accumulation relies heavily on the reduced catalytic efficiency of P. chienii TOT. Quantitative kinetic parameters (Km, Kcat, Vmax, or relative rate ratios) would strengthen the functional argument beyond qualitative rate comparisons. Furthermore, only five downstream genes were tested. Is PcTOT the only rate-limiting enzyme identified? A more comprehensive analysis of the downstream pathway enzymes(e.g.,T1βH, BAPT, T3'NBT, DBTNBT) would strengthen the claim that metabolic flux is shifted specifically towards taxinine J due to this specific bottleneck.

12. The authors propose that the coordinated function of the dispersed TBA depends on "higher-order spatial chromatin organization." While this is a logical hypothesis, the Discussion does not explicitly state if Hi-C interaction data supports physical interactions between these distant loci (~80.7 Mb apart). If such data is available, it should be highlighted to substantiate the claim that regulatory coherence transcends physical continuity. If not, the language should be tempered to reflect that this is a hypothesis rather than a confirmed mechanism.

Other Minor problems:

Line 83, 101, 103, 106, 127, 130：
Supplementary Tables 2, 3, 4, and 5 were not found in the supplemental files.

Line 85：
Please specify "contig N50" instead of just "N50" to avoid ambiguity and ensure clarity regarding the genome assembly quality metrics.

Lines 134-137:
Indicate how divergence times were estimated (e.g., molecular clock model, calibration fossils).

Lines 170-172:
There is a discrepancy in the number of candidate biosynthetic gene clusters (BGCs) reported between the text, figures, and tables. Please cross-check these numbers and ensure consistent labeling and reporting throughout the manuscript. Clarify whether identical BGC identification criteria (plantiSMASH parameters) were used for both haplotypes.

Line 171:
Supplementary Figure 10 lacks panel labels (e.g., a, b). Please add these labels to the figure and ensure they correspond with the descriptions in the figure legend.

Lines 188-190:
Please include a schematic diagram comparing the “archipelago” organization in P. chienii vs. compact BGCs in angiosperms for intuitive interpretation.

Lines 197-198：
The authors mention a translocation event but do not refer to a specific figure. Please cite the relevant figure(s) that provide visual or analytical evidence for this genomic structural variation.

Lines 233-234:
Clarify whether taxinine J detection relied on internal reference standards or MS fragmentation matching only.

Line 240：
Replace “trace leaves” with “trace amount in leaves.”

Lines 250-252, 436-437:
To ensure experimental reproducibility, please provide a comprehensive list of all primer sequences used for gene cloning. It is recommended to include this information in supplementary file.

Line 264:
The phrase "inefficient removal of ancient repetitive elements" is ambiguous. Briefly mentioning the mechanism (e.g., lack of unequal recombination) in the context of P. chienii specifically would make the argument clearer for non-genomics specialists.

Line 352:
Please include the formal citation for the GCEv1.0.0.

Line 440:
Please provide appropriate citations for the Agrobacterium-mediated transformation protocol to support the methodology described in this section.

Improve figure labeling for readability (e.g., Fig. 2b/c).

In Figure 3A, the meaning of the different colored blocks following each gene is unclear.

Define abbreviations (e.g., T5αH, TS, T13αH) upon first mention in the text.

Reviewer #3

(Remarks to the Author)
Reviewer Comments on NCOMMS-25-103630 Archipelago architecture and evolution of paclitaxel biosynthesis revealed by the Pseudotaxus chienii haplotype-resolved genome

The study presents a high-quality haplotype-resolved genome assembly of Pseudotaxus chienii, representing a substantial technical and conceptual advance for investigating the evolution of the taxanes biosynthetic pathway. The haplotype-level resolution is particularly valuable for interrogating genome organization, structural variation, and metabolic gene clustering, especially given the high degree of heterozygosity observed in P. chienii.

In this context, the conclusions of the present study contrast with those of the recent work by Wang M. et al. (2025) (https://www.nature.com/articles/s41467-025-67849-4), which reported that P. chienii harbors only a partial taxane biosynthetic pathway, terminating prior to the biosynthesis of 10-deacetylbaccatin III, based on a chromosome-scale genome

assembly. By leveraging a haplotype-resolved genome assembly, the authors of the present study were able to identify and experimentally validate some orthologs of the paclitaxel biosynthetic pathway.

Notably, the high level of haplotypic divergence implies substantial intraspecific genetic variability, which, in interaction with environmental factors, may contribute to the existence of distinct chemotypes within P. chienii. This highlights the importance of considering genotype × environment interactions when interpreting metabolic diversity and pathway completeness in highly heterozygous species.

These results underscore the critical importance of high-resolution, haplotype-aware genome assemblies when studying complex metabolic pathways in species with high heterozygosity, where collapsed or consensus assemblies may obscure essential genes or entire pathway modules.

However, several points require clarification or improvement to strengthen the conclusions and enhance the clarity of the manuscript.

1. Genome assembly and karyotype inference
The assembly reports 12 pseudochromosomes. It is unclear whether this number reflects the expected karyotype of the species or results from assembly or scaffolding choices. Please clarify the cytogenetic basis (if known) and explicitly state whether the pseudochromosome number corresponds to the actual chromosome number.

2. Genome size estimation by flow cytometry
Supplementary Figure 2 (a, b, c) is not sufficiently explicit to support the conclusions regarding genome size estimation.
• First, the peaks corresponding to T. marei and P. chienii should be clearly identified and labeled in panels 2a, 2b, and 2c.
• Second, I strongly suggest summarizing the flow cytometry data in a table, specifying for each species:
• species name
• C-value (1C genome size)
• PI-A peak position
• estimated nuclear DNA mass

Such a table would clearly support your results.

3. Visualization of clustering results
In Supplementary Figure 10, clusters are difficult to distinguish because the colors used are too similar. A revised color palette with higher contrast would substantially improve readability.

4. Visualization of co-expression modules
Readability, legend and explanations to interpret Supplementary Figure 11 should be improve for reader not familiar with this type of analysis. Does these colors correspond to those mentioned in Supplementary Data 3 ? Does the complete gene set have been clustered or only metabolic related genes ? In addition, more explanation should be provided on the source of the data (108 RNA seq libraries) and plant materials° ? Is this a reanalysis of previously obtained data (reference 68) ? Do the plant materials originate from the same source as in this manuscript ?

5. Missing overview of the metabolic pathway
A schematic representation of the complete taxane biosynthetic pathway is missing. To facilitate comprehension, I recommend adding a figure summarizing:
• the main metabolites
• the corresponding enzymatic steps
• the identified genes from P. chienii and/or T. marei

This could be included as supplementary material, for instance by expanding Supplementary Figure 12B.

A complete pathway would not only help for comprehension but also to confirm, or not, that the complete gene set for paclitaxel biosynthesis is present in P. chienii. For instance, homolog for T9ox (a 2-oxoglutarate dependent dioxygenase, McClune et al, 2025) was apparently not reported in this manuscript. If this is correct, P. chienii does not have a complete biosynthetic pathway for paclitaxel.
In addition, a number of putative homologs with lower sequence identity were not functionally tested, including T9αH-750C (68.7% identity), T1βH-184C (68.2%), BAPT (67.8%), T3'NBT (73%) and DBTNBT (71.9%). To argue a complete paclitaxel pathway, it would be important to test these as well.

6. Archipelago genomic structure and pathway organization
The reported archipelago genomic structure is highly interesting, but the figures do not clearly convey its biological implications.

It would be important to clarify whether the physical genomic organization of the archipelago follows the biosynthetic logic of the taxane biosynthetic pathway and if so, to explicitly illustrate the metabolic pathway through the lens of the archipelago structure.
If no direct correspondence exists, please discuss whether different pathway modules are nevertheless correlated with specific archipelago regions.

7. Clarification of paclitaxel detection and quantification in P. chienii
The presence of paclitaxel in Pseudotaxus was mentioned to be controversial in the literature. The authors claim to detect and quantify paclitaxel in both leaves and bark of Pseudotaxus chienii in this report. Figure 3 report between 10-5 and 10-6 µg/mg of paclitaxel in P. chienii bark, so in the 1 to 10 ng per g range. These levels detected are extremely low and the methodology however not fully reported (sMRM protocol), and thus hard to evaluate that methods are adequate for trace detection. Detecting trace level of paclitaxel in complex matrix may be highly challenging. Minimum quality control would require matrix spike with similar level of paclitaxel d5 (the amount reported in M&M is much higher), standard addition (ie 2 or 3 spikes eg +1 , +2 + 5 ng/g equivalent) into the "same" extract to show the signal scales linearly and the paclitaxel/paclitaxel d5 ratio behaves properly. The sMRM method should relies on two MRM transitions (quantifier and qualifier) and both must be present at the same RT, with a stable peak shape. Qualifier/quantifier ratio should match standards (typical acceptance window : + - 20 to 30% relative). A typical chromatogram for sMRM detection in bark extract should be presented to assess the background/signal ratio. LOD and LOQ reported as well. In addition, note that carryover (sticking to glassware, metal, lines) is a recognized analytical risk in trace analysis by LC-MS/MS. Best practice in trace analysis is to demonstrate carryover removal as part of validation.
The error bars for taxane levels in Figure 3 are very small, likely corresponding to a technical repeat. Biological repeats should be performed.

8 Clarification of preliminary metabolic profiling versus targeted LC–MS
The distinction between preliminary metabolic profiling and targeted LC–MS analysis in P. chienii is not clearly defined. Only LC–MS experiments are described in the Materials and Methods section, making it difficult to understand what is meant by "preliminary metabolic profiling." Please clarify whether this term refers to an initial metabolite annotation or identification step using computational prediction tools (e.g. SIRIUS or related software), or to a distinct experimental approach that is not fully described.

9. Discussion in relation to Wang et al. (2025) and implications for chemical diversity
Given that the conclusions of the present study differ from those reported by Wang et al. (2025) regarding the completeness of the taxane biosynthetic pathway in P. chienii, the authors should explicitly discuss and contextualize these discrepancies. In particular, the discussion should address how differences in genome assembly strategy and resolution (chromosome-scale versus haplotype-resolved assemblies) may have led to divergent biological interpretations. In addition, the authors are encouraged to consider how haplotypic and genetic diversity may contribute to chemical diversity and the existence of distinct chemotypes in P. chienii, potentially shaped by genotype × environment interactions, and to discuss the implications of this variability for taxane biosynthesis and pathway reconstruction.

In this manuscript, a major focus is the question of whether the taxane biosynthetic pathway is complete. Unless this issue can be clearly resolved, the authors may consider redirecting their discussion toward a broader perspective on the evolution of taxanes biosynthesis.

additional comments :
line 82 : supplementary table 2 not provided
line 169 : supplementary figure 10, not 10a, b ?
figure 2 b and c, the cluster appears to have different names in 2b and 2c. Please clarify.

Reviewing by Pr B.St-Pierre and Dr M. Durand

Reviewer #4

(Remarks to the Author)
I co-reviewed this manuscript with one of the reviewers who provided the listed reports. This is part of the Nature Communications initiative to facilitate training in peer review and to provide appropriate recognition for Early Career Researchers who co-review manuscripts.

Version 1:

Reviewer comments:

Reviewer #1

(Remarks to the Author)
I am completely satsified with the authors revisions. I have no further comments.

Reviewer #2

(Remarks to the Author)
The revision has addressed most of my concerns. I have no more question.

Reviewer #3

(Remarks to the Author)
In the manuscript entitled “Archipelago architecture and evolution of paclitaxel biosynthesis revealed by the Pseudotaxus chienii haplotype-resolved genome”, the authors have provided a revised version that addresses many of the concerns raised during the previous review round.
Overall, most of the questions raised by Reviewers #3 and #4 have been satisfactorily addressed. However, we still have a major concern regarding the conclusion that Pseudotaxus chienii retains the capacity to synthesize paclitaxel.
In my view, the new data provided by the authors further strengthen the alternative interpretation that P. chienii may have largely lost, or no longer possess, a functional paclitaxel biosynthetic pathway. The authors demonstrate that several enzymes corresponding to late steps of the proposed pathway exhibit either markedly reduced catalytic activity (PcTOT, Fig. 3e) or no detectable activity under the tested conditions (T9ox, T9αH-750C, T1βH-184C, BAPT, T3′NBT, and DBTNBT; Supplementary Figure 20). Collectively, these observations suggest that multiple bottlenecks exist in the terminal portion of the pathway and raise questions regarding the feasibility of complete paclitaxel biosynthesis in this species.
Furthermore, the additional metabolite analyses performed on leaves and bark from natural populations revealed no detectable paclitaxel in the vast majority of samples examined. According to Supplementary Figure 18, only two of twenty-seven samples were reported as positive. Given the extensive sampling effort, this result appears more consistent with the absence of a functional pathway than with a pathway that remains operational but is expressed only under specific conditions.
One of the two positive samples deserves particular attention. In this case, branches were treated with methyl jasmonate, and trace amounts of paclitaxel (<10 ng g^{-1}) were detected at the 48 h time point in only one of three sampled branches, whereas paclitaxel was no longer detected after 96 h. This observation is difficult to reconcile with de novo induction of paclitaxel biosynthesis by methyl jasmonate. An alternative explanation that should be considered is the possibility of trace contamination. At concentrations close to analytical detection limits, carryover can originate not only from the analytical instrumentation itself but also from laboratory consumables, extraction equipment, glassware, plasticware, or reagent preparation procedures. This issue is well recognized in trace analytical chemistry, including pesticide residue analysis, where stringent precautions are routinely implemented to prevent false-positive detections. For example, see :
GUIDELINES ON GOOD LABORATORY PRACTICE IN PESTICIDE RESIDUE ANALYSIS
https://www.fao.org/input/download/standards/378/cxg_040e.pdf
https://www.eurl-pesticides.eu/library/docs/allcrl/AqcGuidance_Sanco_2003_10476.pdf

Consequently, I do not believe that the current data provide sufficient evidence to support the conclusion that P. chienii retains “a complete paclitaxel biosynthetic pathway with a highly conditional residual capacity dependent on genotype-by-environment interactions.” Rather, the combined biochemical and metabolomic evidence appears more consistent with the alternative hypothesis that P. chienii lacks a functional capacity for paclitaxel biosynthesis, with the rare positive detections requiring additional verification.
I therefore recommend that the authors revise their discussion and conclusions to more explicitly acknowledge this alternative interpretation. At a minimum, the manuscript should present the absence of functional paclitaxel biosynthesis as a plausible explanation supported by the available data, rather than favoring residual biosynthetic capacity as the primary conclusion.

Specific comments :
Improve resolution of supplementary figure 15

Version 2:

Reviewer comments:

Reviewer #3

(Remarks to the Author)
The authors have adequately addressed most of my comments, and I have no further remarks.

Open Access This Peer Review File is licensed under a Creative Commons Attribution 4.0 International License, which permits use, sharing, adaptation, distribution and reproduction in any medium or format, as long as you give appropriate credit to the original author(s) and the source, provide a link to the Creative Commons license, and indicate if changes were made.
In cases where reviewers are anonymous, credit should be given to 'Anonymous Referee' and the source.
The images or other third party material in this Peer Review File are included in the article's Creative Commons license, unless indicated otherwise in a credit line to the material. If material is not included in the article's Creative Commons license and your intended use is not permitted by statutory regulation or exceeds the permitted use, you will need to obtain permission directly from the copyright holder.
To view a copy of this license, visit https://creativecommons.org/licenses/by/4.0/

Response to REVIEWER COMMENTS:

Reviewer #1:

Summary

The manuscript by Wang et al entitled Archipelago architecture and evolution of paclitaxel biosynthesis revealed by the *Pseudotaxus chienii* haplotype-resolved genome describes an interesting study of a sister group species of *Taxus*. The genome analysis performed is of very high quality allowing some intriguing hypothesis to be made. I was highly impressed with this part of the manuscript. That said the more biochemical aspect of the paper is currently not to the same standard and will likely be a difficult read for anyone but taxol researchers.

Response: Thank you for your recognition of our work. We are very grateful for your professional review work, constructive comments, and valuable suggestions on our manuscript.

R1Q1: I would suggest the following **(i)** a model of the paclitaxel biosynthetic pathway of this species.

Response: We appreciate this comment. To clearly illustrate the enzymes and intermediate metabolites involved in paclitaxel biosynthesis in *Pseudotaxus chienii*, we have drawn a comprehensive paclitaxel biosynthetic pathway diagram, with predicted structures of key enzymes and catalytic steps. Detailed data are presented in Supplementary Fig. 15.

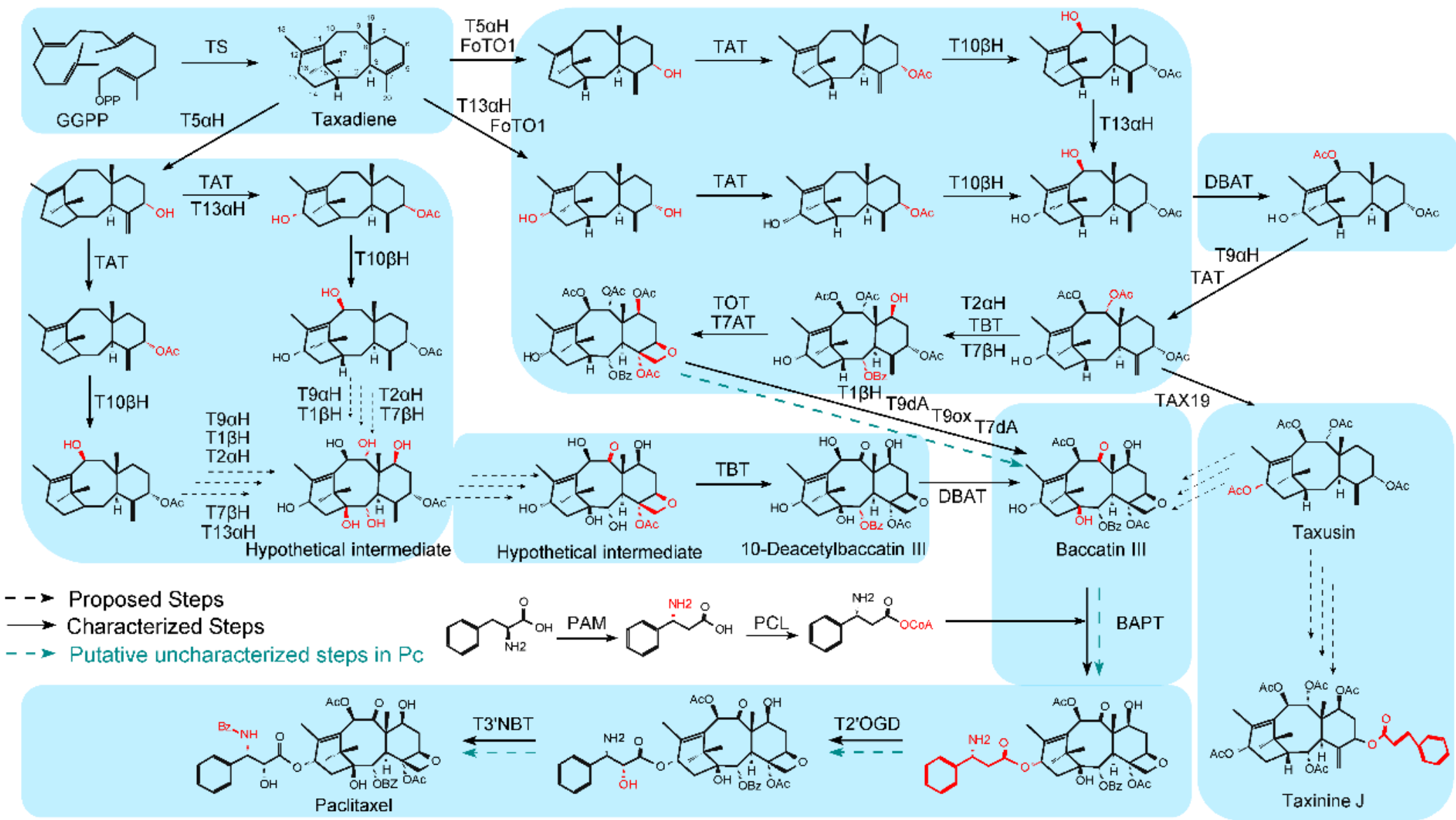

Supplementary Fig. 15 | The paclitaxel biosynthetic pathway in *Pseudotaxus chienii*. Schematic representation of the complete paclitaxel biosynthetic pathway from GGPP to paclitaxel in *P. chienii*, with predicted structures of key enzymes and catalytic steps.

R1Q2: and **(ii)** a considerable expansion of the discussion of this pathway. It is for example particularly surprising given the last authors previous work that baccattin III is not discussed further.

Response: We are grateful for your insightful comments and the chance to refine the manuscript for tighter integration with our experimental findings. We repeated or supplemented taxane metabolite quantification experiments on *P. chienii* samples collected across different seasons, geographic regions, and treatments. These analyses consistently failed to detect the key intermediate baccatin III in any sample (or found it below the detection limit), while paclitaxel was only traceably detected in specific samples. This suggests that paclitaxel biosynthesis is not a constitutive, species-wide characteristic of *P. chienii*. Although the relevant pathway genes exhibit clear transcriptional activity, paclitaxel production appears to be a highly restricted, conditionally dependent residual metabolic capacity. The dominant accumulation of

taxinine J, coupled with the consistent absence of baccatin III across all natural populations and sampling conditions, indicates a significant functional bottleneck in the downstream portion of the canonical taxane pathway. Further experiments revealed that the downstream functional modules of the paclitaxel biosynthetic pathway in *P. chienii* showed no detectable enzymatic activity in heterologous expression systems, strongly supporting the presence of severe functional attenuation at the terminal steps of the pathway. We have incorporated results and discussions regarding the evolution and divergence of the paclitaxel biosynthetic pathway in *P. chienii*, the functional degeneration of its enzymes, and metabolite detection outcomes, including the consistent non-detection of baccatin III:

Page 8-9, lines 322-343, "Given the trace accumulation of paclitaxel in the initial sample, we aimed to systematically determine whether this pattern was a consistent pattern across different populations of the species or a phenomenon limited to a specific region. We performed a comprehensive spatiotemporally targeted taxoid analysis on 2 distinct sampling batches from 4 independent *P. chienii* trees. The samples were taken from two geographically isolated habitats with distinct latitudes and longitudes: Sanqingshan (samples SQS001, SQS006) and Jinggangshan (samples GLM51 - GLM54) (Supplementary Fig. 18a, b). To assess temporal variation, we collected leaf and bark samples at two time points: spring 2024 (April) and winter 2026 (January), yielding a total of 12 samples (representing available tissues across seasons). All 17 spatiotemporal samples were analyzed in parallel using a high-sensitivity 7500 LC-MS/MS system, while 2024 archived samples were previously cross-validated on a 5500 LC-MS/MS platform.

Across all tissues, sampling sites and time points detected, taxinine J exhibited the highest abundance among the four targeted taxanes (Supplementary Fig. 18a, b). In marked contrast, baccatin Ⅲ was undetectable in all assayed samples. Trace levels of paclitaxel were exclusively found in leaves of a single *P. chienii* individual (SQS001) collected from Sanqingshan on April, 2024. Interestingly, in the Jinggangshan populations, although taxinine J maintained the highest abundance, taxusin also

accumulated to a high level in selective samples, with its concentration peaking at approximately half that of taxinine J. Furthermore, MeJA elicitation of *P. chienii* leaves yielded trace paclitaxel in only one out of three independent branches after 48h treatment, whereas no paclitaxel was captured in the 96h treatment group (Supplementary Fig. 18a), and no paclitaxel was detected any natural samples collected in 2026. Collectively, these sporadic detections suggest the retained paclitaxel pathway remains latent, strictly gated by specific G × E interactions."

Page 9-10, lines 360-377, "To preliminarily elucidate the reasons for the attenuated function of PcTOT compared to TmTOT, structural modeling and molecular docking analyses were first performed. The results revealed a slightly more relaxed catalytic conformation in PcTOT (2.7 Å/149.2°) compared to the highly optimized geometric parameters observed in TmTOT (2.4 Å/157.7°) (Supplementary Fig. 19a**)**, despite their overall structural similarity with a root-mean-square deviation (RMSD) of 1.67 Å across 2,766 backbone atoms (Supplementary Fig. 19b**)**. Intriguingly, molecular dynamics simulations revealed distinct, region-specific flexibility patterns across these structural regions. Specifically, PcTOT exhibited a pronounced conformational mobility within the R1 segment, yet displayed a distinctly repressed flexibility, evidenced by lower calculated residue-level root-mean-square fluctuation (RMSF) within R2 and R3 regions (Supplementary Fig. 19c**)**. Based on CAVER analysis, these differentially rigidified regions were spatially mapped onto the residues lining the substrate access channels leading to the buried catalytic pocket (Supplementary Fig. 19d**)**. Taken together, these complementary static and dynamic analyses suggest that the altered flexibility and subsequent channel gating dynamics likely contribute to the divergent catalytic behaviors of the two enzymes. Moreover, despite genomic retention and high sequence conservation with *Taxus* orthologs, heterologous expression of six core downstream candidates, including *T9ox* and *BAPT*, in *Nicotiana benthamiana* failed to yield detectable pathway products, in sharp contrast to active *T. mairei* counterparts (Supplementary Fig. 20**)**."

Page 11-12, lines 440-485, "Resolving the historical debate surrounding paclitaxel biosynthesis in *P. chienii* requires addressing not only contradictory metabolic evidence, but also divergent genomic interpretations reported in recent studies. For instance, Wang et al. (2025)[16] proposed that the pathway terminates prior to 10-deacetylbaccatin III, a conclusion likely constrained by the limitations of consensus chromosome-scale genome assemblies. In contrast, our haplotype-resolved genome demonstrates that the complete genetic architecture underlying paclitaxel biosynthetic pathway, including the core TBA and other dispersed essential genes, remains structurally intact in *P. chienii*. These results further highlighted the critical importance of haplotype-aware genome assemblies for accurately reconstructing complex specialized metabolic pathways in highly heterozygous plant genomes. However, the observed discrepancies between this genomic retention and the restricted biochemical output suggest a dynamic evolutionary trajectory rather than a contradiction. While our phylogenomic analysis indicates that the complete pathway was step-wise assembled and achieved functional integrity in the common ancestor of *Taxus* and *Pseudotaxus*, this ancient structural retention does not equate to modern functional execution. Following its divergence from *Taxus*, the terminal pathway in the *P. chienii* lineage appears to have undergone significant evolutionary functional degradation. Notably, the paclitaxel content in *P. chienii* leaves is over four orders of magnitude lower than that typically observed in T. mairei. Crucially, our findings reveal that the capacity for paclitaxel biosynthesis outside the *Taxus* genus cannot be simply reduced to a binary question of gene presence or absence. Rather, it represents a clear case of evolutionary functional degeneration. Although the ancestral archipelago architecture has been faithfully retained and the complete set of orthologous pathway genes displays low Ka/Ks ratios indicative of sustained purifying selection[35] and has been robustly preserved to cope with extensive genome expansion, such structural conservation does not necessarily guarantee full functional integrity. Indeed, paclitaxel could only be detected in rare instances: specifically, in a single natural sample (SQS001) collected in April 2024 (unambiguously confirmed by MS/MS spectra; Fig. 3f), together with only one positive case observed among all independently MeJA-induced branches in 2026

(Supplementary Fig. 18). These results suggest that the complete paclitaxel biosynthetic capacity is still retained in *P. chienii*, but exists in a latent rather than constitutively active state. Rather than an experimental artifact, this low induction reproducibility underscores that complete biosynthesis is an ultra-rare, stochastic event tightly gated by intricate G × E interactions. Consistent with this interpretation, the functional assays showed that the terminal steps of the pathway, such as PcTOT, have undergone substantial evolutionary degradation. Consequently, this highly conditional and rare activation elegantly may explain the historical controversy regarding the inconsistent detection of paclitaxel in *P. chienii* across previous studies[14-16].

To systematically investigate whether this functional deficiency extends across the downstream pathway, we characterized two core late-stage functional modules. The first module contains key oxygenases involved in skeleton modification, including T9ox (65.3% sequence identity to the *Taxus* ortholog), T9αH-750C (68.7% identity), and taxane 1β-hydroxylase (T1βH-184) (68.2% identity). The second module includes terminal acyltransferases responsible for side-chain formation and attachment, namely baccatin III:3-amino-3-phenylpropanoyl transferase (BAPT) (67.8% identity), taxoid-3'-N-benzoyltransferase (T3'NBT) (73.0% identity), and 3'-N-debenzoyl-2'-deoxypaclitaxel-N-benzoyl transferase (DBTNBT) (71.9% identity). Despite genomic retention and considerable sequence conservation, none of the genes from either downstream module produced detectable pathway products in Nicotiana benthamiana, whereas the functional competence of the corresponding T. mairei orthologs remained intact (Supplementary Fig. 20)."

R1Q3: Additionally given that the authors state that this species is under threat it would be important to outline strategies by which the taxene biosynthesis of this species can be preserved.

Response: Thank you for your suggestion. We have added findings regarding how genetic variation in *P. chienii* interacts with environmental changes to shape distinct chemotypes, emphasized the significance of these findings for conservation strategies,

and proposed specific measures for protecting this endangered species. The Discussion section has been revised accordingly:

Page 11, lines 431-439, "…Furthermore, such underlying genetic variation interacts with environmental changes to shape distinct chemotypes, underscoring the need to account for G × E interactions in exploring metabolic diversification[50]. Given the endangered status of *P. chienii*, these findings carry important implications for the development of more effective conservation strategies to protect its distinctive taxane biosynthetic capability. Rather than focusing exclusively on increasing population sizes, in situ conservation efforts should prioritize the protection of heterogeneous microhabitats so as to preserve G × E interactions that drive chemical diversity. At the same time, ex situ germplasm collections can utilize the haplotype-resolved markers generated in this study to capture and safeguard the full range of chemotypes along with the species' underlying genetic architecture."

R1Q4: Beyond these comments whilst easy to understand the manuscript contains a lot of non-standard English and could do with being corrected by a native speaker or professional company.

Response: We have revised the entire manuscript to ensure its style and grammar meet the standards of the journal according to your suggestions.

Reviewer #2:

Summary

Wang and colleagues have assembled a haplotype-resolved genome of the rare and endangered conifer *Pseudotaxus chienii* and performed an integrative analysis combining comparative genomics, transcriptomics, metabolomics, and functional enzymology. The central finding—a structurally conserved yet physically dispersed genomic organization termed the "Taxane Biosynthetic Archipelago" (TBA) for paclitaxel biosynthesis—represents a novel concept that advances our understanding of metabolic gene cluster evolution in plants with large, complex genomes. The genomic resource itself holds considerable value for the conservation and study of *Taxaceae*. The study tackles a long-standing question in plant specialized metabolism and offers valuable insights into the evolution of taxane biosynthesis and the genomic architecture underlying it. Overall, the study is substantial, well-executed, and of broad relevance to plant genomics, specialized metabolism, and evolutionary biology, and I have only some minor suggestions to further improve the manuscript.

Response: We appreciate your recognition of the innovation and significance of this research.

R2Q1: The assembly quality is exemplary, yet the section could benefit from comparative context. Authors may explicitly compare *P. chienii* assembly statistics (contig N50, BUSCO completeness, repeat composition) with other gymnosperm genomes (e.g., *Taxus mairei*, Ginkgo biloba, Pinus taeda) and recently published *P. chienii* assembly, to highlight relative improvement or novelty. The repeat composition (>88%) places *P. chienii* among the most repetitive plant genomes known. Since transposon purging mechanisms differ across species, a brief comparison of TE purging efficiency or methylation systems with T. mairei would enhance mechanistic understanding. In addition, *Pseudotaxus chienii* is a dioecious species. The study notes

that LTR expansion in *P. chienii* occurred earlier (~21.59 MYA) than in T. mairei. The authors may discuss whether this timing correlates with any specific paleoclimatic events or speciation bottlenecks that might have triggered such transposon activity or the relaxation of purifying selection against repetitive elements.

Response: We sincerely thank you for constructive suggestions, which have helped us better contextualize our genomic findings and improve our manuscript.

R2Q1 (1): The assembly quality is exemplary, yet the section could benefit from comparative context. Authors may explicitly compare *P. chienii* assembly statistics (contig N50, BUSCO completeness, repeat composition) with other gymnosperm genomes (e.g., *Taxus mairei*, Ginkgo biloba, Pinus taeda) and recently published *P. chienii* assembly, to highlight relative improvement or novelty.

Response: We have provided a comprehensive comparison of contig N50, BUSCO completeness, and repeat content between our assembly, representative gymnosperms (e.g., *Taxus mairei*, *Ginkgo biloba*, *Pinus taeda*), and the recently published *P. chienii* consensus genome (Wang et al.,2025) in a new supplementary file (Supplementary Data 5). We have revised the Discussion section to explicitly address these metrics and explain our assembly performance. We emphasize that while consensus genomes usually have high linear contiguity, our haplotype-resolved assembly has a clear advantage in resolving the allelic and structural complexity of this highly heterozygous genome (which contains >88%repetitive sequences). In addition, our assembly achieves 95% BUSCO completeness, one of the highest values reported for published gymnosperm genomes so far, which shows its high quality and completeness (Detailed description is presented in manuscript (**lines 380-387**).

Page 10, lines 380-387, "We compared its contig N50, BUSCO completeness of protein model and repeat content with representative gymnosperms and a recently released *P. chienii* genome assembly, confirming the high reliability of our genomic dataset

(Supplementary Data 5)[7,16,24,25,38-42]. Although consensus assembly may have better linear contiguity, our haplotype-resolved assembly more effectively resolves the structural complexity of this highly heterozygous genome with over 88% repetitive sequences. Our protein annotation achieves a 95% BUSCO completeness, one of the highest among reported gymnosperms, and this high-resolution assembly facilitates the accurate reconstruction of intact metabolic pathways."

R2Q1 (2): The repeat composition (>88%) places *P. chienii* among the most repetitive plant genomes known. Since transposon purging mechanisms differ across species, a brief comparison of TE purging efficiency or methylation systems with T. mairei would enhance mechanistic understanding. In addition, *Pseudotaxus chienii* is a dioecious species. The study notes that LTR expansion in *P. chienii* occurred earlier (~21.59 MYA) than in T. mairei. The authors may discuss whether this timing correlates with any specific paleoclimatic events or speciation bottlenecks that might have triggered such transposon activity or the relaxation of purifying selection against repetitive elements.

Response: Paleoclimate and LTR expansion: We have added a new discussion linking the ~ 21.59MYA LTR retrotransposon burst to major Early Miocene climatic changes, particularly the strengthening of the Asian monsoon (lines **390-395**). We hypothesize that these severe environmental fluctuations caused demographic bottlenecks in *P. chienii*. Such population declines would have relaxed purifying selection, allowing for the extensive retention of repetitive elements during this period.

TE Purging Mechanisms: To further clarify the massive repeat expansion, we explicitly state that the larger genome size of *P. chienii* is mainly attributed to the inefficient removal of ancient TEs-specifically resulting from a lack of unequal recombination-rather than a higher transposition frequency (**lines 395-397**).

Methylation Systems: We fully agree that comparing TE purging efficiency and methylation systems with *T. mairei* is crucial for understanding TE silencing. While targeted methylation data (e.g., WGBS) are not currently available for our *P. chienii*

samples to perform this direct comparison, we have clearly highlighted comparative epigenetics as an important and necessary direction for future research.

Page 10, lines 390-397, "This expansion period aligns closely with major Early Miocene climatic shifts, most notably the strengthening of the Asian monsoon[43]. Though current substitution rates based molecular dating cannot confirm direct causality, severe environmental fluctuations likely triggered demographic bottlenecks[44]. Such population declines would subsequently reduce the efficacy of purifying selection, permitting the extensive retention of repetitive elements[45]. The larger genome of *P. chienii* was primarily attributed to the inefficient removal of ancient repetitive elements resulting from a lack of unequal recombination, rather than more frequent transposition[46,47]."

R2Q2: The sex of the sequenced individual (accession SQS001) is not indicated in the manuscript. This information is relevant for interpreting the assembled genome (e.g., regarding potential sex chromosomes) and the metabolite profiles. Please specify the sex in the "Plant materials and sample collection" section. If the sex is unknown, this should be stated explicitly and noted as a consideration for future studies.

Response: Thank you for your question. In this study, the *P. chienii* sample used for genome sequencing was collected from a male tree. This information has been added to the "Plant materials and sample collection" section.

Page 13, lines 524-526, "Fresh leaves and bark tissues were collected from a mature wild individual of *Pseudotaxus chienii* (accession SQS001, Male) located in Sanqingshan National Park (28.90087°N, 118.08942°E), Jiangxi Province, China."

R2Q3: The concept of the "Taxane Biosynthetic Archipelago" (TBA) is central to the manuscript, yet its defining criteria are introduced retrospectively, based on sequential analyses (proximity followed by co-expression). A clearer operational definition should

be provided earlier—for instance, in the final paragraph of the Introduction or when the term is first used in the Results. For example: "Here, we define the TBA as a genomic region containing multiple physically dispersed yet syntenically conserved gene groups and core enzymes, whose functional coordination is supported by transcriptional co-expression analysis." In addition, its definition remains descriptive. Quantitative analysis, for instance, correlation heatmaps or chromatin interaction data (Hi-C) linking the clusters, could substantiate their functional integration.

Response: Thanks for your excellent suggestion. We agree that the conceptual framework and operational definition of the "Taxane Biosynthetic Archipelago" (TBA) should be introduced more explicitly and supported by quantitative evidence demonstrating functional integration.

In response, we have substantially revised the manuscript in both the Introduction and Results sections. First, we now provide a clear operational definition of the TBA at its initial introduction in the Introduction section (**lines 79–84**). Specifically, we added the following statement: "Here, we define the TBA as a genomic architecture composed of multiple physically dispersed yet syntenically conserved biosynthetic gene groups and core pathway enzymes, whose functional coordination is supported by transcriptional co-expression and chromatin interaction analyses." This definition is now introduced prior to the detailed Results description to clarify the conceptual framework from the outset.

Second, to address the reviewer's concern that the original definition was primarily descriptive, we incorporated additional quantitative analyses supporting the functional integration of the TBA. Specifically, we integrated: i) large-scale co-expression analyses using an integrated dataset of 128 *P. chienii* transcriptomes, ii) Pearson correlation analyses among core pathway genes, and iii) Hi-C chromatin architecture analyses.

These new analyses demonstrate that the three physically separated BGCs (TS1C, TSC, and T9C), together with dispersed core paclitaxel biosynthetic genes, exhibit highly coordinated transcriptional behavior despite extensive genomic separation. Most

core metabolic genes showed strong co-expression relationships (Pearson correlation coefficients generally ⩾ 0.9). In addition, Hi-C analyses revealed that the TBA resides within the transcriptionally active A compartment and that the individual clusters are organized into distinct yet functionally coordinated TAD/sub-TAD structures. Importantly, these results support the central conclusion that functional coherence within the TBA is maintained through shared regulatory coordination and active chromatin occupancy rather than strict physical contiguity. Accordingly, we revised the relevant Results and Discussion sections to emphasize that the TBA represents a regulatory and evolutionary organizational mode distinct from canonical compact biosynthetic gene clusters. These additions substantially strengthen both the conceptual clarity and mechanistic support for the TBA model.

Page 3, lines 79-84, “Leveraging this multi-omics framework, we uncovered previously unrecognized genomic architecture underlying paclitaxel biosynthesis, which we designate the "Taxane Biosynthetic Archipelago (TBA)". Here, we define the TBA as a genomic architecture composed of multiple physically dispersed yet syntenically conserved biosynthetic gene groups and core pathway enzymes, whose functional coordination is supported by transcriptional co expression and chromatin interaction analyses.”

Page 6, lines 223-237, “To quantitatively validate the functional integration of this dispersed genomic archipelago, we combined chromatin architecture profiles derived from Hi-C analysis (using the Pca haplotype as a representative model) with Pearson correlation analyses of a comprehensive 128-sample transcriptomic dataset. Hi-C profiles revealed that the entire TBA region is embedded within the transcriptionally active A compartment[27,28], providing a globally favorable environment for gene expression. Notably, the three gene clusters are spatially segregated into distinct Topologically Associating Domains (TADs)[29,30], which further subdivide into localized sub-TADs (Supplementary Fig. 12a). This hierarchical division closely aligns with functional boundaries; for example, the TAD encompassing TS1C exclusively harbors

core metabolic genes, effectively demarcating the functional domain by excluding a non-metabolic gene at its left border. Although separated into independent TADs, metabolic genes from the three clusters and these dispersed core enzymes form a highly coordinated co-expression network, with the majority of exhibiting Pearson correlation coefficients (r) ≥ 0.9 (Supplementary Fig. 12b). Collectively, these findings indicate that the functional integrity of the TBA is determined by regulatory coordination and active chromatin occupancy, rather than strictly by linear physical proximity."

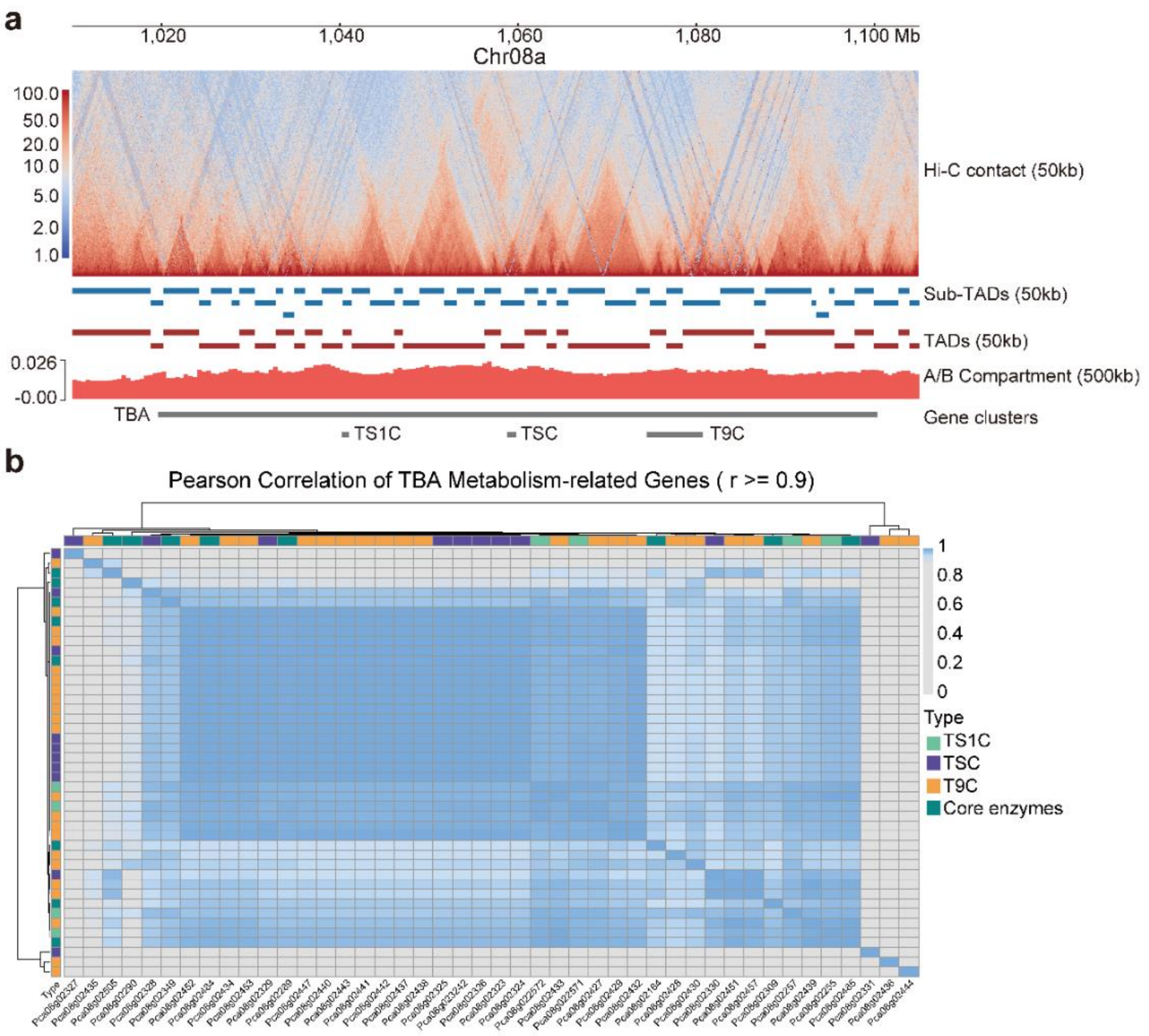

Supplementary Figure 12 | Chromatin structure and co-expression network of metabolism-related genes in the TBA region of Pca. a Hi-C interaction map and chromatin features in the TBA region. Each track (from top to bottom) shows the Hi-C contact frequency, sub-TADs, TADs, A/B compartments, TBA, and the genomic location of the three gene clusters (TS1 Cluster (TS1C), Taxadiene Synthase Cluster (TSC), and T9 Cluster (T9C)). **b** Pearson correlation heatmap of metabolism-related genes in TBA. Based on a transcriptome dataset containing 128 samples, these genes

collectively form a highly coordinated co-expression network, with the vast majority of gene pairs having a Pearson correlation coefficient ($r \geqslant 0.9$).

R2Q4: In the study, the description of metabolic flux could be strengthened. While transcription and metabolite data were analyzed separately, true "multi-omic integration" would entail correlating gene expression, metabolite abundance, and enzyme function statistically (e.g., network or flux correlation analyses). In discussion, the current text states: "Despite retaining a complete paclitaxel biosynthetic pathway, *P. chienii* accumulates trace amounts of paclitaxel and taxusin. In contrast, the substantially elevated levels of taxinine J…" Consider rephrasing to better highlight the metabolic branching point, for instance: "Our findings indicate that although *P. chienii* retains the full genetic complement for paclitaxel biosynthesis, this potential is not fully realized at the level of end-product accumulation. Instead, metabolic flux appears to be preferentially channeled toward taxinine J, as evidenced by…"

Response: We thank you for this valuable suggestion regarding the interpretation and presentation of metabolic flux within the paclitaxel biosynthetic pathway. We agree that the original version did not sufficiently emphasize the relationship between gene expression, metabolite accumulation, and enzymatic functionality within an integrated metabolic framework. In response, we substantially revised the relevant Results and Discussion sections to strengthen the multi-omics interpretation of pathway attenuation and metabolic flux redirection in *P. chienii*.

First, we fully agree with your excellent phrasing suggestion to better reflect the concept of preferential metabolic channeling. Specifically, the original statement has been revised to emphasize the disconnect between genomic retention and end-product realization, as well as the likely redistribution of metabolic flux. The revised text in the Discussion **(lines 494-502)** now states: "Our findings indicate that although *P. chienii* retains the complete genetic architecture for paclitaxel biosynthesis, this potential is not fully realized at the level of end-product accumulation. Instead, metabolic flux appears to be preferentially channeled toward alternative branches, particularly taxinine J. This

metabolic rewiring is evidenced by the marked accumulation of taxinine J, along with discernible levels of taxusin in specific spatiotemporal samples, and the consistent absence of downstream intermediates like baccatin Ill across sampled wild populations. Far from indicating a complete evolutionary loss, this specific taxane accumulation profile provides a biochemical basis for the restriction of complete paclitaxel biosynthesis to an ultra-rare, conditionally dependent trace even."

In addition, we further expanded the interpretation throughout the revised manuscript to emphasize that the observed metabolic phenotype is not explained by gene loss, but rather by functional attenuation at the downstream enzymatic level. This conclusion is supported by the combined evidence from: i) transcriptional activity of pathway-associated genes; ii) targeted metabolite profiling across tissues, seasons, geographic populations, and MeJA treatments, and iii) heterologous enzymatic assays demonstrating markedly reduced or undetectable activities for multiple downstream enzymes. Importantly, we now explicitly discuss that the downstream terminal pathway exhibits severe functional degradation, resulting in restricted conversion toward canonical paclitaxel intermediates and a concomitant redirection of metabolic flux toward taxinine J accumulation. The consistent absence of baccatin III across all spatiotemporal samples, together with the reduced catalytic efficiency of *PcTOT* and the lack of detectable activity in downstream functional modules (e.g., BAPT, DBTNBT), provides biochemical support for this metabolic rewiring model.

Although direct quantitative metabolic flux modeling is currently technically challenging in this slow-growing non-model gymnosperm species, we believe the revised integration of transcriptomic, metabolomic, chromatin, and enzymatic evidence substantially strengthens the multi-omics interpretation of pathway evolution and functional attenuation in *P. chienii*.

R2Q5: The difference in TS2 and other copy numbers between haplotypes is interesting. The author may consider testing whether these differences correlate with distinct metabolite profiles or stress responses in vivo, rather than only inferring from JA induction patterns.

Response: We thank you for this insightful suggestion. The haplotype-specific expansion of *TS2* and related genes raises the important question of whether structural variation contributes to differential metabolic outputs or stress responsiveness in vivo. In the revised manuscript, we addressed this point by expanding both the Results and Discussion sections to more carefully interpret the biological implications of haplotype-specific copy number variation. Specifically, we incorporated additional analyses of promoter *cis*-regulatory elements, which revealed that *TS2* promoters are strongly enriched for jasmonate-responsive motifs (including CGTCA-motifs, TGACG-motifs, and MYC binding sites), whereas these elements are largely absent from *TS1* promoters. Combined with previous reports that *TS2* is preferentially induced under stress conditions, these results support the hypothesis that the expanded *TS2* copies may contribute to inducible defense-related taxane biosynthesis. In addition, we substantially expanded our spatiotemporal metabolite analyses by examining multiple natural populations, different sampling seasons, distinct tissues, and JA-treated samples. These experiments revealed considerable variability in trace paclitaxel accumulation across individuals and environmental conditions. Notably, trace paclitaxel was detected only in a single individual collected at a specific time point, while most other samples lacked detectable accumulation even after MeJA treatment. These observations further support the idea that taxane metabolism in *P. chienii* is strongly influenced by genotype-by-environment (G × E) interactions.

However, directly assigning metabolite phenotypes to individual haplotypes remains technically challenging in this highly heterozygous diploid gymnosperm system, because metabolite accumulation reflects the combined output of both haplotypes within the same individual. Therefore, although our current data support a potential adaptive role for *TS2* copy expansion in stress-responsive metabolism, establishing a direct causal relationship between specific haplotypes and metabolic phenotypes will require future allele-specific functional analyses and population-scale phased metabolomics.

To clarify these limitations and avoid overinterpretation, we revised the manuscript to frame the *TS2* expansion primarily as evidence of regulatory specialization and adaptive gene dosage potential, rather than as a definitively demonstrated determinant of metabolite output.

Page 7, lines 252-258, "To uncover the regulatory basis of this TS2 expansion, we analyzed promoter cis-elements across both haplotypes of *P. chienii* and T. mairei. A clear regulatory divergence was observed: TS2 promoters are highly enriched in Methyl jasmonate (MeJA)-responsive elements (e.g., CGTCA-motifs, TGACG-motifs, and MYC binding sites)[33,34] (Supplementary Fig. 14), which are largely absent in TS1. This pronounced structural divergence suggests a regulatory specialization: while TS1 likely supports baseline expression, the expanded TS2 genes are transcriptionally primed for stress-induced responses."

Page 11, lines 426-433, "…While determining the direct metabolic consequence of this specific allelic expansion is technically impractical within the blended diploid metabolome, our promoter analyses provide robust alternative evidence for its functional role. These analyses firmly support an adaptive gene dosage strategy[49]: TS2 gene expansion allows *P. chienii* to enhance JA-dependent chemical defense upon stress, effectively balancing defensive capacity and metabolic expenditure. Furthermore, such underlying genetic variation interacts with environmental changes to shape distinct chemotypes, underscoring the need to account for G × E interactions in exploring metabolic diversification[50]."

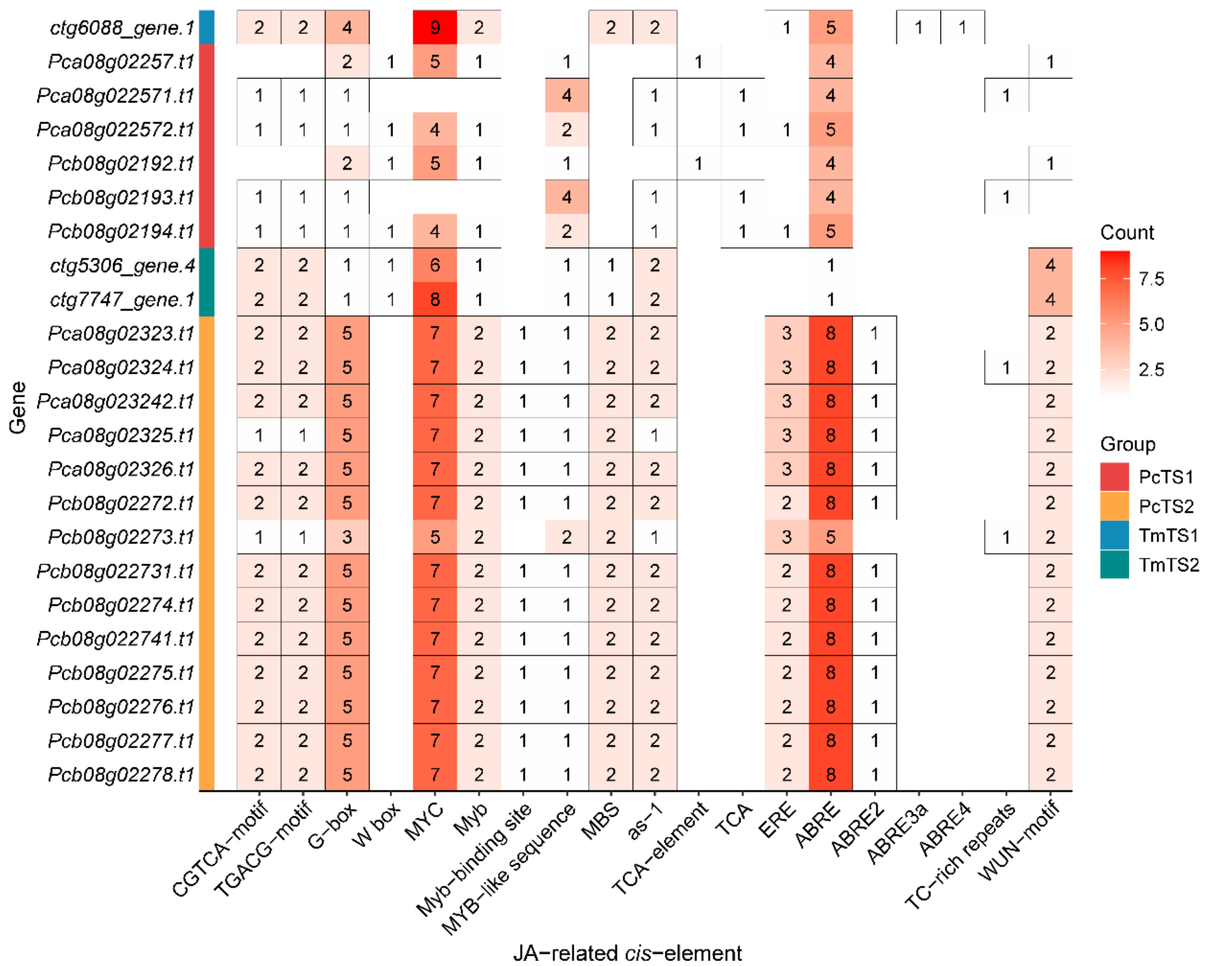

Supplementary Figure 14 | Analysis of promoter *cis*-elements across *TS1* and *TS2* from *Pseudotaxus chienii* and *Taxus. mairei*. The figure displays the distribution and counts of various *cis*-regulatory elements within the promoters of *TS1* and *TS2* genes from *P. chienii* and *T. mairei*.

R2Q6: In the "Phylogenomic insights into the origin and diversification of the paclitaxel Pathway" section, I suggest the authors discuss the paclitaxel biosynthetic pathway within a broader phylogenetic context. Elaborating on when the pathway achieved functional integrity during evolution and identifying potential secondary loss events in related lineages would significantly enhance the depth of the evolutionary narrative.

Response: We thank you for this insightful suggestion. We agree that expanding the phylogenetic context greatly improves the evolutionary narrative of our study.

Accordingly, we have substantially revised the Results and Discussion sections to explicitly address these points.

First, in the Results section (**lines 264-279**): We have expanded the 'Phylogenomic insights...' section to propose a step-wise evolutionary assembly model based on the distribution of 28 key genes. Following your advice, we now specifically highlight the basal *Taxaceae* species *Torrey agrandis*. We clarify that the absence of core backbone genes (e.g., TS) in this species, along with its highly fragmented syntenic region, points to a secondary loss event following lineage divergence, rather than incomplete lineage sorting.

Second, in the Results section (**lines 280-292**): We further clarified the evolutionary timeline by explicitly stating that the "functional integrity" of the complete paclitaxel pathway was exclusively achieved in the most recent common ancestor of *Taxus* and *Pseudotaxus*.

Finally, in the Discussion section (**line 449-455**): To connect this evolutionary background with our main finding (the functional degradation in modern *P.chienii*), we added a new content to the Discussion. We explain that while the pathway achieved functional integrity in their common ancestor, "this ancient structural retention does not equate to modern functional execution." This critical distinction helps separate the historical gain and assembly of the pathway from its recent functional degradation in the *Pseudotaxus* lineage, thereby resolving any potential contradictions.

We believe these updates fully address your concerns and provide a clearer, more comprehensive evolutionary context for our findings.

Page 7, lines 264-279, "…*P. chienii* harbors copies of all currently known paclitaxel biosynthetic genes, indicating that it possesses the genomic potential for paclitaxel production. The upstream precursor biosynthesis genes geranylgeranyl diphosphate synthases (GGPPS), FoTO1, and the phenylpropanoid pathway gene phenylalanoyl-CoA ligase (PCL) are conserved across all surveyed land plant lineages from bryophytes to angiosperms, consistent with their inheritance from a common ancestor of embryophytes.

In contrast, the core taxane backbone-forming terpene synthases TS1 and TS2 are absent in bryophytes, Gnetales and angiosperms, but present in *Pinaceae*, most *Cupressaceae*, and *Taxaceae*. Notably, despite their presence in these broader conifer lineages,TS orthologs were not detected in *Gnetum montanum*, *Cupressus gigantea*, and *T. grandis*. Specifically, the absence of these core genes in the basal *Taxaceae* species *T. grandis*, coupled with its highly fragmented syntenic region (Fig. 2a, Supplementary Fig. 8), is more consistent with secondary loss events following lineage divergence, rather than incomplete lineage sorting. This phylogenetic distribution of TS is potentially driven by gene duplication and subsequent functional specialization within the terpene synthase family, as demonstrated by the functional divergence of TS orthologs in *Taxus wallichiana* and their absence in non-Cupressales species[8].”

Page 7-8, lines 280-292, “A second suite of downstream pathway genes involved various oxygenases, acyltransferases, and side-chain assembly enzyme (*T5αH1*, *T13αH1*, *T9αH-750C*, *T7dA*, *T9dA*, *TAX19*, *DBTNBT* and *PAM*) are present in most conifer lineages (*Pinaceae*, *Taxaceae*, and *Cupressaceae*) and absent from *Gnetales*, bryophytes, and angiosperms. The final set of pathway genes comprising additional likely functional enzymes (e.g. *T10βH1*, *T2αH*, *T7βH1*, *TOT*, *T9αH*, *T7AT*, taxane 2α-O-benzoyltransferase (*TBT1*), *BAPT*) are exclusively detected in the two sequenced *Taxaceae* genomes (*T. mairei* and *P. chienii*), with 1 – 3 copies of each gene and zero copies in all other species surveyed.

This hierarchical distribution of these genes, spanning from ubiquitous precursors, through conifer-enriched modifications, to a suite of oxygenation, acylation and side-chain assembly enzymes, supports a step-wise evolutionary assembly of the pathway. This indicates that the full functional integrity of the complete paclitaxel pathway was exclusively achieved in the most recent common ancestor of *Taxus* and *Pseudotaxus*.”

Page 11-12, lines 449-455, “…However, the observed discrepancies between this genomic retention and the restricted biochemical output suggest a dynamic evolutionary trajectory rather than a contradiction. While our phylogenomic analysis

indicates that the complete pathway was step-wise assembled and achieved functional integrity in the common ancestor of *Taxus* and *Pseudotaxus*, this ancient structural retention does not equate to modern functional execution. Following its divergence from *Taxus*, the terminal pathway in the *P. chienii* lineage appears to have undergone significant evolutionary functional degradation."

R2Q7: In Supplementary Fig. 13, the preliminary metabolic profiling of *P. chienii* bark does not show Baccatin III, a critical precursor in paclitaxel biosynthesis. The authors should clarify whether this compound was below the limit of detection (LOD) or absent. Similarly, 10-deacetylbaccatin III, another essential intermediate, is missing from the figure; please specify if it was monitored. Furthermore, key upstream intermediates such as GGPP, taxadiene, and taxa-4(20),11-dien-5α-ol are not presented. The authors should provide these data or justify their exclusion. Lastly, the figure title should be revised to "Major taxoids in *P. chienii* bark" to more accurately represent the specific class of compounds displayed.

Response: We sincerely thank you for this insightful comment. In the revised manuscript, we substantially expanded the metabolite analysis and clarified the detection status of these key paclitaxel biosynthetic intermediates.

First, regarding the figure itself (now Supplementary Fig. 17), we would like to clarify that we have updated the representative sample from *P. chienii* bark to leaf. As per your valuable suggestion to monitor upstream intermediates (such as taxadiene, and taxa-4(20),11-dien-5α-ol), we had to introduce a newly dedicated GC-MS/MS methodology. This required the complete re-processing and re-extraction of the sample. Because the remaining quantity of our original bark sample was insufficient for this additional extraction, we utilized leaf tissues collected from the exact same batch. This strategy was strictly adopted to avoid any potential biological variations or batch effects that could arise from using samples from different collection time points or individuals. Accordingly, following your constructive recommendation, we have revised the figure

title to “Major taxoids in *Pseudotaxus chienii* leaf” to accurately reflect both the sample type and the specific class of compounds analyzed.

Second, regarding Baccatin III and 10-deacetylbaccatin III: We carefully re-examined their accumulation and explicitly confirm that both compounds were actively monitored in our targeted metabolite analyses. However, they remained consistently undetectable or below the limit of detection (LOD) in all examined *P. chienii* samples under our analytical conditions.

In addition, we further analyzed several upstream intermediates of the taxane pathway. Among them, taxadiene could be detected in *P. chienii*. In contrast, taxa-4(20),11-dien-5α-ol was not detected in our samples. We have now clarified these results in the revised manuscript and supplementary materials. Regarding GGPP, we agree that this metabolite represents an important precursor in taxane biosynthesis. However, due to its highly unstable and low-abundance nature, reliable detection of endogenous GGPP in woody tissues under our experimental conditions remains technically challenging. Finally, following the recommendation, we revised the figure title from a general metabolite description to “Major taxoids in *P. chienii* leaf” to more accurately reflect the specific class of compounds analyzed.

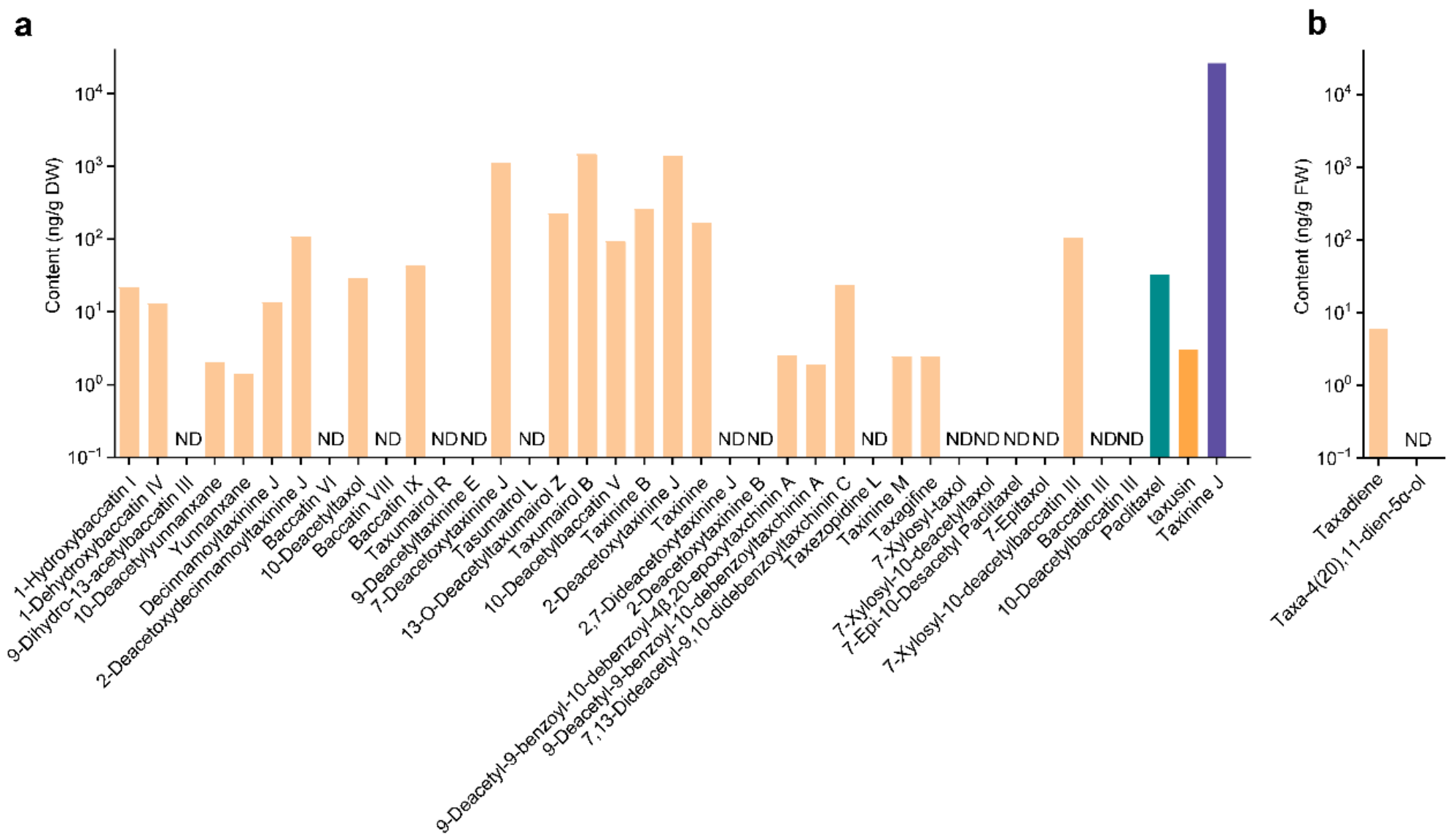

Supplementary Fig. 17 | Major taxoids in *Pseudotaxus chienii* leaf. a Content of major taxane expressed as ng/g dry weight (DW). **b** Contents of selected toxoids

determined on fresh weight basis (ng/g FW)**.** Metabolites that were undetected in the assay were indicated by "ND" (Not Detected).

R2Q8: The authors attribute reduced paclitaxel accumulation to deleterious sequence changes and functional divergence, but direct evidence for such mutations (e.g., active-site substitutions) is missing. Protein modeling and mutation mapping could substantiate the argument

Response: Thank you very much for this highly constructive suggestion. We completely agree that direct structural evidence would substantially strengthen the interpretation that functional attenuation contributes to the reduced paclitaxel accumulation observed in *P. chienii*. Therefore, we incorporated AI-assisted structural modeling, molecular docking, and molecular dynamics simulations to compare the *Taxus mairei* TOT (TmTOT) and *P. chienii* TOT (PcTOT) proteins in the revised manuscript.

Although no obvious catalytic-site truncation or large-scale structural disruption was observed (with an overall structural similarity RMSD of 1.67 Å), comparative structural analyses revealed notable conformational differences between the two proteins. Specifically:

First, molecular docking analyses suggested that PcTOT adopts a less favorable catalytic geometry compared with TmTOT, including altered substrate-binding distance and angle parameters (2.7 Å/149.2° for PcTOT vs. 2.4 Å/157.7° for TmTOT).

Second, molecular dynamics simulations and CAVER analyses indicated distinct, region-specific flexibility patterns. While PcTOT exhibited pronounced mobility in the R1 segment, it displayed distinctly repressed flexibility (reduced RMSF) within the R2 and R3 regions. Crucially, these differentially rigidified regions spatially map to the residues lining the substrate-access channels leading to the catalytic pocket.

These results suggest that the functional attenuation of PcTOT may not primarily arise from catastrophic active-site mutations, but rather from subtle conformational

changes that reduce catalytic accessibility and dynamic flexibility of the substrate-binding pocket. We have now incorporated these structural analyses and corresponding discussion into the Results (**lines 359-374**) and Methods (**line 658-674**) sections of the revised manuscript to provide additional mechanistic support for the observed enzymatic degeneration in *P. chienii*.

Page 9-10, lines 359-374, "…To preliminarily elucidate the reasons for the attenuated function of PcTOT compared to TmTOT, structural modeling and molecular docking analyses were first performed. The results revealed a slightly more relaxed catalytic conformation in PcTOT (2.7 Å/149.2°) compared to the highly optimized geometric parameters observed in TmTOT (2.4 Å/157.7°) (Supplementary Fig. 19a**)**, despite their overall structural similarity with a root-mean-square deviation (RMSD) of 1.67 Å across 2,766 backbone atoms (Supplementary Fig. 19b**)**. Intriguingly, molecular dynamics simulations revealed distinct, region-specific flexibility patterns across these structural regions. Specifically, PcTOT exhibited a pronounced conformational mobility within the R1 segment, yet displayed a distinctly repressed flexibility, evidenced by lower calculated residue-level root-mean-square fluctuation (RMSF) within R2 and R3 regions (Supplementary Fig. 19c**)**. Based on CAVER analysis, these differentially rigidified regions were spatially mapped onto the residues lining the substrate access channels leading to the buried catalytic pocket (Supplementary Fig. 19d**)**. Taken together, these complementary static and dynamic analyses suggest that the altered flexibility and subsequent channel gating dynamics likely contribute to the divergent catalytic behaviors of the two enzymes."

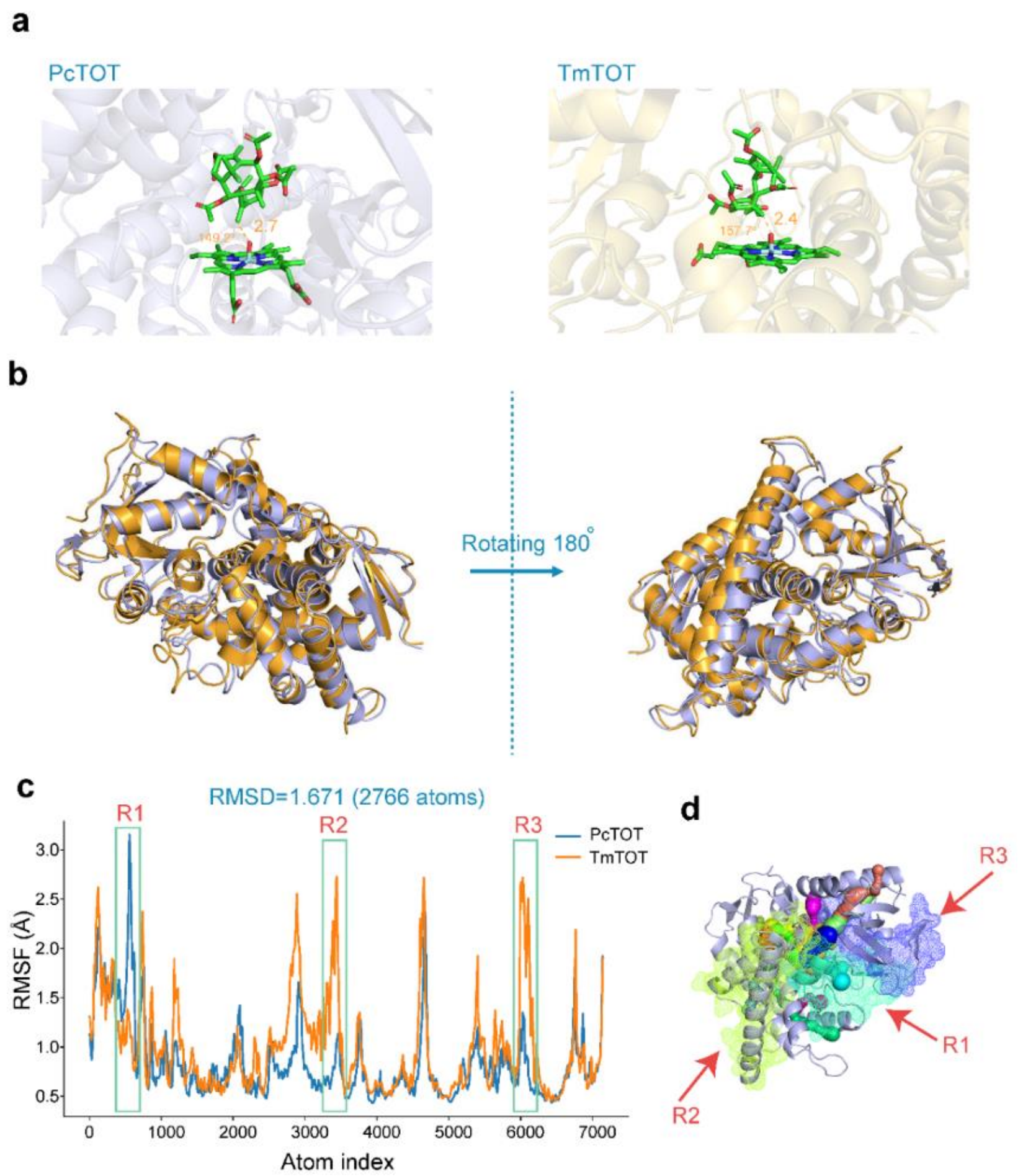

Supplementary Fig. 19 | Structural comparison and molecular dynamics simulation of PcTOT and TmTOT. a Active site alignment of PcTOT (left) and TmTOT (right). The taxane substrate (upper sticks) is positioned directly above the catalytic heme co-factor (lower sticks). Orange dashed lines indicate the distance (Å) and reaction angle (degrees) between the heme-activated oxygen and the target atom of the substrate. **b** Structural superposition of the overall models of PcTOT (light blus) and TmTOT (orange) shown from two perspectives rotated by 180° (root-mean-square deviation (RMSD) = 1.671 Å across 2766 atoms). **c** Root-mean-square fluctuation (RMSF) profiles calculated from 200-ns molecular dynamics simulations. The blue and orange lines represent the atomic fluctuations of PcTOT and TmTOT, respectively. The green boxes highlight three specific structural flexibility regions, designated R1, R2, and R3. **d** Three-dimensional spatial mapping of the R1, R2, and R3 regions (indicated by red arrows and meshes) on the 3D enzyme structure, illustrating their physical locations relative to the substrate-binding pocket and access channel.

R2Q9: The manuscript convincingly demonstrates low paclitaxel but high taxinine J accumulation in *P. chienii* and suggests that prioritizing taxinine J is an evolved defense strategy. Is there any ecological or bioactivity evidence suggesting that taxinine J is more effective than paclitaxel against the specific pathogens or herbivores found in *P. chienii*'s habitat? Without this context, the "adaptive" argument remains speculative.

Response: Thank you for this insightful comment. We completely agree that claiming an "adaptive" advantage for taxinine J is speculative without direct ecological evidence. To address this, we have removed the statements describing taxinine J as a "primary chemical defense" from the revised Discussion. Instead of making ecological assumptions, we now focus solely on the biochemical mechanism. As revised in the Discussion (**lines 496-502**), we explain the high taxinine J accumulation simply as a consequence of the downstream functional bottleneck. We clarify that the functional degeneration at the later catalytic steps restricts the paclitaxel pathway, which naturally favors the accumulation of alternative branch products like taxinine J. This revision ensures that our conclusions do not overreach and are fully grounded in the genomic, targeted metabolite, and enzymatic data presented in our study.

Page 13, lines 496-502, "…Instead, metabolic flux appears to be preferentially channeled toward alternative branches, particularly taxinine J. This metabolic rewiring is evidenced by the marked accumulation of taxinine J, along with discernible levels of taxusin in specific spatiotemporal samples, and the consistent absence of downstream intermediates like baccatin Ill across sampled wild populations. Far from indicating a complete evolutionary loss, this specific taxane accumulation profile provides a biochemical basis for the restriction of complete paclitaxel biosynthesis to an ultra-rare, conditionally dependent trace event."

R2Q10: The methods section states that experiments were performed on leaves 6, 7, and 8 of the same N. benthamiana plant as three biological replicates, each consisting

of fourleaf discs. However, the Results section lacks data representing these replicates (e.g., error bars, SD/SEM, or individual data points). Please provide the underlying data for these replicates or incorporate statistical error bars into the figures to show experimental consistency.

Response: We thank you for this helpful comment. The presentation of biological replicates and experimental variability is important for evaluating the robustness and reproducibility of the transient expression assays. We revised Figure 3b to explicitly include individual biological replicate data points together with statistical error bars (mean ± SD) for the corresponding quantitative analyses. These revisions now more clearly illustrate the consistency and reproducibility of the experiments performed using leaves 6, 7, and 8 from the same *Nicotiana benthamiana* plant, with each biological replicate consisting of four leaves discs as described in the Methods section.

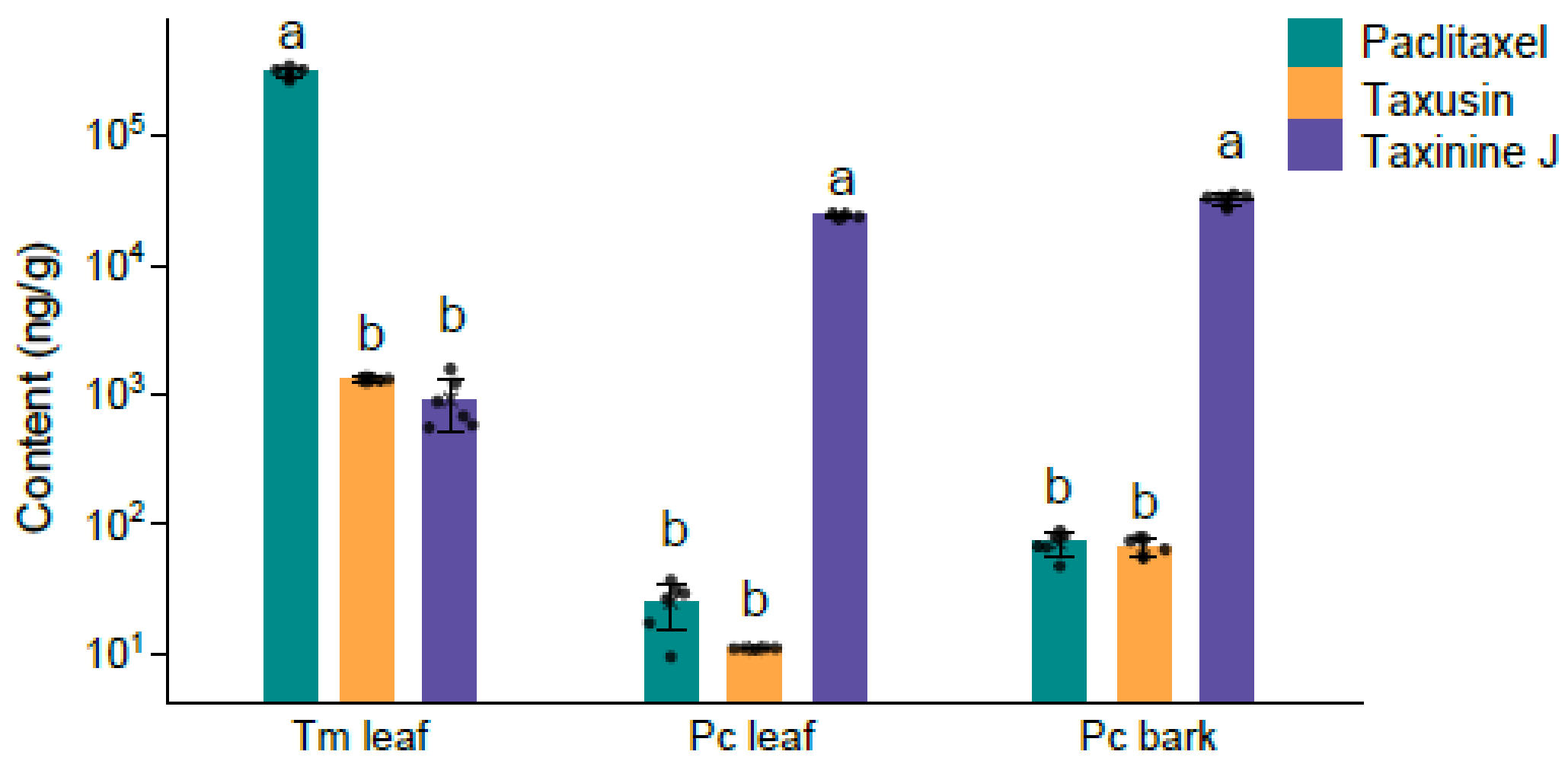

R2Q11: The conclusion regarding the low paclitaxel accumulation relies heavily on the reduced catalytic efficiency of *P. chienii* TOT. Quantitative kinetic parameters (Km, Kcat, Vmax, or relative rate ratios) would strengthen the functional argument beyond qualitative rate comparisons. Furthermore, only five downstream genes were tested. Is PcTOT the only rate-limiting enzyme identified? A more comprehensive analysis of the downstream pathway enzymes (e.g., T1βH, BAPT, T3'NBT, DBTNBT) would

strengthen the claim that metabolic flux is shifted specifically towards taxinine J due to this specific bottleneck.

Response: We thank you for this important suggestion. The broader functional characterization of downstream paclitaxel biosynthetic enzymes is essential for evaluating whether the observed metabolic phenotype in *P. chienii* reflects a localized enzymatic constraint or a more extensive pathway-level attenuation. Consequently, we substantially expanded the Results section "Functional characterization identifies a severe downstream bottleneck in paclitaxel biosynthesis" by incorporating additional heterologous functional analyses of multiple late-stage enzymes. Specifically, as detailed in the revised manuscript (**lines 374-377, lines 474-481**), we tested six core later candidates across two functional modules: oxygenases (T9ox, T9H-750C, and T1βH) and terminal acyltransferases (BAPT, T3'NBT. and DBTNBT). These results revealed that, in contrast to their characterized *Taxus* homologs, the corresponding *P. chienii* enzymes exhibited no detectable catalytic activity under our experimental conditions. Importantly, this pattern was consistently observed across several independent downstream modules rather than being restricted to PcTOT alone. Together with the severely reduced activity observed for PcTOT, these results indicate that the terminal paclitaxel biosynthetic network in *P. chienii* has undergone broad functional attenuation.

Accordingly, we revised the manuscript to avoid overemphasizing PcTOT as the sole rate-limiting step. Instead, we now describe the downstream pathway as exhibiting "profound functional attenuation of the terminal paclitaxel biosynthetic pathway" (**line 488**) and "pronounced functional degeneration at the later catalytic steps" (**line 516**), supported by the collective loss or reduction of activity across several terminal biosynthetic enzymes.

Regarding quantitative kinetic characterization of PcTOT, we fully acknowledge that parameters such as Km, Kcat, and Vmax would provide valuable mechanistic detail. However, rigorous kinetic measurements were technically difficult because PcTOT displayed extremely weak and unstable catalytic behavior in heterologous systems,

with product formation frequently near the analytical detection threshold. In addition, the authentic taxane substrates required for precise steady-state kinetic analyses are difficult to obtain in sufficient purity and quantity. Under these conditions, we considered qualitative activity comparisons to be more reliable and scientifically conservative than presenting low-confidence kinetic estimates.

Taken together, these convergent data support a model in which metabolic flux toward canonical paclitaxel biosynthesis is severely constrained by widespread downstream pathway degeneration, thereby favoring accumulation of alternative taxane branches such as taxinine J.

Page 10, lines 374-377, "Moreover, despite genomic retention and high sequence conservation with Taxus orthologs, heterologous expression of six core downstream candidates, including T9ox and BAPT, in *Nicotiana benthamiana* failed to yield detectable pathway products, in sharp contrast to active T. mairei counterparts (Supplementary Fig. 20)."

Page 12, lines 474-485, "To systematically investigate whether this functional deficiency extends across the downstream pathway, we characterized two core late-stage functional modules. The first module contains key oxygenases involved in skeleton modification, including T9ox (65.3% sequence identity to the *Taxus* ortholog), T9αH-750C (68.7% identity), and taxane 1β-hydroxylase (T1βH-184) (68.2% identity). The second module includes terminal acyltransferases responsible for side-chain formation and attachment, namely baccatin III:3-amino-3-phenylpropanoyl transferase (BAPT) (67.8% identity), taxoid-3′-N-benzoyltransferase (T3'NBT) (73.0% identity), and 3'-N-debenzoyl-2'-deoxypaclitaxel-N-benzoyl transferase (DBTNBT) (71.9% identity). Despite genomic retention and considerable sequence conservation, none of the genes from either downstream module produced detectable pathway products in *Nicotiana benthamiana*, whereas the functional competence of the corresponding *T. mairei* orthologs remained intact (Supplementary Fig. 20)."

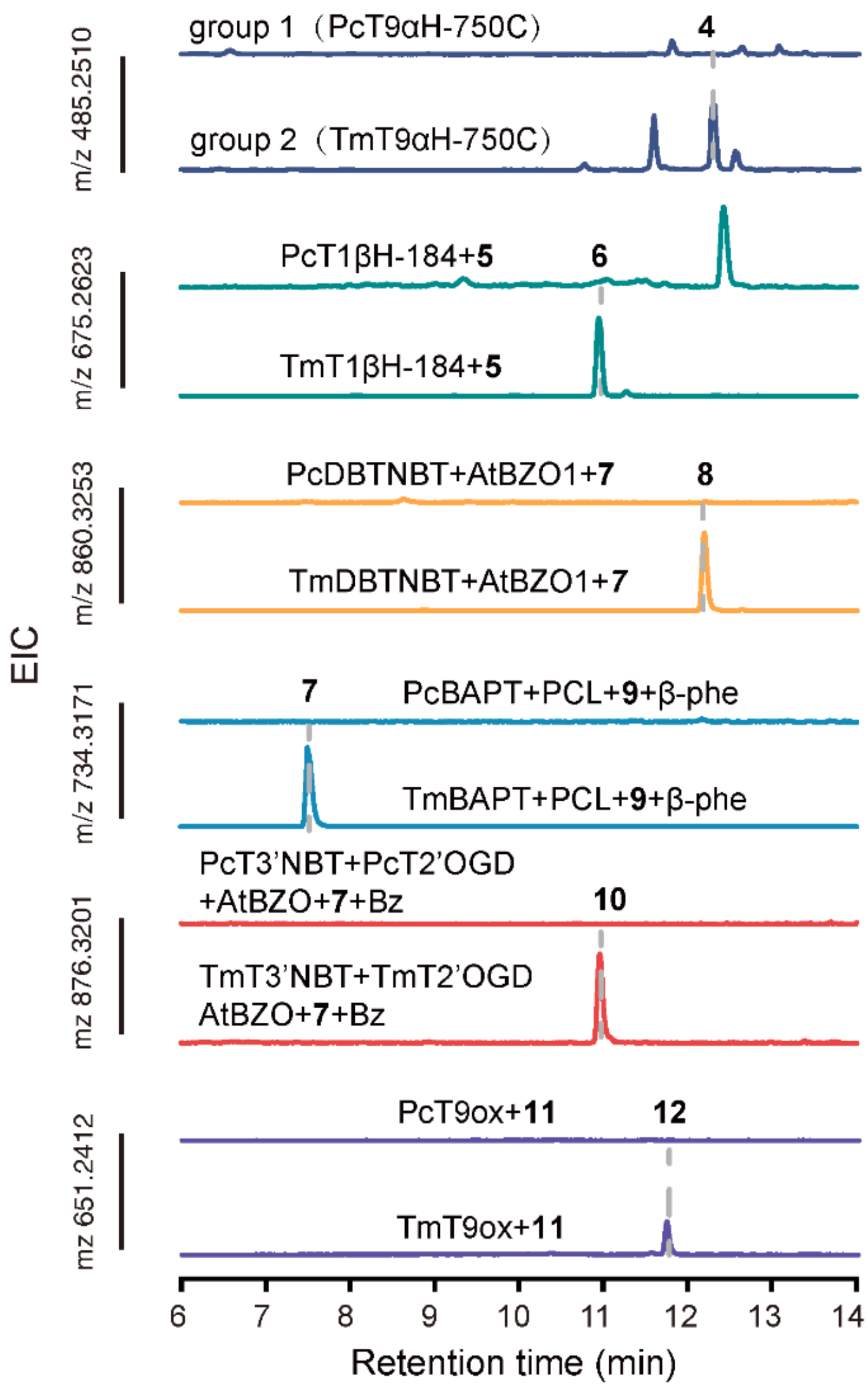

Supplementary Fig. 20 | Functional characterization of candidate paclitaxel biosynthesis genes from *Pseudotaxus chienii*. In vivo enzymatic activities of candidate genes from *P. chienii* (group 1) were assayed using a transient expression system in tobacco leaves. The corresponding orthologous genes from *T. mairei* (group 2) were used as positive controls in all assays. Extracted ion chromatograms (EICs) illustrate the production of target metabolites across different functional validation assays: De novo synthesis of 13a-deacetyltaxusin (**4**) by co-expressing *PcT9aH-750C* with upstream biosynthesis genes. Production of baccatin IV (**6**) in leaves expressing *PcT1βH-184* following the infiltration of 1-dehydroxybaccatin IV (**5**). Production of 2' -deoxypaclitaxel (**8**) in leaves co-expressing *PcDBTNBT* and *AtBZ01* with the infiltration of 3'-debenzoyl-2 -deoxypaclitaxel (**7**). Synthesis of **7** in leaves co-

expressing *PcBAPT* and *PCL* (a phenylalanine-CoA ligase with an A312G mutation from *Penicillium chrysogenum*) supplemented with baccatin I (**9**) and Β-phenylalanine (β-phe). Production of paclitaxel (**10**) in leaves expressing *PcT3'NBT*, *PcT2'OGD*, and *AtBZO1* with the infiltration of **7** and benzoic acid (Bz). Production of 13-acetylbaccatin IIl (**12**) in leaves expressing *PcT9ox* following the infiltration of 9-dihydro-13-acetylbaccatin IIl (**11**).

R2Q12: The authors propose that the coordinated function of the dispersed TBA depends on "higher-order spatial chromatin organization." While this is a logical hypothesis, the Discussion does not explicitly state if Hi-C interaction data supports physical interactions between these distant loci (~80.7 Mb apart). If such data is available, it should be highlighted to substantiate the claim that regulatory coherence transcends physical continuity. If not, the language should be tempered to reflect that this is a hypothesis rather than a confirmed mechanism.

Response: We appreciate this suggestion. We substantially revised the relevant Results (**lines 223-237**) and Discussion (**lines 408-418**) sections and incorporated additional Hi-C-based chromatin architecture analyses to better support the proposed TBA model. Specifically, our revised analyses show that the dispersed taxane biosynthetic regions are consistently localized within transcriptionally active A compartments and organized into coordinated TAD/sub-TAD chromatin environments. In addition, these dispersed loci exhibit highly coordinated transcriptional behavior across the integrated transcriptomic datasets. Together, these observations support the conclusion that the TBA is not merely a collection of physically separated gene clusters, but rather represents a functionally coordinated chromatin-regulatory unit despite large genomic distances (~80.7 Mb). Importantly, we agree with the reviewer that the current Hi-C analyses do not directly resolve stable long-range chromatin looping interactions among all dispersed TBA loci. Therefore, we revised the manuscript to avoid overstating this point. Instead of claiming experimentally confirmed physical interactions, we now describe higher-order chromatin organization as a plausible

regulatory framework that may facilitate coordinated expression and functional integration of dispersed pathway components.

Page 6, line 223-237, "To quantitatively validate the functional integration of this dispersed genomic archipelago, we combined chromatin architecture profiles derived from Hi-C analysis (using the Pca haplotype as a representative model) with Pearson correlation analyses of a comprehensive 128-sample transcriptomic dataset. Hi-C profiles revealed that the entire TBA region is embedded within the transcriptionally active A compartment[27,28], providing a globally favorable environment for gene expression. Notably, the three gene clusters are spatially segregated into distinct Topologically Associating Domains (TADs)[29,30], which further subdivide into localized sub-TADs (Supplementary Fig. 12a). This hierarchical division closely aligns with functional boundaries; for example, the TAD encompassing TS1C exclusively harbors core metabolic genes, effectively demarcating the functional domain by excluding a non-metabolic gene at its left border. Although separated into independent TADs, metabolic genes from the three clusters and these dispersed core enzymes form a highly coordinated co-expression network, with the majority of exhibiting Pearson correlation coefficients (r) ≥ 0.9 (Supplementary Fig. 12b). Collectively, these findings indicate that the functional integrity of the TBA is determined by regulatory coordination and active chromatin occupancy, rather than strictly by linear physical proximity."

Page 10-11, line 408-418, "…Traditional models generally invoke direct chromatin looping to account for long-range co-expression, whereas our findings reveal a distinct spatial regulatory paradigm in expanded gymnosperm genomes. Although spatially segregated into distinct TADs, the three TBA clusters maintain robust regulatory synchrony without relying on direct physical contact. Instead, this coordinated expression is driven by spatial co-localization within the transcriptionally active A compartment, coupled with unified regulation by trans-regulatory networks. This insight refines the evolutionary understanding of specialized metabolism. In large expanded genomes, the integrity of biosynthetic pathways does not require linear

contiguity or complex 3D chromatin folding. Rather, functional modules can be isolated via precise topological insulation and synchronously modulated by common global regulatory signals[48]".

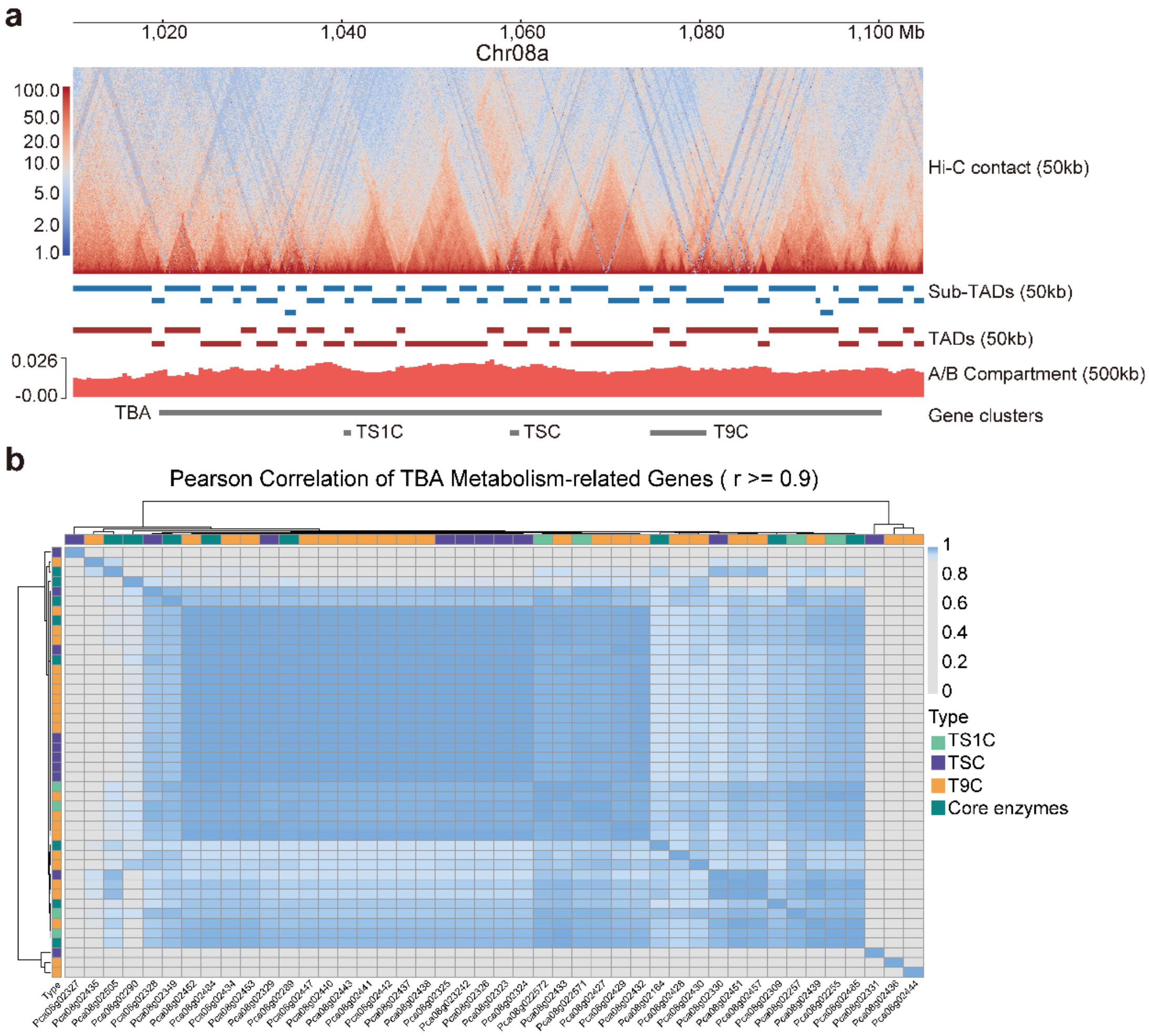

Supplementary Figure 12 | Chromatin structure and co-expression network of metabolism-related genes in the TBA region of Pca. a Hi-C interaction map and chromatin features in the TBA region. Each track (from top to bottom) shows the Hi-C contact frequency, sub-TADs, TADs, A/B compartments, TBA, and the genomic location of the three gene clusters (TS1 Cluster (TS1C), Taxadiene Synthase Cluster (TSC), and T9 Cluster (T9C)). **b** Pearson correlation heatmap of metabolism-related genes in TBA. Based on a transcriptome dataset containing 128 samples, these genes collectively form a highly coordinated co-expression network, with the vast majority of gene pairs having a Pearson correlation coefficient (r $\geqslant$ 0.9).

R2Q13: Line 83, 101, 103, 106, 127, 130： Supplementary Tables 2, 3, 4, and 5 were not found in the supplemental files.

Response: We thank you for carefully checking the supplementary materials and apologize for the confusion. The missing Supplementary Tables 2, 3, 4, and 5 (now Supplementary Tab. 1, 2, 4, 5, 6) have now been added to the revised supplemental files. Corresponding references in the main text have also been checked and updated to ensure consistency and accessibility.

R2Q14: Line 85： Please specify "contig N50" instead of just "N50" to avoid ambiguity and ensure clarity regarding the genome assembly quality metrics.

Response: We sincerely thank you for your valuable reminder. We have replaced all mentions of "N50" with contig N50 to clarify the genome assembly metric and avoid ambiguity.

Page 3, lines 116-117, "…Hap1 spans 15.434 Gb (725 contigs; contig N50 60.2 Mb) and Hap2 spans 15.430 Gb (817 contigs; contig N50 55.9 Mb)."

R2Q15: Lines 134-137: Indicate how divergence times were estimated (e.g., molecular clock model, calibration fossils).

Response: Thank you for your comment. We would like to clarify that these details have been included in our methodology. In the original manuscript, we specified that the divergence times were estimated using a relaxed molecular clock model, with fossil calibration points from the TimeTree database. To make this information stand out more clearly, we have revised the description in the Methods section to read:

Page 15, lines 602-605, "Specifically, divergence times were estimated using MCMCTree (PAML v4.9j)[80] under a relaxed molecular clock model. To calibrate the

absolute evolutionary timeline, fossil-calibration points obtained from the TimeTree database[81] were applied to specific nodes."

R2Q16: Lines 170-172: There is a discrepancy in the number of candidate biosynthetic gene clusters (BGCs) reported between the text, figures, and tables. Please cross-check these numbers and ensure consistent labeling and reporting throughout the manuscript. Clarify whether identical BGC identification criteria (plantiSMASH parameters) were used for both haplotypes.

Response: We thank the reviewer for carefully checking the consistency of the BGC annotations and reporting. We systematically re-examined the manuscript, figures, tables, and supplementary materials to ensure that the reported numbers of candidate biosynthetic gene clusters (BGCs) are fully consistent throughout the revised version.

In addition, the statement in the Results section indicating "21 candidate biosynthetic gene clusters (BGCs) in Pca and 25 in Pcb" specifically refers to the candidate BGCs identified within the 80.7-Mb taxane-associated region on chromosome 8. In contrast, the numbers reported in the supplementary datasets correspond to the total number of BGCs identified genome-wide across the entire haplotype assemblies.

Futhermore, we confirm that identical plantiSMASH identification parameters and filtering criteria were applied to both haplotypes throughout all analyses. This information has now been added to the Methods section for clarity and reproducibility. We appreciate the reviewer's attention to this issue, which helped improve the precision and consistency of the manuscript.

Page 16, line 615-617, "The BGC were initially identified using plantiSMASH v2.0.0[87] with default parameters to detect candidate clusters based on gene proximity and conserved domain signatures across both haplotypes (with same parameters) of the *P.chienii* genome."

R2Q17: Line 171: Supplementary Figure 10 lacks panel labels (e.g., a, b). Please add these labels to the figure and ensure they correspond with the descriptions in the figure legend.

Response: Thank you for your careful examination. We apologize for any confusion. We would like to clarify that Supplementary Fig. 10 is a single, comprehensive image rather than a multi-panel figure.It displays the overall chromosomal distribution of secondary metabolism-related gene clusters across the genomes. Therefore, sub-panel labels (e.g., a, b) are not applicable to this specific figure.

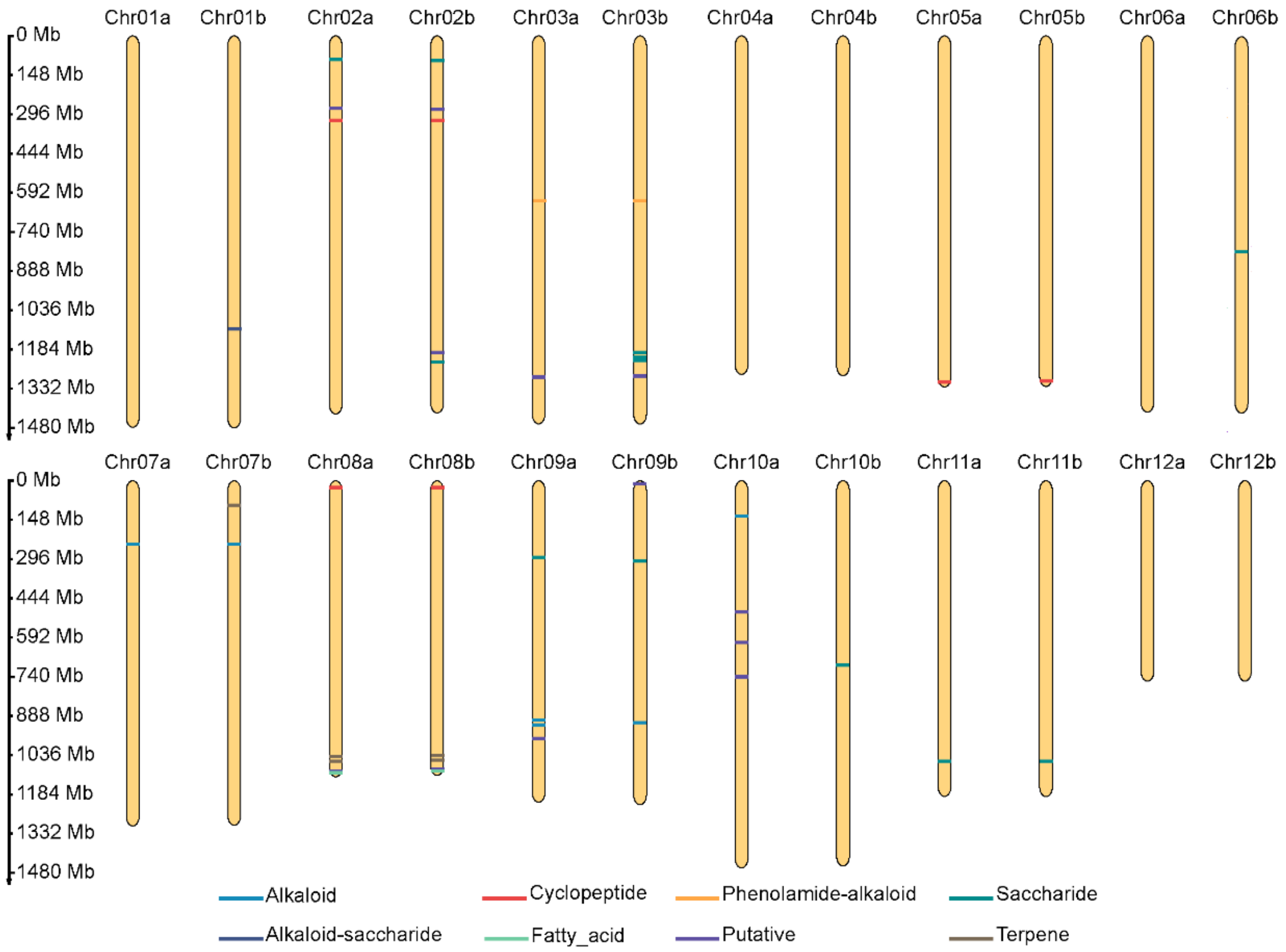

Supplementary Fig. 10 | The chromosomal distribution of secondary metabolism-related gene clusters in *Pseudotaxus chienii*. Color bars and labels indicate key enzyme-coding genes involved in pathways such as esters (putative), lignans, alkaloids, terpenes, and polyketides.

R2Q18: Lines 188-190: Please include a schematic diagram comparing the "archipelago" organization in *P. chienii* vs. compact BGCs in angiosperms for intuitive interpretation.

Response: We thank you for this valuable suggestion. We have added a new schematic diagram comparing the Taxane Biosynthetic Archipelago (TBA) organization in *P. chienii* with the canonical compact biosynthetic gene cluster (BGC) organization commonly observed in angiosperms (**line 240**).

The newly added schematic illustrates the major conceptual differences between these two genomic architectures. Specifically, the figure highlights that taxane biosynthetic genes in *P. chienii* are distributed across multiple physically separated but syntenically conserved genomic islands spanning an ~80.7 Mb region, whereas angiosperm BGCs are typically organized as tightly co-localized gene clusters within a compact chromosomal locus.

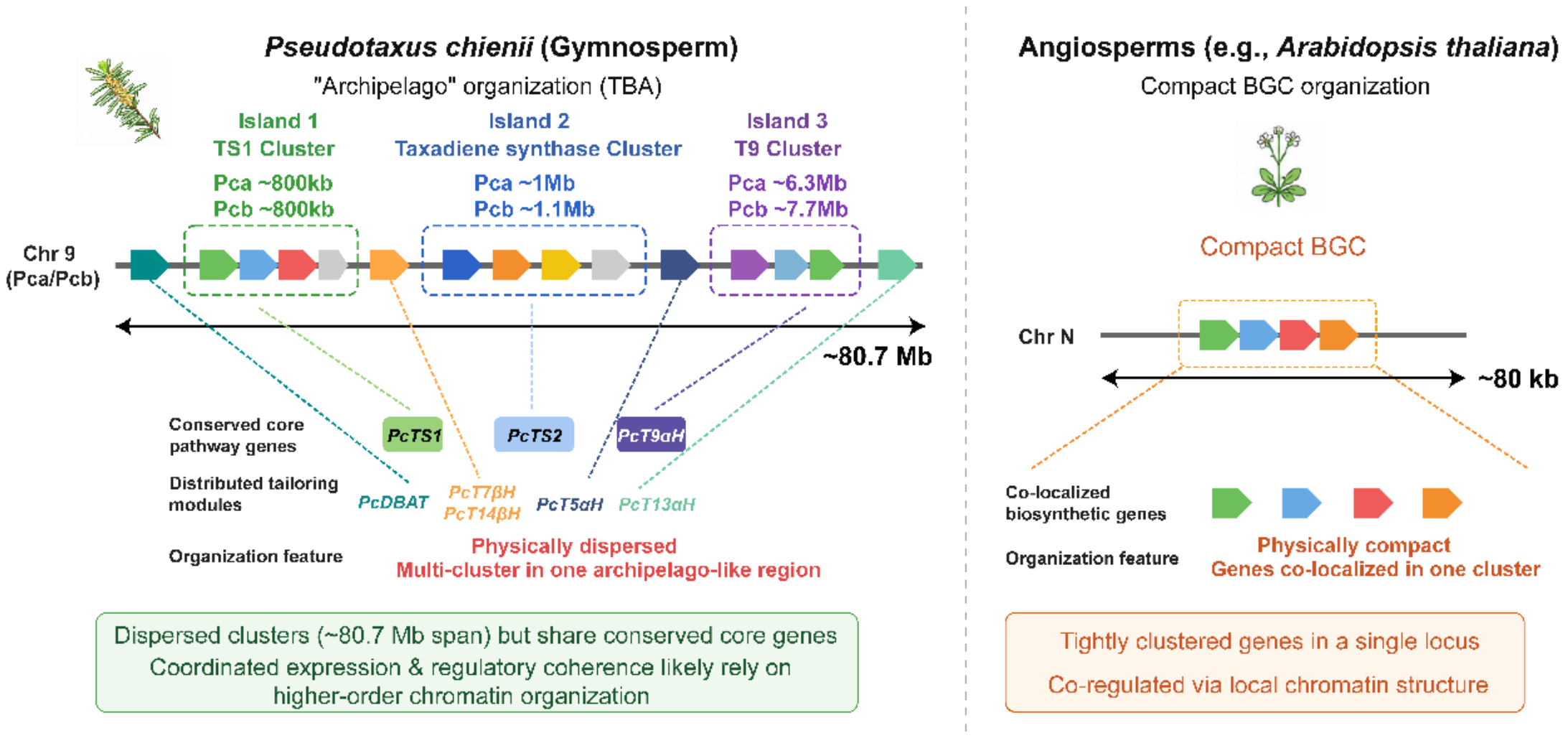

Supplementary Figure 13 | Schematic comparison of the "archipelago" organization in *Pseudotaxus chienii* versus compact biosynthetic gene clusters (BGCs) in angiosperms. In *P. chienii* (left), the Taxane Biosynthetic Archipelago (TBA) spans a massive genomic region (~80.7 Mb), featuring physically dispersed gene cluster (TS1 Cluster (TS1C), Taxadiene Synthase Cluster (TSC), and T9 Cluster (T9C)) and scattered core pathway genes (e.g., *PcDBAT*, *PcT5αH*, *PcT13αH*) that likely rely on higher-order chromatin organization for coordinated expression. In contrast, typical angiosperm BGCs (right) are physically compact (e.g., ~80 kb), with co-localized

biosynthetic genes tightly clustered in a single locus and co-regulated via their local chromatin structure.

R2Q19: Lines 197-198：The authors mention a translocation event but do not refer to a specific figure. Please cite the relevant figure(s) that provide visual or analytical evidence for this genomic structural variation.

Response: We thank you for pointing out this omission. In the revised manuscript, we have added the appropriate figure citations in the relevant section describing the translocation event.

Page 7, line 246-249, "For instance, the T9C (collinear with the previously defined Group 9.1 in *T. mairei*[7]) highlights this plasticity (Fig. 2b), with the TOT genes varying from three copies in Pca to a single copy in Pcb, and appearing translocated near the TS1C in the *T. mairei* (Fig. 2b, c)."

R2Q20: Lines 233-234: Clarify whether taxinine J detection relied on internal reference standards or MS fragmentation matching only.

Response: Thank you for your question. In the revised manuscript, we clarified that taxinine J (CAS: 18457-46-0; BBP04119, BioBioPha, China) identification was performed using authentic reference standards in combination with LC-MS/MS fragmentation patterns and retention time matching. This information has now been explicitly added to the Methods section to improve transparency and reproducibility. We have added detailed information in the manuscript.

Page 19, lines 751-754, "Calibration curves (0.5 – 1000 ng mL^{-1}) of taxinine J (CAS: 18457-46-0), taxusin (CAS: 19605-80-2), Baccatin III (CAS: 27548-93-2) and paclitaxel (CAS: 33069-62-4) standards, as well as other target metabolites analyzed in

Nicotiana benthamiana leaves, were prepared by serial dilution of authentic standards in 80% methanol to match the sample solvent matrix."

R2Q21: Line 240: Replace "trace leaves" with "trace amount in leaves."

Response: Thank you for your suggestion. We have corrected the phrase to "trace amount in leaves" to improve the accuracy of our expression.

Page 8, lines 320-321, "Within this initially sampled *P. chienii* individual, both paclitaxel and taxusin remained at trace amount in leaves, whereas taxinine J accumulated to substantially higher levels (Fig. 3b)."

R2Q22: Lines 250-252, 436-437: To ensure experimental reproducibility, please provide a comprehensive list of all primer sequences used for gene cloning. It is recommended to include this information in supplementary file.

Response: We thank you for this helpful suggestion. We have compiled and added a comprehensive list of all primer sequences used for gene cloning and related experiments in the revised supplementary files (**Supplementary Data 6**). Corresponding references have also been added in the Methods section to facilitate experimental reproducibility.

R2Q23: Line 264: The phrase "inefficient removal of ancient repetitive elements" is ambiguous. Briefly mentioning the mechanism (e.g., lack of unequal recombination) in the context of *P. chienii* specifically would make the argument clearer for non-genomics specialists.

Response: We sincerely thank you for this insightful comment. Following your suggestion, we have reframed the description to directly incorporate the specific

mechanism ("lack of unequal recombination") while also improving the overall phrasing to make the statement more direct.

Page 10, line 395-397, "The larger genome of *P. chienii* was primarily attributed to the inefficient removal of ancient repetitive elements resulting from a lack of unequal recombination, rather than more frequent transposition[46,47]."

R2Q24: Line 352: Please include the formal citation for the GCEv1.0.0.

Response: Thank you for your question. We have revised the software version to GCE v1.0.2 at line 477 and added its formal citation as required.

Page 14, lines 557-559, "Additionally, genome size, heterozygosity, and repetitive element content were determined using K-mer (21-mer) analysis performed with GCE v1.0.2[53] on all clean MGI short-read data, evaluating K-mer depth distributions and peak features."

R2Q25: Line 440: Please provide appropriate citations for the Agrobacterium-mediated transformation protocol to support the methodology described in this section.

Response: We appreciate your suggestion. We have added a classic formal citation for the freeze-thaw transformation protocol of *Agrobacterium tumefaciens* GV3101 in the revised manuscript.

Page 15, line 574-575, "The plasmids containing the genes were transformed into *Agrobacterium tumefaciens* (strain GV3101) cells using the freeze–thaw method[107]."

R2Q26: Improve figure labeling for readability (e.g., Fig. 2b/c).

Response: We thank you for this suggestion. We have revised the labeling, font size, and overall layout of the affected figures, including Fig. 2b/c, to improve readability and visual clarity throughout the manuscript.

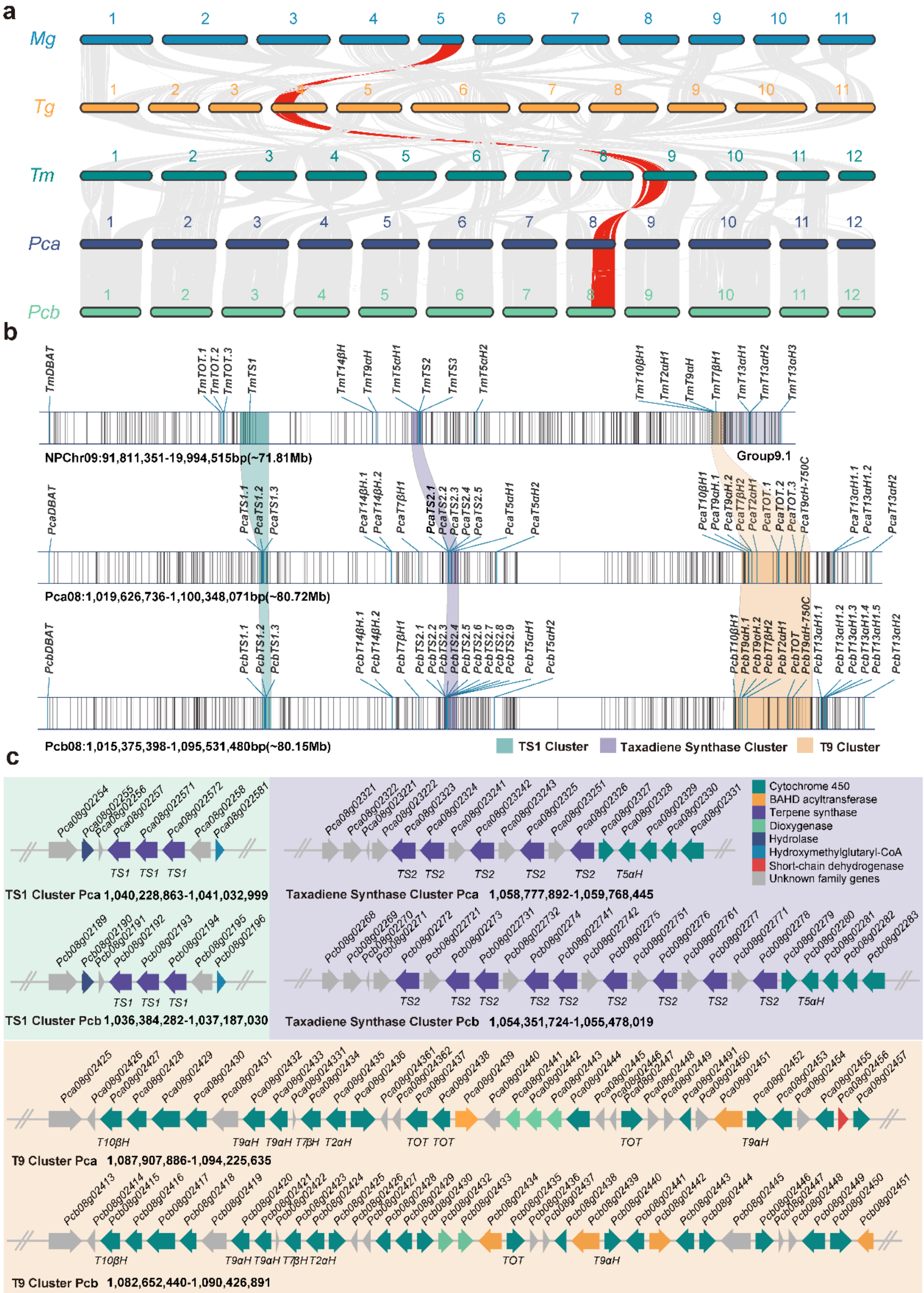

Fig. 2 | Genomic organization and structural features of Taxane Biosynthetic Archipelago (TBA) in *Pseudotaxus chienii*. a Macrosynteny among three *Taxaceae* species and the *Cupressaceae* outgroup *M. glyptostroboides* (Mg) highlights a conserved region containing three biosynthetic gene clusters (BGCs) and colinear upstream genes, including *TS*, *T5αH*, *T10αH* and *T13αH*. Tg, *T.grandis*; Tm, *T.mairei*; Pca/b, *P. chienii* hap1/hap2. **b** Colinear arrangement of paclitaxel biosynthetic genes within the TBA region. Genes involved in the paclitaxel biosynthetic pathway are shown in red. **c** Organization of three paclitaxel-related BGCs on *P. chienii* chromosome Pca08. Genes are color-coded by enzymatic function, including terpene synthases, cytochrome P450s and downstream tailoring enzymes involved in taxane modification.

R2Q27: In Figure 3A, the meaning of the different colored blocks following each gene is unclear.

Response: We thank you for noting this ambiguity. In the revised figure legend for Figure 3A, we now explicitly clarify that the different colored blocks represent distinct gene copies in *P. chienii* hap1, which were color-coded to facilitate visualization and differentiation among duplicated gene copies.

Page 31, line 1107, "Different colors represent different gene copies in *P. chienii* hap1."

R2Q28: Define abbreviations (e.g., T5αH, TS, T13αH) upon first mention in the text.

Response: We have carefully checked all abbreviations throughout the manuscript and clearly defined them at their first occurrence.

Reviewer #3:

Summary

Reviewer Comments on NCOMMS-25-103630 Archipelago architecture and evolution of paclitaxel

l biosynthesis revealed by the *Pseudotaxus chienii* haplotype-resolved genome.

The study presents a high-quality haplotype-resolved genome assembly of *Pseudotaxus chienii*, representing a substantial technical and conceptual advance for investigating the evolution of the taxanes biosynthetic pathway. The haplotype-level resolution is particularly valuable for interrogating genome organization, structural variation, and metabolic gene clustering, especially given the high degree of heterozygosity observed in *P. chienii*.

In this context, the conclusions of the present study contrast with those of the recent work by Wang M. et al. (2025) (https://www.nature.com/articles/s41467-025-67849-4), which reported that *P. chienii* harbors only a partial taxane biosynthetic pathway, terminating prior to the biosynthesis of 10-deacetylbaccatin III, based on a chromosome-scale genome assembly. By leveraging a haplotype-resolved genome assembly, the authors of the present study were able to identify and experimentally validate some orthologs of the paclitaxel biosynthetic pathway.

Notably, the high level of haplotypic divergence implies substantial intraspecific genetic variability, which, in interaction with environmental factors, may contribute to the existence of distinct chemotypes within *P. chienii*. This highlights the importance of considering genotype × environment interactions when interpreting metabolic diversity and pathway completeness in highly heterozygous species.

These results underscore the critical importance of high-resolution, haplotype-aware genome assemblies when studying complex metabolic pathways in species with high

heterozygosity, where collapsed or consensus assemblies may obscure essential genes or entire pathway modules.

However, several points require clarification or improvement to strengthen the conclusions and enhance the clarity of the manuscript.

Response: We are very grateful for the comments and suggestions you have raised.

R3Q1: Genome assembly and karyotype inference

The assembly reports 12 pseudochromosomes. It is unclear whether this number reflects the expected karyotype of the species or results from assembly or scaffolding choices. Please clarify the cytogenetic basis (if known) and explicitly state whether the pseudochromosome number corresponds to the actual chromosome number.

Response: We thank you for raising this point. While direct cytogenetic data like chromosome counting is currently unavailable for *Pseudotaxus chieni*, we are confident that the 12 assembled pseudochromosomes represent the actual biological chromosome number rather than an assembly artifact. This number is consistent with a recently published independent assembly of *P. chienii* (Wang et al., 2025) and matches the conserved haploid chromosom enumber (n = 12) of related species. Additionally, our Hi-C interaction maps show 12 distinct chromosome territories with no ambiguous links, and the total length of the assembled pseudochromosomes precisely matches our genome size estimations from flow cytometry and K-mer analysis. We have updated the Results section to clarify that the pseudochromosome number corresponds to the actual chromosome number and aligns with previous size estimates (**lines 113-116**).

Page 3, lines 113-116, "This yielded two haplotype-phased genomes (Hap1 and Hap2), both anchored to 12 pseudochromosomes, corresponding to the highly conserved haploid chromosome number (n = 12) of the *Taxus* species[21,22]. The total length of the assembled pseudochromosomes is consistent with our genome size estimations, the previously published genome of *P. chienii*[16]."

R3Q2: Genome size estimation by flow cytometry

Supplementary Figure 2 (a, b, c) is not sufficiently explicit to support the conclusions regarding genome size estimation.

• First, the peaks corresponding to T. marei and *P. chienii* should be clearly identified and labeled in panels 2a, 2b, and 2c.

• Second, I strongly suggest summarizing the flow cytometry data in a table, specifying for each species:

• species name

• C-value (1C genome size)

• PI-A peak position

• estimated nuclear DNA mass

Such a table would clearly support your results.

Response: We thank you for these constructive suggestions and apologize for our oversight in the previous submission. During revision, we discovered that an incorrect flow cytometry image had inadvertently been included in the original Supplementary Fig. 2, and the figure did not correspond to the *P. chienii* measurements described in the manuscript. In the revised version, we have replaced Supplementary Fig. 2 with the correct flow cytometry results. The updated panels now clearly display and label the peaks corresponding to *T. mairei* and *P. chienii* in Fig. S2 a–c. The corrected data clearly show that the nuclear fluorescence signal (and corresponding genome size) of *P. chienii* is approximately 1.5-fold greater than that of *T. mairei*, consistent with our genome assembly and size estimation results. In addition, following your recommendation, we have now added the previously missing flow cytometry summary table as Supplementary Tab. 1.

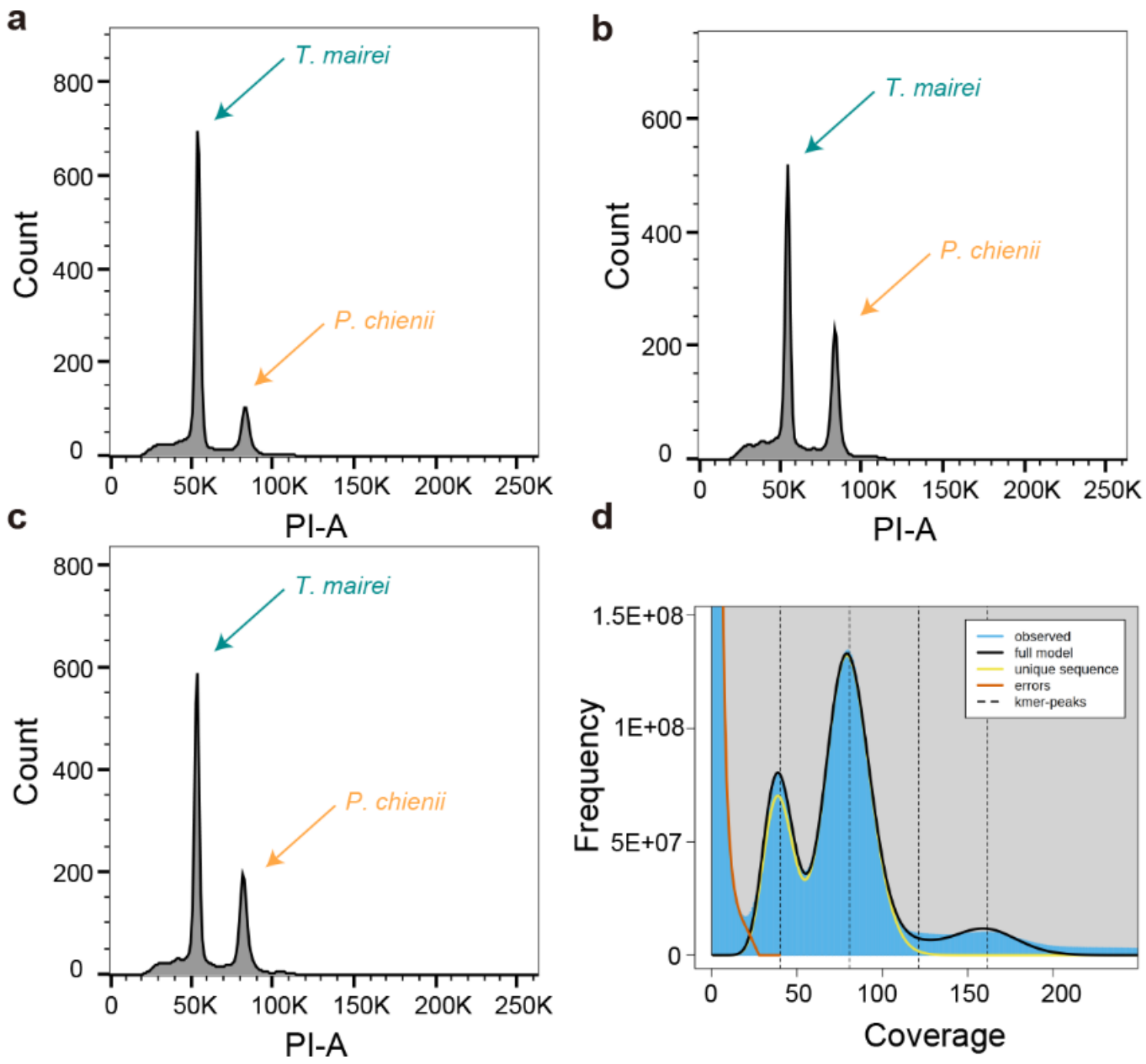

Supplementary Fig. 2 | Genome size assessment. a-c Flow cytometry results of genome size estimation. a, b, c are three experimental replicates, peak 2 represents the internal reference of *T. mairei* (10.23Gb), and peak represents *P. chienii*. PI-A, propidium iodide area. **d** *K-mer*-based genome size prediction. The first peak presents heterozygous *K-mers* and the second peak presents homozygous *K-mers*. The estimated monoploid genome sizes and heterozygosities are shown. The first peaks of both results are located at 40 ×, corresponding to second peaks at 80 ×.

Supplementary Tab. 1 | Statistics of flow cytometry estimation of genome sizes

Type	R1		R2		R3	
	PI-A Mean	PI-A %CV	PI-A Mean	PI-A %CV	PI-A Mean	PI-A %CV
P1 (*P. chienii*)	84,015	2.9	84,410	2.6	82,777	3.0
P2 (*T. mairei*)	54,795	3.1	55,407	3.5	54,128	3.4
Size (Gb)	15.69		15.58		15.64	
Mean size (Gb)	15.64					

R3Q3: Visualization of clustering results

In Supplementary Figure 10, clusters are difficult to distinguish because the colors used are too similar. A revised color palette with higher contrast would substantially improve readability.

Response: We thank you for this helpful suggestion. We have revised the color palette used in Supplementary Fig. 10 to improve contrast and visual separation among clusters. The updated figure substantially improves readability and cluster distinguishability.

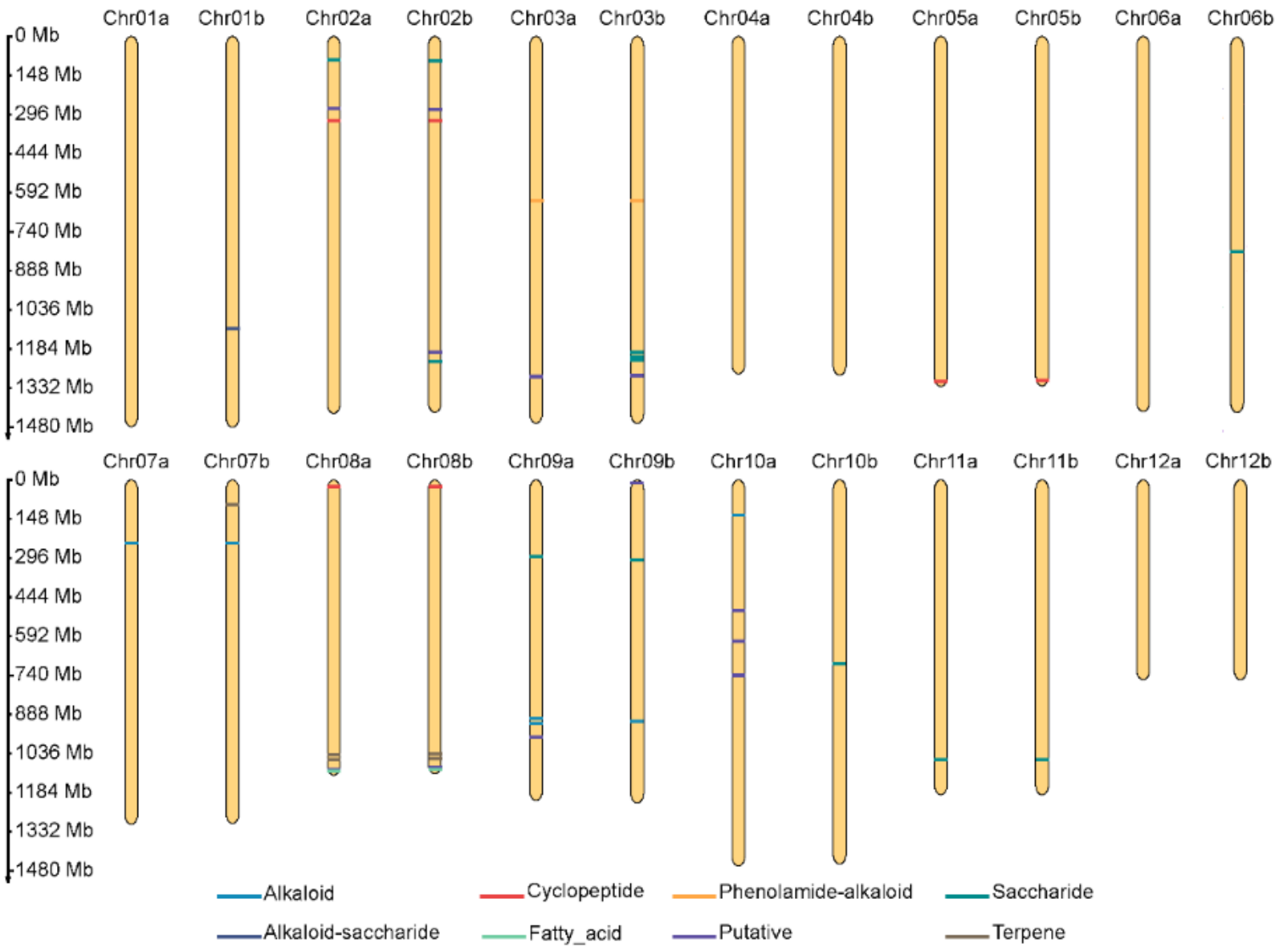

Supplementary Fig. 10 | The chromosomal distribution of secondary metabolism-related gene clusters in *P. chienii*. Color bars and labels indicate key enzyme-coding genes involved in pathways such as esters (putative), lignans, alkaloids, terpenes, and polyketides.

R3Q4: Visualization of co-expression modules

Readability, legend and explanations to interpret Supplementary Figure 11 should be improve for reader not familiar with this type of analysis. Does these colors correspond to those mentioned in Supplementary Data 3? Does the complete gene set have been clustered or only metabolic related genes? In addition, more explanation should be provided on the source of the data (108 RNA seq libraries) and plant materials? Is this

a reanalysis of previously obtained data (reference 68)? Do the plant materials originate from the same source as in this manuscript?

Response: We thank you for these valuable comments. We agree that additional explanation was needed to improve the readability and interpretability of Supplementary Fig. 11, particularly for readers less familiar with WGCNA-based co-expression analyses.

First, regarding the module visualization: we have substantially revised the figure legend for Supplementary Fig. 11. We now explicitly clarify that the colored bars beneath the dendrogram represent distinct co-expression modules and correspond exactly to the module assignments provided in Supplementary Data 3. We also clarified that the clustering analysis was performed using the TPM data for all expressed genes passing quality filtering, rather than only metabolism-related genes.

Second, regarding the source of the data and plant materials: we apologize for the incompleted description in our original manuscript. The co-expression analysis was originally performed on a highly robust, integrated dataset comprising 128 *P. chienii* transcriptomes, rather than just the 108 libraries mentioned. To provide full transparency, we have now explicitly detailed the composition of this comprehensive 128-sample dataset in the revised Methods (**lines 622-630**). It consists of three parts:

1) Transcriptome data from a population of 108 *P. chienii* individuals. This is indeed a reanalysis of the previously published dataset (Reference 68 in the original manuscript, now Reference 92 in the revised manuscript). Importantly, we have clarified that these plant materials represent diverse natural populations distinct from the individual (SQS001) sequenced in our current study.

2) Additional publicly available *P. chienii* transcriptomic data from various studies.

3) The newly generated tissue-specific transcriptomes from our current study.

All raw data were processed and clustered using a unified computational pipeline. We have updated both the Methods section and the Supplementary Fig. 11 legend to provide full transparency regarding the biological sources and composition of this dataset.

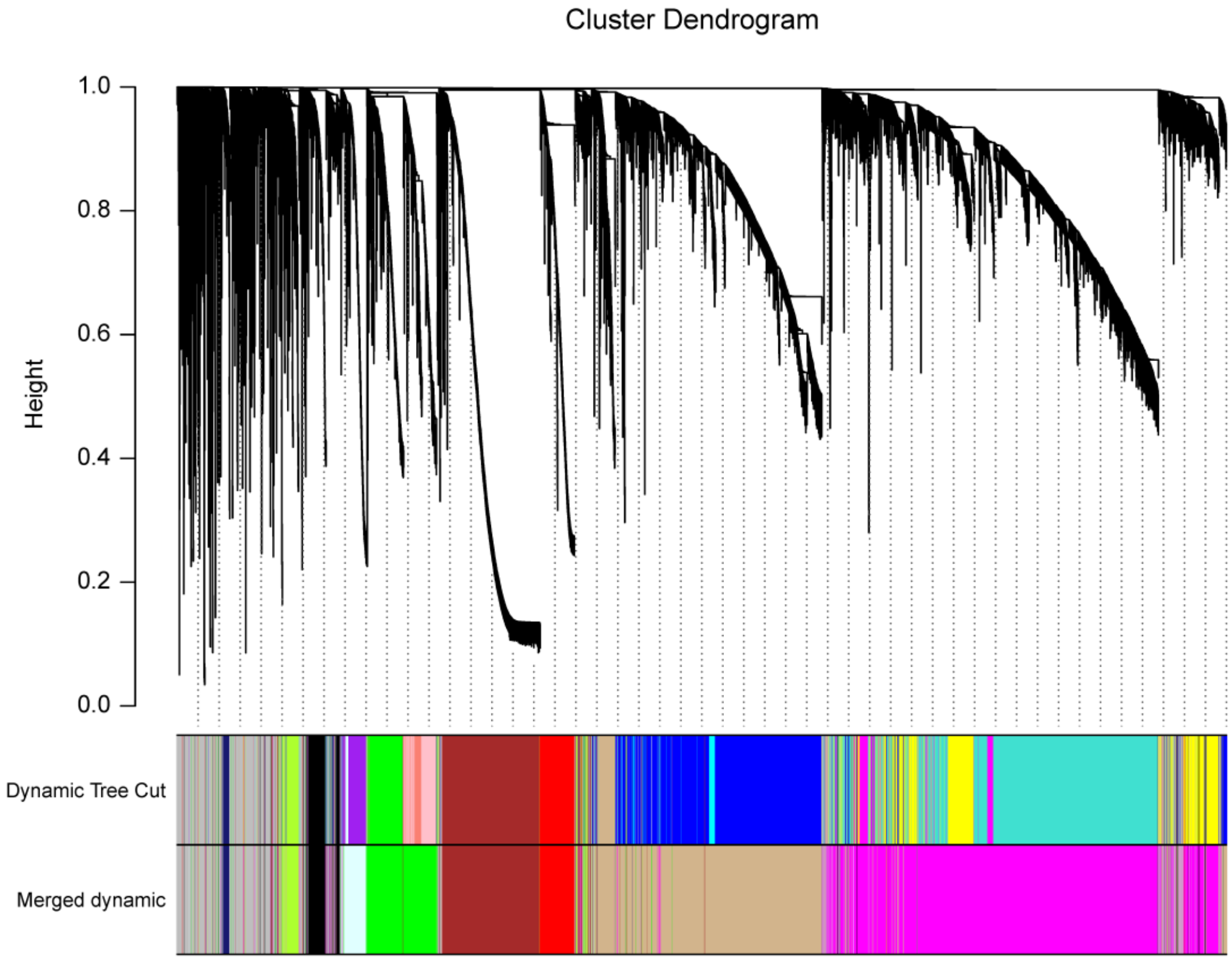

Supplementary Fig. 11 | Weighted gene co-expression network analysis (WGCNA) of *Pseudotaxus chienii* transcriptomes. The dendrogram was generated using expression profiles from RNA-seq libraries derived from multiple *P. chienii* tissues, developmental stages, and treatment conditions. All expressed genes passing quality filtering were included in the network construction and module clustering analysis. Branches group genes into distinct co-expression modules identified by topological overlap-based hierarchical clustering. Colored bars below the dendrogram represent different co-expression modules and correspond to the module assignments provided in Supplementary Data 3. The transcriptomic dataset used for WGCNA consisted of both newly generated RNA-seq data from this study and previously published datasets, which were reanalyzed under a unified computational pipeline. Scale bar, Pearson correlation distance.

Page 15, lines 622-630, "To construct a highly robust, species-wide co-expression network, we utilized an integrated dataset comprising 128 *P.chienii* transcriptomes.

This comprehensive dataset consists of three parts: (1) transcriptome data from a population of 108 *P. chienii* individuals[92] (BioProject ID: PRJNA674020), which represent diverse natural populations distinct from the individual (SQS001) sequenced in this study; (2) additional publicly available *P. chienii* transcriptomic data (BioProject ID: PRJNA630781[93]; PRJEB4922[94]; PRJNA726756[95]; PRJNA396655[96]; PRJNA498605[97]); and (3) the tissue-specific transcriptomes generated in our current study (BioProject ID: PRJCA042793). Transcripts Per Kilobase Million (TPM) data for all genes across these 128 integrated samples served as input matrix."

R3Q5: Missing overview of the metabolic pathway

A schematic representation of the complete taxane biosynthetic pathway is missing. To facilitate comprehension, I recommend adding a figure summarizing:

• the main metabolites

• the corresponding enzymatic steps

• the identified genes from *P. chienii* and/or T. marei

This could be included as supplementary material, for instance by expanding Supplementary Figure 12B.

A complete pathway would not only help for comprehension but also to confirm, or not, that the complete gene set for paclitaxel biosynthesis is present in *P. chienii*. For instance, homolog for T9ox (a 2-oxoglutarate dependent dioxygenase, McClune et al, 2025) was apparently not reported in this manuscript. If this is correct, *P. chienii* does not have a complete biosynthetic pathway for paclitaxel.

In addition, a number of putative homologs with lower sequence identity were not functionally tested, including T9αH-750C (68.7% identity), T1βH-184C (68.2%), BAPT (67.8%), T3'NBT (73%) and DBTNBT (71.9%). To argue a complete paclitaxel pathway, it would be important to test these as well.

Response: We thank you for this valuable suggestion. We agree that a comprehensive schematic overview of the taxane biosynthetic pathway would substantially improve the readability and interpretability of the manuscript.

First, regarding the pathway schematic: We expanded Supplementary Fig. 15 to include a detailed overview of the taxane/paclitaxel biosynthetic pathway. The revised figure now summarizes: i) the major taxane intermediates, ii) the corresponding enzymatic reactions, and iii) the identified homologous genes in *P. chienii* and *T. mairei*. This revised pathway map provides a clearer framework for evaluating the completeness and functional status of the paclitaxel biosynthetic pathway in *P. chienii*.

Second, regarding the presence of *T9ox* and the functional testing of downstream homologs: Following your comment, we also carefully re-examined the presence of T9ox genes in the *P. chienii* genome. We confirmed that a homolog of *T9ox* (taxane 9-oxidase) is present in the genome assembly and is now explicitly labeled in the revised Supplementary Fig. 16. In addition, we successfully cloned the corresponding candidate gene and included it in our downstream functional analyses. Furthermore, we expanded the heterologous functional characterization experiments to include several additional downstream pathway candidates with relatively lower sequence identity to known *Taxus* enzymes, including *T9ox*, *T9αH-750C*, *T1βH-184C*, *BAPT*, *T3'NBT*, and *DBTNBT*. These newly added experiments are now presented in the revised manuscript (lines 374-377, lines 474-493) and Supplementary Fig. 20.

Notably, although these homologs could be successfully cloned and expressed, none of them produced detectable catalytic products under the heterologous tobacco expression system used in this study. Together with the persistent absence of key intermediates such as baccatin III and 10-deacetylbaccatin III, these results further support our conclusion that the downstream paclitaxel biosynthetic pathway in *P. chienii* has undergone substantial functional attenuation.

Accordingly, we revised the manuscript to clarify that *P. chienii* appears to retain a structurally complete set of paclitaxel biosynthetic genes at the genomic level, but several downstream enzymatic modules exhibit severe functional degeneration. We believe these revisions substantially strengthen both the pathway overview and the mechanistic interpretation of pathway evolution in *P. chienii*.

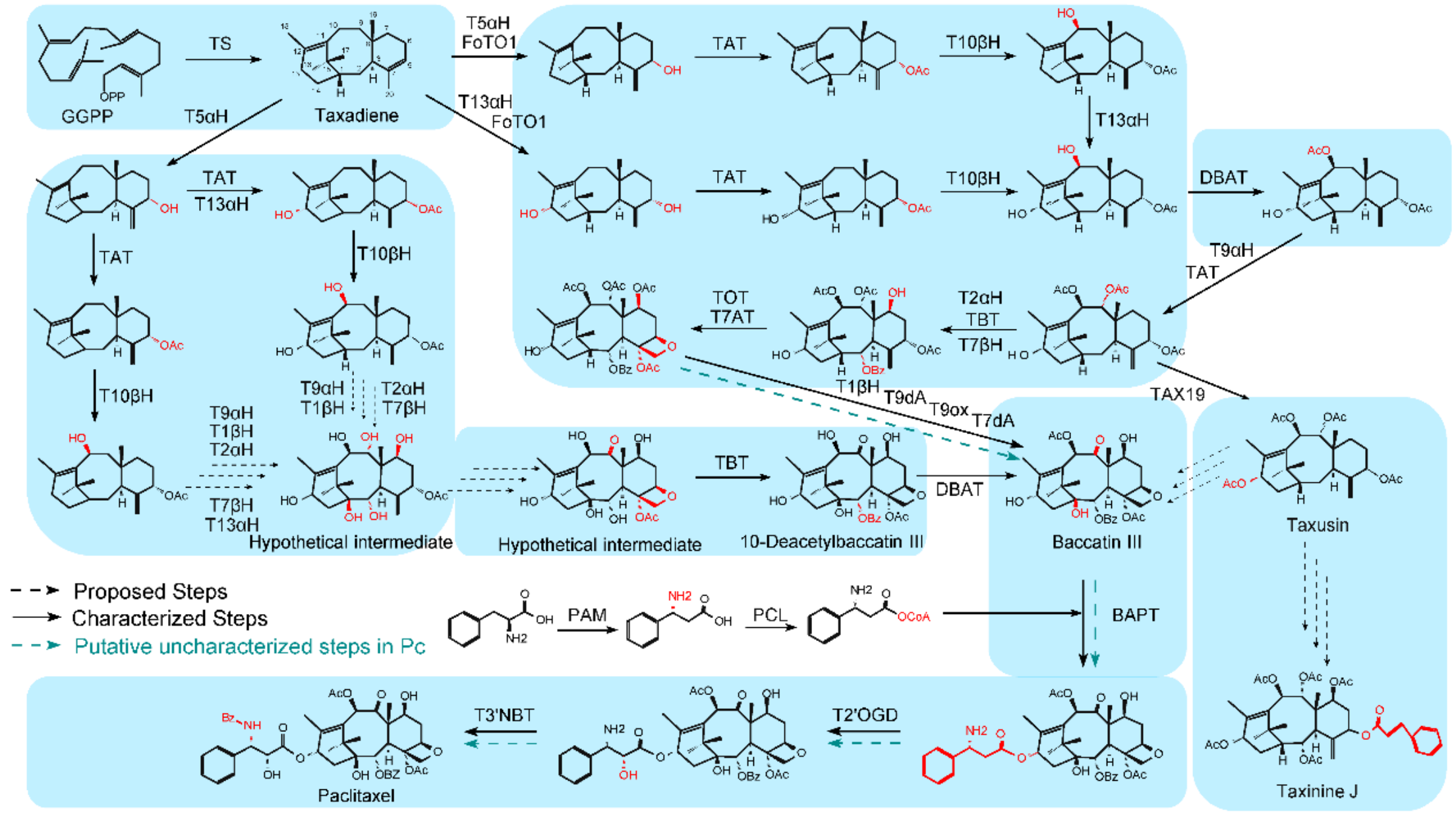

Supplementary Fig. 15 | The paclitaxel biosynthetic pathway in *Pseudotaxus chienii*. Schematic representation of the complete paclitaxel biosynthetic pathway from GGPP to paclitaxel in *P. chienii*, with predicted structures of key enzymes and catalytic steps.

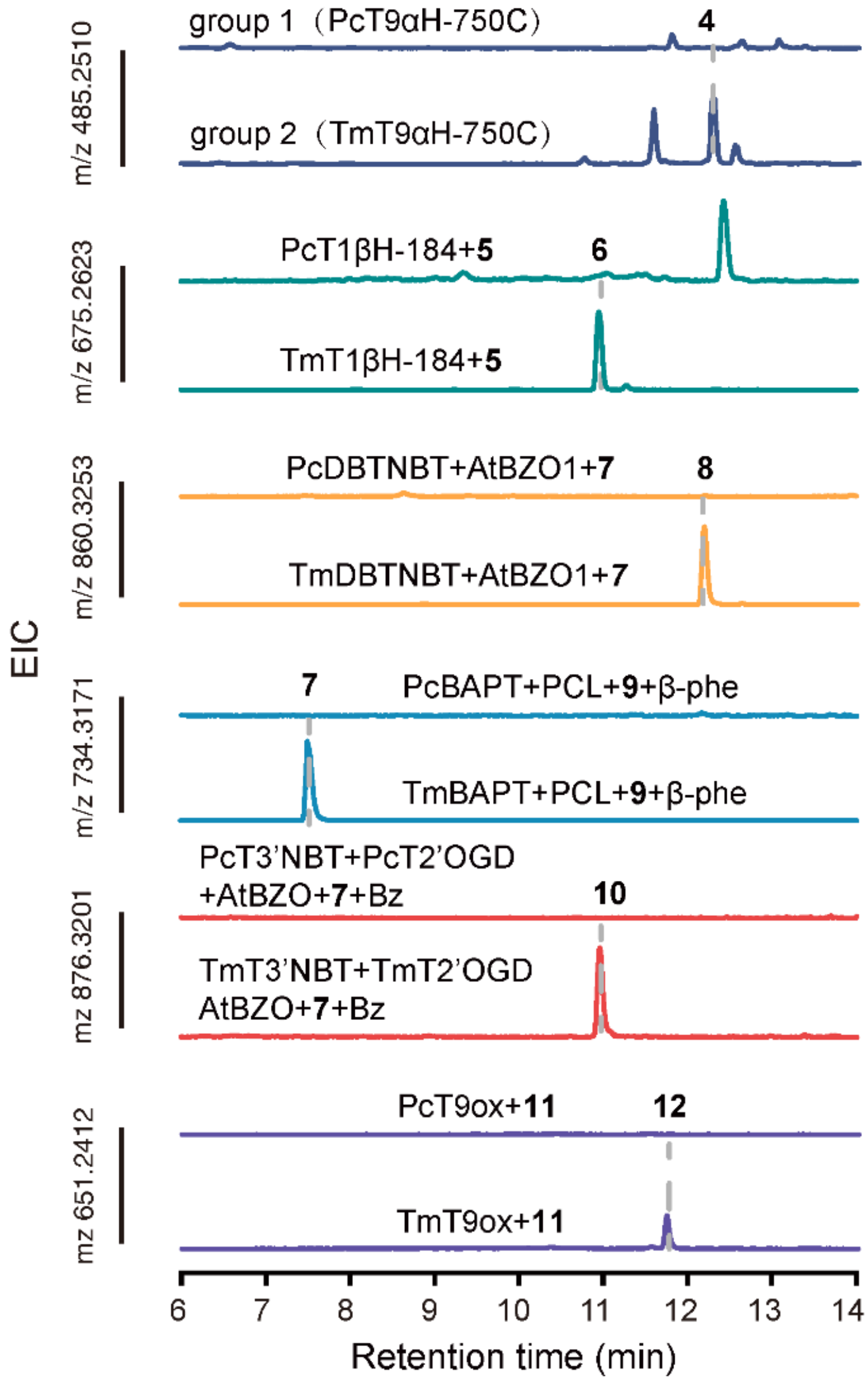

Supplementary Fig. 20 | Functional characterization of candidate paclitaxel biosynthesis genes from *Pseudotaxus chienii*. In vivo enzymatic activities of candidate genes from *P. chienii* (group 1) were assayed using a transient expression system in tobacco leaves. The corresponding orthologous genes from *T. mairei* (group 2) were used as positive controls in all assays. Extracted ion chromatograms (EICs) illustrate the production of target metabolites across different functional validation assays: De novo synthesis of 13a-deacetyltaxusin (**4**) by co-expressing *PcT9aH-750C* with upstream biosynthesis genes. Production of baccatin IV (**6**) in leaves expressing *PcT1βH-184* following the infiltration of 1-dehydroxybaccatin IV (**5**). Production of 2' -deoxypaclitaxel (**8**) in leaves co-expressing *PcDBTNBT* and *AtBZ01* with the infiltration of 3'-debenzoyl-2 -deoxypaclitaxel (**7**). Synthesis of **7** in leaves co-

expressing *PcBAPT* and *PCL* (a phenylalanine-CoA ligase with an A312G mutation from *Penicillium chrysogenum*) supplemented with baccatin I (**9**) and Β-phenylalanine (β-phe). Production of paclitaxel (**10**) in leaves expressing *PcT3'NBT*, *PcT2'OGD*, and *AtBZO1* with the infiltration of **7** and benzoic acid (Bz). Production of 13-acetylbaccatin III (**12**) in leaves expressing *PcT9ox* following the infiltration of 9-dihydro-13-acetylbaccatin III (**11**).

Page 10, lines374-377, "Moreover, despite genomic retention and high sequence conservation with *Taxus* orthologs, heterologous expression of six core downstream candidates, including *T9ox* and *BAPT*, in *Nicotiana benthamiana* failed to yield detectable pathway products, in sharp contrast to active *T. mairei* counterparts (Supplementary Fig. 20**)**."

Page 12, lines 474-493, "To systematically investigate whether this functional deficiency extends across the downstream pathway, we characterized two core late-stage functional modules. The first module contains key oxygenases involved in skeleton modification, including T9ox (65.3% sequence identity to the *Taxus* ortholog), T9αH-750C (68.7% identity), and taxane 1β-hydroxylase (T1βH-184) (68.2% identity). The second module includes terminal acyltransferases responsible for side-chain formation and attachment, namely baccatin III:3-amino-3-phenylpropanoyl transferase (BAPT) (67.8% identity), taxoid-3′-N-benzoyltransferase (T3'NBT) (73.0% identity), and 3'-N-debenzoyl-2'-deoxypaclitaxel-N-benzoyl transferase (DBTNBT) (71.9% identity). Despite genomic retention and considerable sequence conservation, none of the genes from either downstream module produced detectable pathway products in *Nicotiana benthamiana*, whereas the functional competence of the corresponding *T. mairei* orthologs remained intact (Supplementary Fig. 20).

Together with the significantly reduced catalytic efficiency of PcTOT, the absence of detectable activity across these downstream functional modules in the heterologous system strongly indicated profound functional attenuation of the terminal paclitaxel biosynthetic pathway. Although we cannot completely exclude the possibility that these

enzymes may retain weak basal activities in the presence of *P. chienii* specific co-factors or native cytochrome P450 reductases that are absent from the heterologous system, the overall heterologous in planta enzymatic profile, which characterized by catalytic activities reduced to near or below the detection threshold, is highly consistent with our endogenous targeted metabolite analyses."

R3Q6: Archipelago genomic structure and pathway organization

The reported archipelago genomic structure is highly interesting, but the figures do not clearly convey its biological implications.

It would be important to clarify whether the physical genomic organization of the archipelago follows the biosynthetic logic of the taxane biosynthetic pathway and if so, to explicitly illustrate the metabolic pathway through the lens of the archipelago structure.

If no direct correspondence exists, please discuss whether different pathway modules are nevertheless correlated with specific archipelago regions.

Response: We thank you for this insightful comment. Our analyses indicate that the physical organization of the TBA does not strictly follow the linear biochemical sequence of the canonical paclitaxel pathway. Instead, genes involved in upstream scaffold formation, oxidative tailoring, acylation, and downstream diversification are distributed across multiple syntenically conserved but physically separated genomic regions. Therefore, the TBA differs fundamentally from the compact and collinear biosynthetic gene clusters commonly observed in angiosperms.

Importantly, although no strict pathway-order collinearity was observed, we found that different archipelago regions nevertheless show functional enrichment for particular biosynthetic modules. For example, some regions are preferentially enriched for core scaffold-forming enzymes and cytochrome P450-mediated modification steps, whereas others contain downstream acyltransferases and pathway diversification-related enzymes. In addition, these dispersed modules display coordinated transcriptional behavior and shared chromatin activity patterns, supporting the concept

that the TBA functions as an integrated regulatory architecture despite extensive genomic separation.

Accordingly, we revised the Results (**lines 223-237**) and Discussion (**lines 404-418**) to clarify that the TBA represents a modular and distributed biosynthetic organization rather than a conventional compact metabolic assembly. We further discuss how this organization may provide evolutionary flexibility for pathway duplication, diversification, regulatory rewiring, and partial functional degeneration during *Taxaceae* evolution. The detailed description as follow:

Page 6, lines 223-237, "To quantitatively validate the functional integration of this dispersed genomic archipelago, we combined chromatin architecture profiles derived from Hi-C analysis (using the Pca haplotype as a representative model) with Pearson correlation analyses of a comprehensive 128-sample transcriptomic dataset. Hi-C profiles revealed that the entire TBA region is embedded within the transcriptionally active A compartment[27,28], providing a globally favorable environment for gene expression. Notably, the three gene clusters are spatially segregated into distinct Topologically Associating Domains (TADs)[29,30], which further subdivide into localized sub-TADs (Supplementary Fig. 12a). This hierarchical division closely aligns with functional boundaries; for example, the TAD encompassing TS1C exclusively harbors core metabolic genes, effectively demarcating the functional domain by excluding a non-metabolic gene at its left border. Although separated into independent TADs, metabolic genes from the three clusters and these dispersed core enzymes form a highly coordinated co-expression network, with the majority of exhibiting Pearson correlation coefficients (r) ≥ 0.9 (Supplementary Fig. 12b). Collectively, these findings indicate that the functional integrity of the TBA is determined by regulatory coordination and active chromatin occupancy, rather than strictly by linear physical proximity."

Page 10-11, lines 404-418, "To address this challenge, we introduced the TBA, which illustrates a striking evolutionary adaptation: pathway coordination within these large phylogenetic lineages operates independently of physical gene proximity. Defined by

functionally linked yet spatially dispersed biosynthetic gene clusters, the TBA represents a departure from the canonical model of compact BGCs[31], raising a key question of how functional coordination is maintained across large genomic regions. Traditional models generally invoke direct chromatin looping to account for long-range co-expression, whereas our findings reveal a distinct spatial regulatory paradigm in expanded gymnosperm genomes. Although spatially segregated into distinct TADs, the three TBA clusters maintain robust regulatory synchrony without relying on direct physical contact. Instead, this coordinated expression is driven by spatial co-localization within the transcriptionally active A compartment, coupled with unified regulation by trans-regulatory networks. This insight refines the evolutionary understanding of specialized metabolism. In large expanded genomes, the integrity of biosynthetic pathways does not require linear contiguity or complex 3D chromatin folding. Rather, functional modules can be isolated via precise topological insulation and synchronously modulated by common global regulatory signals[48]."

R3Q7: Clarification of paclitaxel detection and quantification in *P. chienii*

The presence of paclitaxel in Pseudotaxus was mentioned to be controversial in the literature. The authors claim to detect and quantify paclitaxel in both leaves and bark of *Pseudotaxus chienii* in this report. Figure 3 report between 10-5 and 10-6 µg/mg of paclitaxel in *P. chienii* bark, so in the 1 to 10 ng per g range. These levels detected are extremely low and the methodology however not fully reported (sMRM protocol), and thus hard to evaluate that methods are adequate for trace detection. Detecting trace level of paclitaxel in complex matrix may be highly challenging. Minimum quality control would require matrix spike with similar level of paclitaxel d5 (the amount reported in M&M is much higher), standard addition (ie 2 or 3 spikes eg +1, +2 + 5 ng/g equivalent) into the "same" extract to show the signal scales linearly and the paclitaxel/paclitaxel d5 ratio behaves properly. The sMRM method should relies on two MRM transitions (quantifier and qualifier) and both must be present at the same RT, with a stable peak shape. Qualifier/quantifier ratio should match standards (typical acceptance window: + - 20 to 30% relative). A typical chromatogram for sMRM detection in bark extract

should be presented to assess the background/signal ratio. LOD and LOQ reported as well. In addition, note that carryover (sticking to glassware, metal, lines) is a recognized analytical risk in trace analysis by LC-MS/MS. Best practice in trace analysis is to demonstrate carryover removal as part of validation.

The error bars for taxane levels in Figure 3 are very small, likely corresponding to a technical repeat. Biological repeats should be performed.

Response: We sincerely thank you for the professional and constructive comments regarding our trace detection methodology. To address your concerns regarding data reliability, we have expanded our validation using an ultra-sensitive SCIEX 7500 triple quadrupole mass spectrometer and comprehensively revised the methodology section (**lines 712-762**).

First, we would-like to clarify a typographical error in our initial manuscript: the term "sMRM" (scheduled-MRM) was used by mistake; we actually employed the standard MRM (multiple-reaction monitoring) mode.

To ensure full transparency and address the concern that the methods were difficult to evaluate, we have thoroughly updated- the "LC-MS analysis" section. It now details the chromatographic conditions, gradient programs, and all ion source parameters for both mass spectrometry platforms used in this study.

Regarding the specificity of trace detection (qualifier/quantifier transitions), we opted for an approach that provides even higher confidence to eliminate matrix-derived false positives. Using the linear ion trap functionality of the SCIEX 5500 QTRAP, we acquired a complete MS/MS fragmentation spectrum (EPI scan) of the trace paclitaxel peak (Figure 3f). This full spectrum matches the authentic standard, providing unambiguous, "fingerprint-level" structural confirmation of the target molecule.

We are especially grateful for your meticulous review of the reported concentrations. Prompted by your comment regarding the 1 to 10 ng/g range, we re-evaluated our raw data and discovered a unit conversion error in the previous draft that inadvertently reduced the final concentration order of magnitude by 10-fold. The actual paclitaxel content in the *P. chienii* bark is between 10^{-4} and 10^{-5} μg/mg (approximately

26 - 89 ng/g). To rectify this and facilitate intuitive comparisons across samples, we have unified all metabolite concentration units to ng/g DW throughout the manuscript and figures. Figure 3b has been replotted using the corrected data and unified units.

To address the need for matrix spiking and linear scaling, our quantitative methodology strictly adheres to the group standard "Quantitative detection guidelines for taxanes in *Taxus* species" (T/CI 1115-2025). For samples analyzed on the ultra-sensitive 7500 system, a stable isotope internal standard (paclitaxel-d5, 20 ng/mL) was explicitly spiked into each sample prior to extraction. This dynamically compensates for extraction losses and complex matrix effects, achieving a correction equivalent to standard addition. Calibration curves (0.5-1000 ng/mL) were consistently spiked with the internal standard, and quantification was performed using internal-standard-normalization (1/x2 weighting) to ensure linear scaling. Regarding the Limit of-Quantification (LOQ), the lowest point of our calibration curve is 0.5 ng/mL, which corresponds to a quantification limit far below the actual trace levels of paclitaxel detected in our samples (approximately 26 - 89 ng/g). This demonstrates that our methodology is highly sensitive and- fully adequate for this trace analysis.

To eliminate platform-specific biases and test reproducibility, we completely re-analyzed the original archived samples for all four targeted metabolites using the higher-sensitivity SCIEX 7500 mass spectrometer. Furthermore, we expanded our dataset by collecting and analyzing 17 new spatiotemporal samples across different seasons and geographically isolated habitats using the 7500 platform. As detailed in the revised manuscript, Figure 3f displays the MS/MS structural confirmation obtained from the 5500 QTRAP system, whereas the comprehensive quantitative results from the 7500 platform are presented in Supplementary Fig. 18. Notably, the 7500 platform results include the successful re-analysis of the exact same *P. chienii* leaf sample shown in Supplementary Fig. 18a. Both platforms independently verified the presence of paclitaxel in the positive samples, confirming that the trace accumulation is a verifiable biological event.

We completely agree that carryover is a recognized analytical risk in trace LC-MS/MS analysis. To actively mitigate this, our chromatographic gradient included

astringent wash step (holding at 95% mobile phase B) to ensure complete carryover removal before: the next injection. Additionally, quality-control pools were systematically injected every 10 samples to monitor system stability and verify the absolute absence of background interference. The excellent system stability (intra-day RSDs < 14.8%) confirms that residues were effectively eliminated.

Finally, regarding the error bars in Figure 3b: these represent true independent biological replicates (n = 4-6), not technical replicates. The narrow appearance of the error bars is an artifact of the logarithmic (Log^{10}) transformation on the Y-axis. This scaling was strictly necessary to display the massive, order-of-magnitude differences in taxane content between *Taxus* and *Pseudotaxus* samples. We have updated the Figure3 legend to explicitly clarify this visual compression effect.

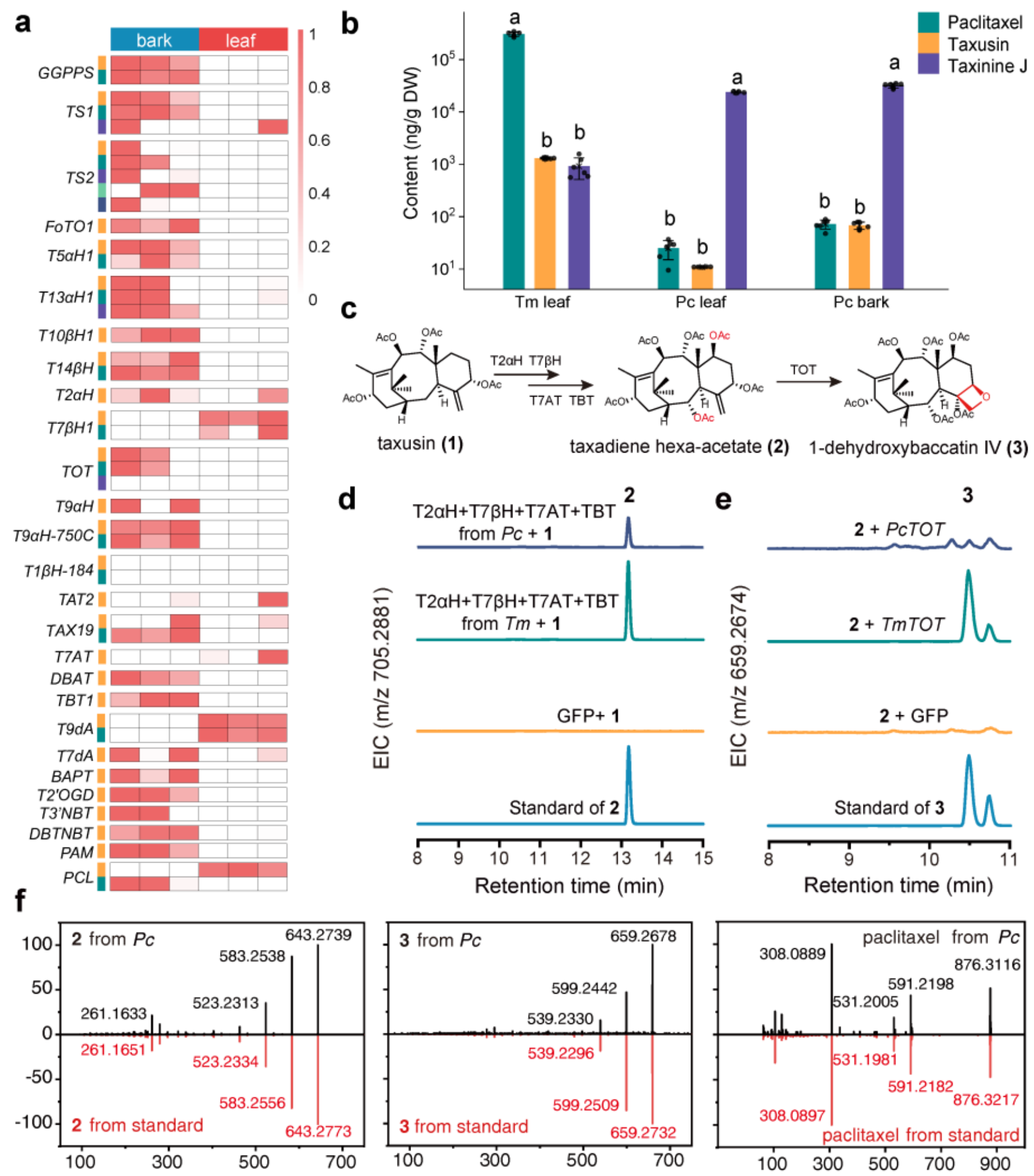

Fig. 3 | Identification of the paclitaxel biosynthetic pathway in *Pseudotaxus chienii* . **a** Heatmaps display the expression levels of corresponding biosynthetic genes in *P. chienii* leaf and *P. chienii* bark tissues. Different colors represent different gene copies in *P. chienii* hap1. **b** Targeted LC-MS profiles of key taxane metabolites in Pc leaf, Pc bark, and *T. mairei* leaf (Tm leaf) (n= 4-6). Different lowercase letters indicate a significant difference ($P < 0.01$) based on one-way ANOVA with two-tailed Duncan test. Error bars represent means ± SD of independent biological replicates. Note: The Y-axis is plotted using a Log10 scale to accommodate the magnitude of concentration differences between species, which visually compresses the error bars representing biological variability. **c** Schematic of T2αH, T7βH, T7AT, and TBT convert taxusin (**1**) to taxadiene hexa-acetate (**2**), and the schematic of TOT convert taxadiene hexa-acetate (**2**) to 1-dehydroxybaccatin IV (**3**). **d, e** EICs of expected products when the T2αH, T7βH, T7AT, TBT (**d**) and TOT (**e**) from *P. chienii* or *T. mairei* is expressed in *Nicotiana benthamiana*. **f** MS/MS spectra of taxadiene hexa-acetate (**2**), 1-dehydroxybaccatin IV (**3**) and paclitaxel produced in tobacco (black) and its standard (red).

Page 18-19, lines 712-762, "Targeted metabolite detection and trace quantification of taxane compounds were strictly performed in accordance with the authoritative group standard "Quantitative detection guidelines for taxanes in *Taxus* species" (T/CI 1115-2025)[109]. The quantitative analyses were performed on a SCIEX 5500 QTRAP® triple-quadrupole/linear-ion-trap hybrid mass spectrometer equipped with a Turbo-V™ ion source and a Shimadzu LC-2030 UHPLC system. For the 5500 system, chromatographic separation was achieved on a Waters ACQUITY UPLC BEH T3 column (2.1 × 100 mm, 1.8-μm) maintained at 450 °C. Mobile phases consisted of (A) 0.1 % formic acid in water and (B) 0.1 % formic acid in acetonitrile delivered at 0.4 mL min^{-1}. A 10-min gradient was programmed as follows: 0 – 0.5 min 20 % B, 0.5 – 6.5 min 20 → 95 % B, 6.5 – 7.5 min 95 % B (stringent wash to ensure complete carryover removal), 7.5 – 7.6 min 95 → 20 % B, followed by 2.4 min re-

equilibration. Injection volume was 5 μL and samples were maintained at 8°C in the autosampler.

To rigorously cross-validate the trace detection of paclitaxel at extreme low abundance, independent targeted analyses were also conducted on a higher-sensitivity SCIEX 7500 Triple Quad mass spectrometer coupled with an ExionLCTM system (AB SCIEX, USA), which was equipped with a heated electrospray ionization (HESI) probe. For the 7500 system, chromatographic separation was performed on a Waters ACQUITY UPLC HSS T3 column (2.1 × 100 mm, 1.8 μm) maintained at 40 °C. The mobile phases consisted of (A) 4 mM ammonium acetate in water and (B) 4 mM ammonium acetate in methanol, delivered at a flow rate of 0.3 mL min-1. A 20-min gradient program was applied as follows: 0 - 2.5 min, 10 → 15% B; 2.5 - 5 min, 15 → 40% B; 5 - 7 min, 40 → 70% B; 7 - 8 min, 70% B; 8 - 11min, 70 → 80% B; 11 - 13 min, 80 → 100% B; 13 - 16 min, 100% B;16 -16.5 min, 100 → 10% B; followed by 3.5 min of re-equilibration. The injection volume was 2 μL, and the autosampler temperature was maintained at 8 °C.

The 5500 mass spectrometer was operated in positive electrospray ionization (ESI^+) mode with multiple-reaction monitoring (MRM) using compound-specific transitions optimized via direct infusion of authentic standards. Quantification on the 5500 system was established using the external standard method. To unambiguously exclude matrix-derived false positives, enhanced product ion (EPI) scans were triggered on the 5500 QTRAP system to acquire full tandem mass spectrometry (MS/MS) fragmentation spectra, utilizing its linear-ion-trap functionality to structurally confirm the exact identity of trace paclitaxel against authentic standards.

In parallel, the ultra-sensitive 7500 system was dedicated to trace quantification of low-abundance samples utilizing the stable-isotope (paclitaxel-d_5) internal standard method in multiple reaction monitoring (MRM) mode under positive ionization, with ion transitions optimized using chemical standards. Source parameters for the 5500 system were: curtain gas 25 psi, ion-spray voltage 5.5 kV, temperature 500 °C, nebulizer gas (GS1) 50 psi, and heater gas (GS2) 50 psi. Source parameters for the 7500 system were: curtain gas 40 psi, ion-spray voltage 2.5 kV, temperature 400 °C,

nebulizer gas (GS1) 35 psi, and heater gas (GS2) 60 psi. Compound-dependent parameters (including declustering potential, collision energy, and cell-exit potential) were automatically optimized with Analyst® 1.7 and SCIEX OS software.

Calibration curves (0.5 – 1000 ng mL^{-1}) of taxinine J (CAS: 18457-46-0), taxusin (CAS: 19605-80-2), Baccatin III (CAS: 27548-93-2) and paclitaxel (CAS: 33069-62-4) standards, as well as other target metabolites analyzed in Nicotiana benthamiana leaves, were prepared by serial dilution of authentic standards in 80% methanol to match the sample solvent matrix. For the 7500 system calibration, the standard solutions were consistency spiked with 20 ng mL^{-1} paclitaxel-d5 to match the internal standard concentration in the samples. Quality-control pools (low, mid, high) were injected every 10 samples to actively monitor system stability and verify the absence of background interference; intra-day RSDs were < 14.8 %. Data acquisition and quantification were performed with Analyst 1.7 and MultiQuant 3.0 softwares for the 5500 system, applying external standard calibration. For the 7500 system, SCIEX OS software was applied for taxinine J, taxusin, Baccatin Ⅲ and paclitaxel identification and peak integration, utilizing internal-standard normalization with a $1/x^2$ weighting factor."

R3Q8: Clarification of preliminary metabolic profiling versus targeted LC–MS

The distinction between preliminary metabolic profiling and targeted LC–MS analysis in *P. chienii* is not clearly defined. Only LC–MS experiments are described in the Materials and Methods section, making it difficult to understand what is meant by "preliminary metabolic profiling." Please clarify whether this term refers to an initial metabolite annotation or identification step using computational prediction tools (e.g. SIRIUS or related software), or to a distinct experimental approach that is not fully described.

Response: Thank you for your question. We sincerely apologize that our inaccurate description caused confusion. All analyses performed in our study were targeted LC–

MS analyses and did not include preliminary metabolic profiling. We have corrected this in the manuscript:

Page 8, line 304-308, “To corroborate the transcriptomic findings and resolve the controversy regarding paclitaxel presence, preliminary targeted quantification of metabolites from *P. chienii* leaf (SQS001, April 2024) was performed using a SCIEX 5500 QTRAP LC-MS/MS system and a Thermo Scientific TSQ 9000 triple-quadrupole mass spectrometer coupled with a Trace 1310 gas chromatograph for GC-MS/MS detection. A total of over 41 taxane metabolites were identified across the two detection platforms (Supplementary Fig. 17a, b).”

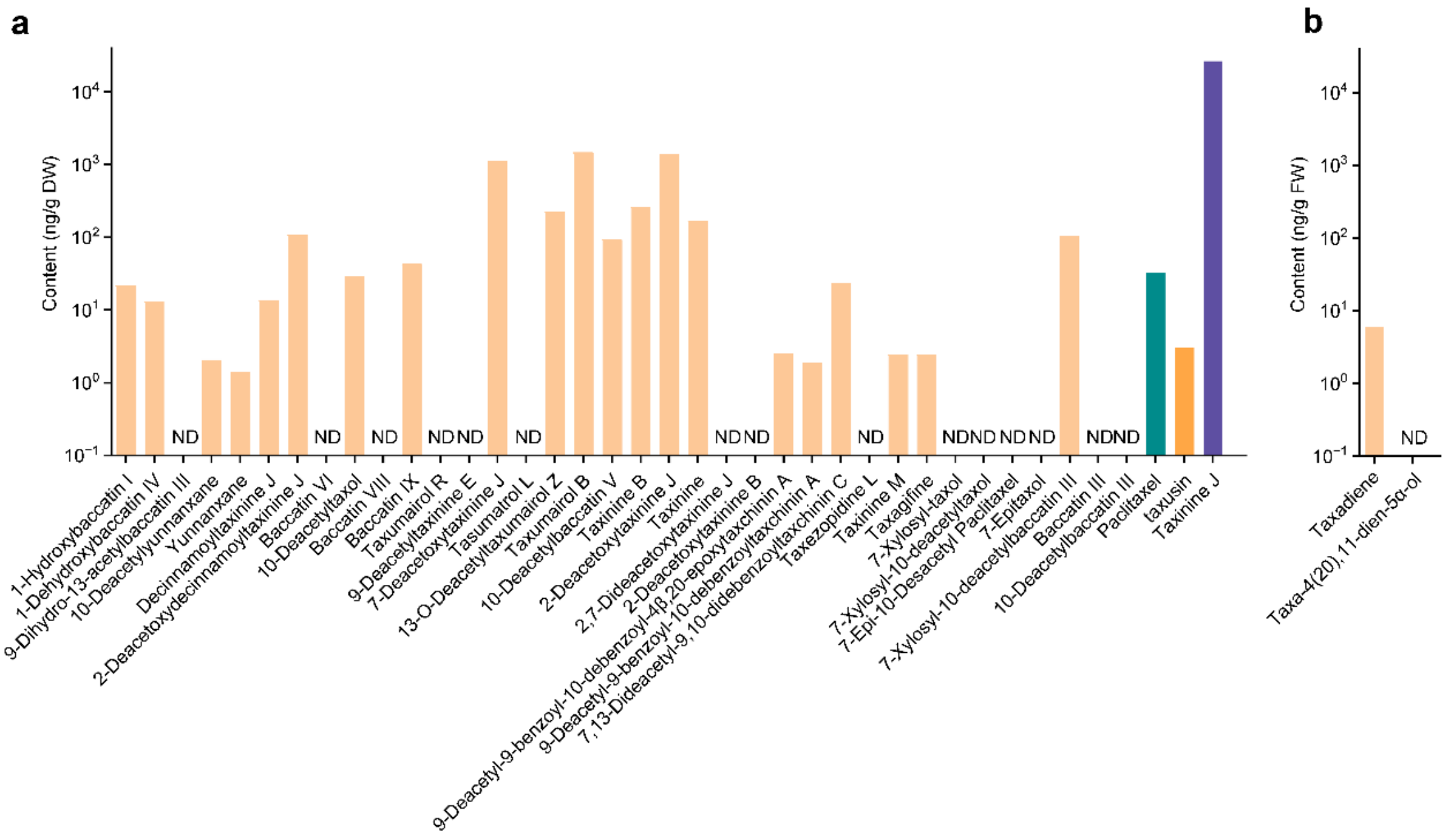

Supplementary Fig. 17 | Major taxanes in *Pseudotaxus chienii* leaf. a Content of major taxane expressed as ng/g dry weight (DW). **b** Contents of selected toxoids determined on fresh weight basis (ng/g FW)**.** Metabolites that were undetected in the assay were indicated by “ND” (Not Detected).

R3Q9: Discussion in relation to Wang et al. (2025) and implications for chemical diversity

Given that the conclusions of the present study differ from those reported by Wang et al. (2025) regarding the completeness of the taxane biosynthetic pathway in *P.*

chienii, the authors should explicitly discuss and contextualize these discrepancies. In particular, the discussion should address how differences in genome assembly strategy and resolution (chromosome-scale versus haplotype-resolved assemblies) may have led to divergent biological interpretations. In addition, the authors are encouraged to consider how haplotypic and genetic diversity may contribute to chemical diversity and the existence of distinct chemotypes in *P. chienii*, potentially shaped by genotype × environment interactions, and to discuss the implications of this variability for taxane biosynthesis and pathway reconstruction. In this manuscript, a major focus is the question of whether the taxane biosynthetic pathway is complete. Unless this issue can be clearly resolved, the authors may consider redirecting their discussion toward a broader perspective on the evolution of taxanes biosynthesis.

Response: We sincerely thank you for this insightful comment. We completely agree with your perspective and have revised the Discussion section to explicitly address the discrepancies with Wang et al. (2025), to contextualize the role of genotype-by-environment (G × E) interactions in shaping chemotype diversity, and to elaborate on the broader evolutionary implications of our findings.

First, regarding the discrepancies with Wang et al. (2025): As detailed in the revised Discussion **(lines 440-473)**, our haplotype-resolved genome assembly provides a more comprehensive view of the structural complexity of the *P. chienii* genome compared to the consensus chromosome-scale assembly employed by Wang et al. (2025). This higher-resolution approach enabled us to unequivocally demonstrate that the complete genetic architecture for the taxane biosynthetic pathway is structurally intact and organized into a novel "Taxane Biosynthetic Archipelago" (TBA). The conclusion of an incomplete pathway by Wang et al. likely stems from the inherent limitations of a consensus assembly in accurately reconstructing complex, heterozygous regions, which can lead to the collapse or misrepresentation of allelic variation and gene copy number.

Furthermore, we agree that haplotypic and genetic diversity are key drivers of chemical diversity. As newly discussed in **lines 426-439**, our assembly revealed

significant structural differences between haplotypes, such as an almost two-fold difference in *TS2* gene copy number. This allelic variation, in conjunction with specific G × E interactions, can give rise to distinct chemotypes. This is evident in our study: paclitaxel production was restricted to trace levels in only one individual under specific conditions, while the alternative product, taxinine J, was consistently the dominant metabolite. This indicates that the functional output of the pathway is not merely a matter of gene presence or absence, but is a dynamic trait modulated by underlying genetic diversity and environmental context.

Finally, in light of these findings and your excellent suggestion, we have reframed our discussion towards a broader evolutionary perspective (**lines 506-521**). We propose that the TBA in *P. chienii* represents a case of evolutionary degeneration: while the ancestral genomic architecture has been preserved under purifying selection, the terminal steps of the pathway have undergone substantial functional degradation. This hypothesis is strongly supported by our downstream enzyme activity assays. Although upstream and midstream enzymes (e.g., T2αH, T7βH, T7AT, TBT) exhibited catalytic activities comparable to their homologs in *T. mairei*, critical bottlenecks have emerged in the downstream segment. Specifically, the catalytic efficiency of *PcTOT* was significantly reduced. More importantly, the cytochrome P450 oxygenase responsible for core scaffold modification (e.g., T9ox) and the terminal acyltransferase responsible for side-chain formation (e.g., BAPT) both failed to exhibit detectable catalytic activity in our heterologous expression system. This severe functional attenuation has rerouted metabolic flux, favoring the accumulation of the alternative branch product, taxinine J, and relegating complete paclitaxel biosynthesis to a highly conditional, residual capacity dependent on specific G × E interactions. This refined model offers a new paradigm for understanding the metabolic evolution of complex pathways in non-model, highly heterozygous species.

Page 11-12, lines 440-473, "Resolving the historical debate surrounding paclitaxel biosynthesis in *P. chienii* requires addressing not only contradictory metabolic evidence, but also divergent genomic interpretations reported in recent studies. For instance,

Wang et al. (2025)[16] proposed that the pathway terminates prior to 10-deacetylbaccatin III, a conclusion likely constrained by the limitations of consensus chromosome-scale genome assemblies. In contrast, our haplotype-resolved genome demonstrates that the complete genetic architecture underlying paclitaxel biosynthetic pathway, including the core TBA and other dispersed essential genes, remains structurally intact in *P. chienii*. These results further highlighted the critical importance of haplotype-aware genome assemblies for accurately reconstructing complex specialized metabolic pathways in highly heterozygous plant genomes. However, the observed discrepancies between this genomic retention and the restricted biochemical output suggest a dynamic evolutionary trajectory rather than a contradiction. While our phylogenomic analysis indicates that the complete pathway was step-wise assembled and achieved functional integrity in the common ancestor of *Taxus* and *Pseudotaxus*, this ancient structural retention does not equate to modern functional execution. Following its divergence from *Taxus*, the terminal pathway in the *P. chienii* lineage appears to have undergone significant evolutionary functional degradation. Notably, the paclitaxel content in *P. chienii* leaves is over four orders of magnitude lower than that typically observed in *T. mairei*. Crucially, our findings reveal that the capacity for paclitaxel biosynthesis outside the *Taxus* genus cannot be simply reduced to a binary question of gene presence or absence. Rather, it represents a clear case of evolutionary functional degeneration. Although the ancestral archipelago architecture has been faithfully retained and the complete set of orthologous pathway genes displays low Ka/Ks ratios indicative of sustained purifying selection[35] and has been robustly preserved to cope with extensive genome expansion, such structural conservation does not necessarily guarantee full functional integrity. Indeed, paclitaxel could only be detected in rare instances: specifically, in a single natural sample (SQS001) collected in April 2024 (unambiguously confirmed by MS/MS spectra; Fig. 3f), together with only one positive case observed among all independently MeJA-induced branches in 2026 (Supplementary Fig. 18). These results suggest that the complete paclitaxel biosynthetic capacity is still retained in *P. chienii*, but exists in a latent rather than constitutively active state. Rather than an experimental artifact, this low induction reproducibility

underscores that complete biosynthesis is an ultra-rare, stochastic event tightly gated by intricate G × E interactions. Consistent with this interpretation, the functional assays showed that the terminal steps of the pathway, such as PcTOT, have undergone substantial evolutionary degradation. Consequently, this highly conditional and rare activation elegantly may explain the historical controversy regarding the inconsistent detection of paclitaxel in *P. chienii* across previous studies[14-16]."

Page 11, lines 426-439, "…While determining the direct metabolic consequence of this specific allelic expansion is technically impractical within the blended diploid metabolome, our promoter analyses provide robust alternative evidence for its functional role. These analyses firmly support an adaptive gene dosage strategy[49]: TS2 gene expansion allows *P. chienii* to enhance JA-dependent chemical defense upon stress, effectively balancing defensive capacity and metabolic expenditure. Furthermore, such underlying genetic variation interacts with environmental changes to shape distinct chemotypes, underscoring the need to account for G × E interactions in exploring metabolic diversification[50]. Given the endangered status of *P. chienii*, these findings carry important implications for the development of more effective conservation strategies to protect its distinctive taxane biosynthetic capability. Rather than focusing exclusively on increasing population sizes, in situ conservation efforts should prioritize the protection of heterogeneous microhabitats so as to preserve G × E interactions that drive chemical diversity. At the same time, ex situ germplasm collections can utilize the haplotype-resolved markers generated in this study to capture and safeguard the full range of chemotypes along with the species' underlying genetic architecture."

Page 13, line 506-521, "Ultimately, the haplotype-resolved genome of the endangered *P. chienii* provides a valuable new framework for understanding how complex specialized metabolism evolves in massively expanded plant genomes. By introducing the TBA, we extend the classical concept of metabolic gene clusters, showing that within such repeat-rich gymnosperm genomes, pathway coordination can be achieved

independently of physical gene proximity through localization within shared transcriptionally permissive chromatin compartments and trans-acting regulatory networks. Importantly, the combined use of structural genomics, spatiotemporal metabolite profiling, and heterologous in planta functional assays has resolved the long-standing debate over whether paclitaxel can be produced outside the *Taxus* genus. Our results point to a more subtle pattern of metabolic evolution: although the ancestral genomic architecture has been largely retained amid extreme genome expansion, pronounced functional degeneration at the later catalytic steps has constrained downstream pathway progression, favoring the accumulation of alternative branch products such as taxinine J, its various structurally related derivatives (Supplementary Fig. 17), and discernible levels of taxusin across different populations. These findings thus refine our understanding of taxane diversification and offer a critical genomic foundation for both the conservation of Taxaceae and the engineered production of high-value taxane."

R3Q10: line 82: supplementary table 2 not provided

Response: We sincerely apologize for our oversight. The missing Supplementary Tab. 2 (now Supplementary Tab. 1) have now been added to the revised supplemental files. Corresponding references in the main text have also been checked and updated to ensure consistency and accessibility.

R3Q11: line 169: supplementary figure 10, not 10a, b?

Response: Thank you for pointing this out. We have revised this figure reference and carefully checked the entire manuscript to ensure that all figure and data citations are consistent with the descriptions in the Results section.

Page 6, lines 205-206, "…identifying 21 candidate biosynthetic gene clusters (BGCs) in Pca and 25 in Pcb (Supplementary Fig. 10, Supplementary Data 1, 2)."

R3Q12: figure 2 b and c, the cluster appears to have different names in 2b and 2c. Please clarify.

Reviewing by Pr B.St-Pierre and Dr M. Durand

Response: Thank you for pointing this out. We have revised Figure 2 to ensure consistent cluster names.

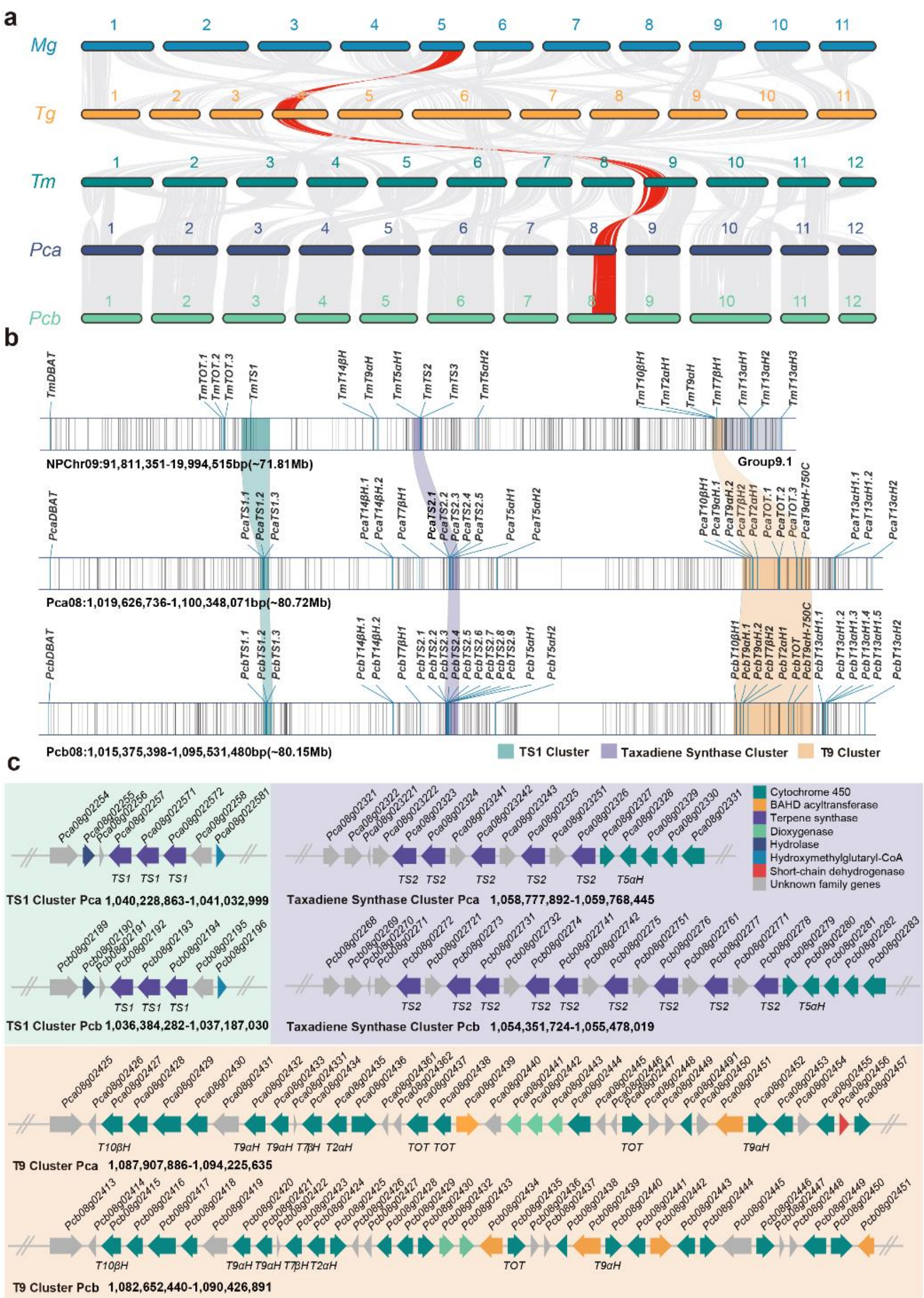

Fig. 2 | Genomic organization and structural features of Taxane Biosynthetic Archipelago (TBA) in *Pseudotaxus chienii*. a Macrosynteny among three *Taxaceae* species and the *Cupressaceae* outgroup *M. glyptostroboides* (Mg) highlights a conserved region containing three biosynthetic gene clusters (BGCs) and colinear upstream genes, including *TS*, *T5αH*, *T10αH* and *T13αH*. Tg, *T.grandis*; Tm, *T.mairei*; Pca/b, *P. chienii* hap1/hap2. **b** Colinear arrangement of paclitaxel biosynthetic genes within the TBA region. Genes involved in the paclitaxel biosynthetic pathway are shown in red. **c** Organization of three paclitaxel-related BGCs on *P. chienii* chromosome Pca08. Genes are color-coded by enzymatic function, including terpene synthases, cytochrome P450s and downstream tailoring enzymes involved in taxane modification.

Finally, we sincerely thank Prof. B. St-Pierre and Dr. M. Durand for your recognition of our work, and for providing constructive comments and valuable suggestions.

Reviewer #4:

I co-reviewed this manuscript with one of the reviewers who provided the listed reports. This is part of the Nature Communications initiative to facilitate training in peer review and to provide appropriate recognition for Early Career Researchers who co-review manuscripts.

Response: We thank you for giving us valuable suggestions and comments.

Response to REVIEWER COMMENTS:

Reviewer #1:

R1Q1: I am completely satisfied with the·authors’ revisions. I have no further comments.

Response: We sincerely thank you for your time and expertise, and we grateful for your positive assessment of our revised manuscript. Your constructive feedback in the previous round was invaluable and has led to substantial improvements in our work.

Reviewer #2:

R2Q1: The revision has addressed most of my concerns. I have no more question.

Response: We deeply appreciate your thorough review and endorsement of our revisions. Your insightful suggestions throughout this process have been instrumental in refining the manuscript, and we are grateful for your guidance.

Reviewer #3:

R3Q1: In the manuscript entitled “Archipelago architecture and evolution of paclitaxel biosynthesis revealed by the Pseudotaxus chienii haplotype-resolved genome”, the authors have provided a revised version that addresses many of the concerns raised during the previous review round. Overall, most of the questions raised by Reviewers #3 and #4 have been satisfactorily addressed.

Response: We sincerely thank you for carefully reassessing our manuscript and for recognizing the improvements made in the previous revision.

R3Q2: However, we still have a major concern regarding the conclusion that Pseudotaxus chienii retains the capacity to synthesize paclitaxel.

In my view, the new data provided by the authors further strengthen the alternative interpretation that *P. chienii* may have largely lost, or no longer possess, a functional paclitaxel biosynthetic pathway. The authors demonstrate that several enzymes corresponding to late steps of the proposed pathway exhibit either markedly reduced catalytic activity (PcTOT, Fig. 3e) or no detectable activity under the tested conditions (T9ox, T9αH-750C, T1βH-184C, BAPT, T3′NBT, and DBTNBT; Supplementary Figure 20). Collectively, these observations suggest that multiple bottlenecks exist in the terminal portion of the pathway and raise questions regarding the feasibility of complete paclitaxel biosynthesis in this species.

Furthermore, the additional metabolite analyses performed on leaves and bark from natural populations revealed no detectable paclitaxel in the vast majority of samples examined. According to Supplementary Figure 18, only two of twenty-seven samples were reported as positive. Given the extensive sampling effort, this result appears more consistent with the absence of a functional pathway than with a pathway that remains operational but is expressed only under specific conditions.

One of the two positive samples deserves particular attention. In this case, branches were treated with methyl jasmonate, and trace amounts of paclitaxel (<10 ng g^{-1}) were detected at the 48 h time point in only one of three sampled branches, whereas paclitaxel was no longer detected after 96 h. This observation is difficult to reconcile with de novo induction of paclitaxel biosynthesis by methyl jasmonate. An alternative explanation that should be considered is the possibility of trace contamination. At concentrations close to analytical detection limits, carryover can originate not only from the analytical instrumentation itself but also from laboratory consumables, extraction equipment, glassware, plasticware, or reagent preparation procedures. This issue is well recognized in trace analytical chemistry, including pesticide residue analysis, where stringent precautions are routinely implemented to prevent false-positive detections. For example, see:

https://www.fao.org/input/download/standards/378/cxg_040e.pdf

https://www.eurl-pesticides.eu/library/docs/allcrl/AqcGuidance_Sanco_2003_10476.pdf

Consequently, I do not believe that the current data provide sufficient evidence to support the conclusion that *P. chienii* retains "a complete paclitaxel biosynthetic pathway with a highly conditional residual capacity dependent on genotype-by-environment interactions." Rather, the combined biochemical and metabolomic evidence appears more consistent with the alternative hypothesis that *P. chienii* lacks a functional capacity for paclitaxel biosynthesis, with the rare positive detections requiring additional verification.

I therefore recommend that the authors revise their discussion and conclusions to more explicitly acknowledge this alternative interpretation. At a minimum, the manuscript should present the absence of functional paclitaxel biosynthesis as a plausible explanation supported by the available data, rather than favoring residual biosynthetic capacity as the primary conclusion.

Response: We sincerely thank you for this valuable assessment and constructive suggestions, which have helped us improve the rigor of the manuscript and ensure that our conclusions more accurately reflect the available evidence. We understand that your principal concern is that paclitaxel was detected at only trace levels in two of the27 examined samples and that these rare, poorly reproducible detections are insufficient to support the conclusion that *P. chienii* retains functional paclitaxel biosynthesis. We agree with this concern.

Your insightful suggestion prompted us to carefully re-evaluate the combined metabolomic and biochemical evidence, reconsider the overall logic and internal consistency of the manuscript, and make targeted revisions to the relevant text to ensure that our interpretation more accurately reflects both the strength and the limitations of the available evidence. In particular, we now present the absence of functional capacity for complete paclitaxel biosynthesis as a plausible interpretation supported by the available biochemical and metabolomic evidence, rather than favoring residual biosynthetic capacity as the primary conclusion. Weak residual activity under specific native conditions is retained only as an unresolved possibility requiring further

verification, and trace contamination is acknowledged as an alternative explanation for the isolated MeJA-associated detection. The main revisions are detailed below:

Pages 1, lines 33-35, "…a largely conserved genomic architecture associated with paclitaxel biosynthesis, its terminal pathway has undergone severe functional degradation and may have largely lost the functional capacity for paclitaxel production."

Pages 4, lines 98-102, "These results collectively indicate severe functional attenuation of the terminal paclitaxel biosynthetic pathway, consistent with substantial impairment of complete paclitaxel biosynthesis in *P. chienii*. This study advances our understanding of the evolutionary decay and metabolic diversification of complex specialized metabolic pathways beyond the Taxus genus."

Pages 13, lines 470-472, "independently detected on two LC-MS/MS platforms; the SCIEX Triple Quad 5500 LC-MS/MS spectrum is shown in Fig. 3f, and the SCIEX Triple Quad 7500 LC-MS/MS result is shown in Supplementary Fig. 18"

Pages 13, lines 474-489, "These rare detections do not establish residual paclitaxel biosynthetic activity, although such activity cannot be completely excluded. Moreover, given the low abundance and limited reproducibility of the MeJA-associated signal, trace contamination arising during sample handling, extraction, or instrumental analysis may represent an alternative explanation for this isolated MeJA-associated detection[50,51]. Independently, the markedly reduced catalytic activity of PcTOT and the absence of detectable activity for several downstream enzymes under the tested conditions support severe functional attenuation of the terminal pathway. Taken together, the combined biochemical and metabolomic evidence suggests that the absence of functional capacity for complete paclitaxel biosynthesis is a plausible interpretation, while weak residual activity under specific native conditions remains an unresolved possibility. The highly limited and inconsistent detection of paclitaxel may help explain the conflicting reports regarding its occurrence in *P. chienii* across

previous studies14-16. Resolving these alternatives and evaluating the possible contribution of G × E interactions to the observed variation in paclitaxel detection will require broader population-level and spatiotemporal sampling across multiple genotypes and environmental conditions, more extensive biological replication, stringent analytical verification, and further biochemical and in planta functional investigations."

Pages 14, lines 518-521, "Therefore, *P. chienii* may have lost the functional capacity for complete paclitaxel biosynthesis, although a residual, trace-level capacity under specific native conditions, possibly influenced by G × E interactions, cannot be excluded and remains to be verified"

We also carefully checked the manuscript for overall logical consistency and made minor wording and editorial corrections throughout to improve its clarity and scientific rigor. We sincerely thank you again for the valuable comments and suggestions, which have helped us further strengthen the manuscript.

R3Q2: Improve resolution of supplementary figure 15.

Response: We thank you for pointing this out. Supplementary Figure 15 has been regenerated at higher resolution. In addition, all figures are provided as vector files with this revision.

Response to REVIEWER COMMENTS:

Reviewer #3:

R3Q1: The authors have adequately addressed most of my comments, and I have no further remarks.

Response: We sincerely thank you for your time and expertise, and we grateful for your positive assessment of our revised manuscript. Your constructive feedback in the previous round was invaluable and has led to substantial improvements in our work.
