# Supplementary material for: Archipelago architecture and evolution of paclitaxel biosynthesis revealed by the Pseudotaxus chienii haplotype-resolved genome
Source: Nat Commun. 2026 Aug 26;17:10189. doi: 10.1038/s41467-026-77115-w (PMC13612588; doi:10.1038/s41467-026-77115-w)
Supplement: Supplementary file 4 [file 41467_2026_77115_MOESM3_ESM.pdf]

Inventory of Supporting Information

File Name: Supplementary Data 1

Description: BGCs in hap1 (Pca)

File Name: Supplementary Data 2

Description: BGCs in hap2 (Pcb)

File Name: Supplementary Data 3

Description: The genes in module of co-expression.

File Name: Supplementary Data 4

Description: Ka/Ks ratios, sequence similarity, and CDS sequence of orthologous gene pairs in the paclitaxel biosynthetic pathway between *T. mairei* and *P. chienii*

File Name: Supplementary Data 5

Description: Comparison of the *P. chienii* genome assembly with previously published gymnosperm genomes.

File Name: Supplementary Data 6

Description: Primers used in this study
